# Supplementary material for: All-Trans Retinoic Acid Stimulates Viral Mimicry, Interferon Responses and Antigen Presentation in Breast-Cancer Cells
Source: Cancers (Basel). 2020 May 6;12(5):1169. doi: 10.3390/cancers12051169 (PMC7281473; doi:10.3390/cancers12051169)
Supplement: Supplementary file 1 [file cancers-12-01169-s001.zip › cancers-776836 supplementary/Suppl. INFO_Cancers _final.pdf]

SUPPLEMENTARY INFORMATION

Title: All-trans retinoic acid stimulates viral mimicry, interferon responses and antigen presentation in breast-cancer cells

Marco Bolis[1,2], Gabriela Paroni[1], Maddalena Fratelli[1], Arianna Vallerga[1], Luca Guarrera[1], Adriana Zanetti[1], Mami Kurosaki[1], Silvio Ken Garattini[3], Maurizio Gianni'[1], Monica Lupi[4], Linda Pattini[5], Maria Monica Barzago[1], Mineko Terao[1] and Enrico Garattini[1]

[1]Laboratory of Molecular Biology, Istituto di Ricerche Farmacologiche Mario Negri IRCCS, via La Masa 19, 20156 Milano Italy. [2]Functional Cancer Genomics Laboratory, Institute of Oncology Research; Bioinformatics Core Unit, SIB-Swiss Institute of Bioinformatics; USI, University of Southern Switzerland. Bellinzona, Switzerland. [3]DAME, Dipartimento di Area Medica Università degli Studi di Udine. Department of Oncology, Azienda Ospedaliera di Udine, Udine, Italy. [4] Department of Oncology, Istituto di Ricerche Farmacologiche Mario Negri IRCCS, via La Masa 19, 20156 Milano Italy. [5] Department of Electronics, Information and Bioengineering, Politecnico di Milano, Milano

Supplementary Methods	pages 2-4
Legends to Supplementary Tables S1-S4	pages 4-5
Supplementary Figures S1-S15	pages 6-20
Original Western blots of Figures 5-7	pages 21-31

SUPPLEMENTARY METHODS

Cell lines: The table below summarizes the characteristics and the source of the cell lines used throughout the study:

**Cell name**	**Gender**	**Phenotype**	**Source**
AU565	F	Luminal ($HER2^+$)	ATCC
BT20	F	Basal (triple-negative)	ATCC
BT474	F	Luminal ($ER^+/HER2^+$)	ATCC
BT483	F	Luminal (ER^+)	ATCC
BT549	F	Basal (triple-negative)	ATCC
CAL-120	F	Basal (triple-negative)	DSMZ
CAL-148	F	Basal (triple-negative)	DSMZ
CAL-51	F	Basal (triple-negative)	DSMZ
CAL-851	F	Basal (triple-negative)	DSMZ
CAMA1	F	Luminal (ER^+)	ATCC
DU4475	F	Basal (triple-negative)	ATCC
EFM192A	F	Luminal ($ER^+/HER2^+$)	DSMZ
EFM19	F	Luminal ($ER^+/HER2^+$)	DSMZ
HCC-1143	F	Basal (triple-negative)	ATCC
HCC-1187	F	Basal (triple-negative)	ATCC
HCC-1395	F	Basal (triple-negative)	ATCC
HCC-1419	F	Luminal ($HER2^+$)	ATCC
HCC-1428	F	Luminal (ER^+)	ATCC
HCC1500	F	Luminal (ER^+)	ATCC
HCC-1569	F	Basal (triple-negative)	ATCC
HCC-1599	F	Basal (triple-negative)	ATCC
HCC-1806	F	Basal (triple-negative)	ATCC
HCC-1937	F	Basal (triple-negative)	ATCC
HCC-1954	F	Basal (triple-negative)	ATCC
HCC-202	F	Luminal ($HER2^+$)	ATCC
HCC-2218	F	Luminal ($HER2^+$)	ATCC
HCC-38	F	Basal (triple-negative)	ATCC
HCC-70	F	Basal (triple-negative)	ATCC
HDQP1	F	Basal (triple-negative)	DSMZ
Hs281T	F	Basal (triple-negative)	ATCC
Hs343T	F	Basal (triple-negative)	ATCC
Hs578T	F	Basal (triple-negative)	ATCC
KPL1	F	Luminal (ER^+)	DSMZ
MB-157	F	Basal (triple-negative)	ATCC
MCF7	F	Luminal (ER^+)	ATCC
MDA-MB-157	F	Basal (triple-negative)	ATCC
MDA-MB-134VI	F	Luminal (ER^+)	ATCC
MDA-MB-175VII	F	Luminal (ER^+)	ATCC

MDA-MB-231	F	Basal (triple-negative)	ATCC
MDA-MB-361	F	Luminal (ER^+/$HER2^+$)	ATCC
MDA-MB-415	F	Luminal (ER^+)	ATCC
MDA-MB-435S	F	Basal (triple-negative)	ATCC
MDA-MB-436	F	Basal (triple-negative)	ATCC
MDA-MB-453	F	Basal (triple-negative)	ATCC
MDA-MB-468	F	Basal (triple-negative)	ATCC
SK-BR-3	F	Luminal ($HER2^+$)	ATCC
T47D	F	Luminal (ER^+)	ATCC
UACC-812	F	Luminal ($ER2^+$/$HER2^+$)	ATCC
ZR75.1	F	Luminal (ER^+)	ATCC
ZR75.30	F	Luminal (ER^+/$HER2^+$)	ATCC

ATCC = American Type Culture Collection; DSMZ = Deutsche Sammlung von Mikroorganismen und Zellkulturen; SIGMA = Sigma-Aldrich. The cell-lines marked in yellow are the 16 cell-lines used to perform the RNA-seq experiments following treatment with ATRA. During the course of the entire study, all the cell lines were authenticated by constantly checking the morphology and the growth doubling time. In addition, all the cell lines were mycoplasma free, as indicated by periodic PCR assays performed on the cell conditioned medium, using the following nucleotide mycoplasma-recognizing primers (forward: 5’TGCACCATCTGTCACTCTGTTAACCTC3’; reverse: 5’ACTCCTACGGGAGGCAGCAGTA3’).

Primary breast cancer specimens

The primary mammary tumour specimens used for the studies described in Fig. 4D were obtained from 9 distinct patients whose relevant clinical information are summarized below.

Sample No.	Patient's code	ER %	PG %	HER2 Hercep Test	Ki 67 %	classification	Stage	Grade
22	14089_3_M_5	0%	0%	1+	50%	TN	pT2	G2
23	14343_3_M_8	0%	0%	0	40%	TN	pT3	G3
31	15542_3_1	0%	0%	1+	80%	TN	pT1c	G3
36	16536_3_M_1	90%	90%	0	12%	LumA	pT2N1a	G2
41	16440_3_M_1	90%	90%	1+	8%	LumA	pT1b	G2
44	8001_3_M_1	90%	90%	1+	10%	LumA	pT1c	G2
61	19094_3_M_3	80%	30%	2+	15%	Lum B	pT1c	G2
64	18993_3_M_3	80%	80%	1+	25%	Lum B	pT1c	G3
67	19385_3_M_4	70%	5%	3+	10%	HER2/RAR	pT1c	G3

Legend: ER = estrogen receptor; PG = progesterone receptor; HER2 = HER2 positive; TN = triple-negative; LumA = Luminal A; LumB = Luminal B; HER2/RAR: HER2 positive with co-amplification of the HER2 and RARα genes.

Polymerase Chain Reaction experiments: The expression of the following transcripts was evaluated with the use of commercially available taqman assays according to the instructions of the manufacturer (ThermoFisher Scientific): Dihydroorotate dehydrogenase (DHODH, NM_001361.4; assay ID-Hs00361406_m1); Minichromosome maintenance complex component 6 (MCM6, NM_005915.5; assay ID-Hs00195504_m1); Minichromosome maintenance complex component 2 (MCM2, NM_004526.3; assay ID-Hs01091568_g1); Poly (ADP-ribose) polymerase family, member 14 (PARP14, NM_017554.2; Hs00393814_m1).

Identification of genetic perturbations proportional to ATRA-sensitivity and generation of ATRA-sensitivity associated gene interaction networks: After a first phase of differential expression analysis, we determined for each gene, the correlation between ATRA-induced fold changes across cell-lines and the respective ATRA sensitivity scores. Subsequently, we filtered out genes with either Pearson's or Spearman's correlation coefficients associated p-values lower than 0.01. We adopted both metrics in order to obtain more inclusive results. We further discarded genes showing a coefficient of variation across cell-lines being lower than 0.5 and obtained a final list consisting of 754 genes, whose perturbations are associated with ATRA responsiveness. Finally, we constructed a protein-protein interaction network using the *STRING* database and discarded all elements with less than three edges. A scheme of the strategy used for the analysis of the data is available in Supplementary Figure S2.

LEGENDS TO SUPPLEMENTARY TABLES

Supplementary Table S1 *Genes up- and down-regulated by ATRA in breast-cancer cell-lines* The indicated cell-lines were treated with DMSO or ATRA (1 μM) for 24 hours. Whole-genome gene-expression analysis was performed by *RNA-seq*. The table contains the list of 754 genes significantly up- and down-regulated by ATRA and whose ATRA-dependent expression is quantitatively and significantly correlated with the *ATRA-score*. The expression data are shown as the $\log_2$ of the ATRA/DMSO fold-change and the values calculated derive from 3 biological replicates. The values corresponding to the following correlation indexes are provided: pRHO = p-value of the Spearman's correlation between ATRA/DMSO fold-change and *ATRA-score*; RHO = Spearman's correlation coefficient between ATRA/DMSO fold-change and *ATRA-score*; pR = p-value of the Pearson's correlation between ATRA/DMSO fold-change and *ATRA-score*; R = Pearson's correlation coefficient between ATRA/DMSO fold-change and *ATRA-score*; CV = Coefficient of variation.

Supplementary Table S2 *Enrichment analyses of the gene-sets up- and down-regulated by ATRA which are quantitatively associated with ATRA sensitivity in breast-cancer cell-lines* Each breast-cancer cell-line was treated with vehicle (DMSO) or ATRA (10^{-6} M) for 24 hours. Total RNA was extracted and subjected to *RNA-seq* analysis. The gene-sets up- or down-regulated by ATRA and whose significance is proportional to ATRA-sensitivity were subjected to GSEA (Gene Set Enrichment Analysis) using the HALLMARK and the CURATED datasets. The FDR (False Discovery Rate) values and the ATRA-dependent up-or down-regulation are indicated.

Supplementary Table S3 *Identification of the single genes and gene-sets up- and down-regulated by ATRA in cell-lines characterized by low, intermediate and high sensitivity to the anti-proliferative action of the retinoid* Each breast-cancer cell-line was treated with vehicle (DMSO) or ATRA (10^{-6} M) for 24 hours. Total RNA was extracted and subjected to *RNA-seq* analysis. The individual genes

and gene-sets up- or down-regulated by ATRA in cell-lines characterized by low, intermediate and high sensitivity to the anti-proliferative effects of the retinoid. GSEA (Gene Set Enrichment Analysis) was performed using the HALLMARK datasets. The FDR (False Discovery Rate) values and the ATRA-dependent up-or down-regulation are indicated.

Supplementary Table S4 *Enrichment analyses of the gene-sets up- and down-regulated by ATRA in HCC-1599 xenografts* Three separate SCID mice/experimental group were transplanted sub-cutaneously with *HCC-1599* cells. Animals were administered two daily doses of vehicle or ATRA (15 mg/kg) orally. RNA was extracted from the isolated tumor tissues and subjected to microarray gene-expression analysis. The gene-sets up- or down-regulated by ATRA were subjected to GSEA (Gene Set Enrichment Analysis) using the HALLMARK and the CURATED datasets. The FDR values and the ATRA-dependent up-or down-regulation are indicated.

Supplementary Table S5 *Effect of ATRA on the expression of ENV RNAs in breast cancer cell-lines* Each breast-cancer cell-line was treated with vehicle (DMSO) or ATRA (10^{-6} M) for 24 hours. Total RNA was extracted and subjected to *RNA-seq* analysis. The expression levels of ENV RNAs were determined. The > $5x10^{6}$ ENV-derived small RNAs are grouped in 42 classes. The p-values of the overall up- and down-regulation of each class and the entire series of ENV-derived RNAs identified in each cell-line is indicated.

Supplementary Table S6 *Effect of ATRA on the expression of interferon genes in breast cancer cell-lines* Each breast-cancer cell-line was treated with vehicle (DMSO) or ATRA (10^{-6} M) for 24 hours. Total RNA was extracted and subjected to *RNA-seq* analysis. The expression levels of the transcripts encoding the indicated interferon (IFN) species were determined. Each value is the mean of 3 replicate cultures treated with vehicle (DMSO) or ATRA.

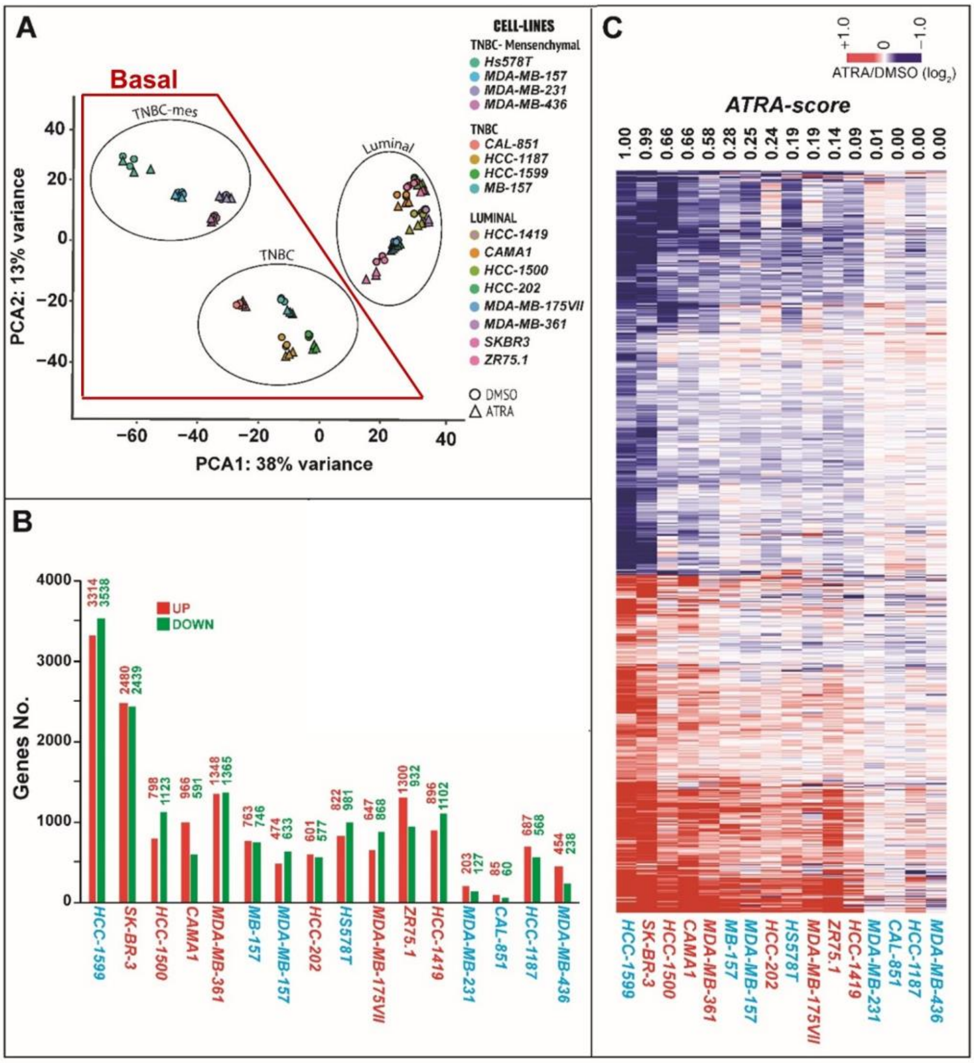

Supplementary Figure S1 *Gene expression profiles of the breast-cancer cell-lines following exposure to ATRA*

Three replicate cultures of the indicated and logarithmically growing cell-lines were treated with vehicle (DMSO) or ATRA (1 μM) for 24 hours. RNA was extracted and subjected to *RNA-seq* analysis. (**A**) The diagram illustrates a bi-dimensional principal component analysis (PCA) of the *RNA-seq* data obtained in each cell-line exposed to DMSO or ATRA. The gene-expression data clearly separate *Basal* and *Luminal* cell-lines. *Basal* cell lines are further divided into two subgroups, i.e. *TNBC* and *TNBC-mes*, which recapitulate their phenotypic and morphological characteristics. ATRA-treatment does not cause transitions across the 3 groups of cell-lines. *TNBC* = triple-negative breast-cancer; *TNBC-mes* = triple-negative breast-cancer with a mesenchymal phenotype. (**B**) The column graph indicates the number of genes selectively up- (red) or down-regulated (green) by ATRA in each cell-line. The cell-lines are ranked from left to right according to their decreasing sensitivity to ATRA (decreasing *ATRA-score* value). *Luminal* cell-lines are indicated in red, while *Basal* cell-lines are marked in blue. The inset shows the linear correlation between the *ATRA-score* values and the total number of up- and down-regulated genes determined in each cell-line. The calculated *r* value for the correlation is shown. (**C**) The heat-map shows the 754 genes whose up-or down-regulation is proportional to ATRA-sensitivity. The cell-lines are ranked from left to right according to their decreasing sensitivity to ATRA. The *ATRA-score* values are shown above the heat-map. *Luminal* cell-lines are indicated in red, while *Basal* cell-lines are marked in blue.

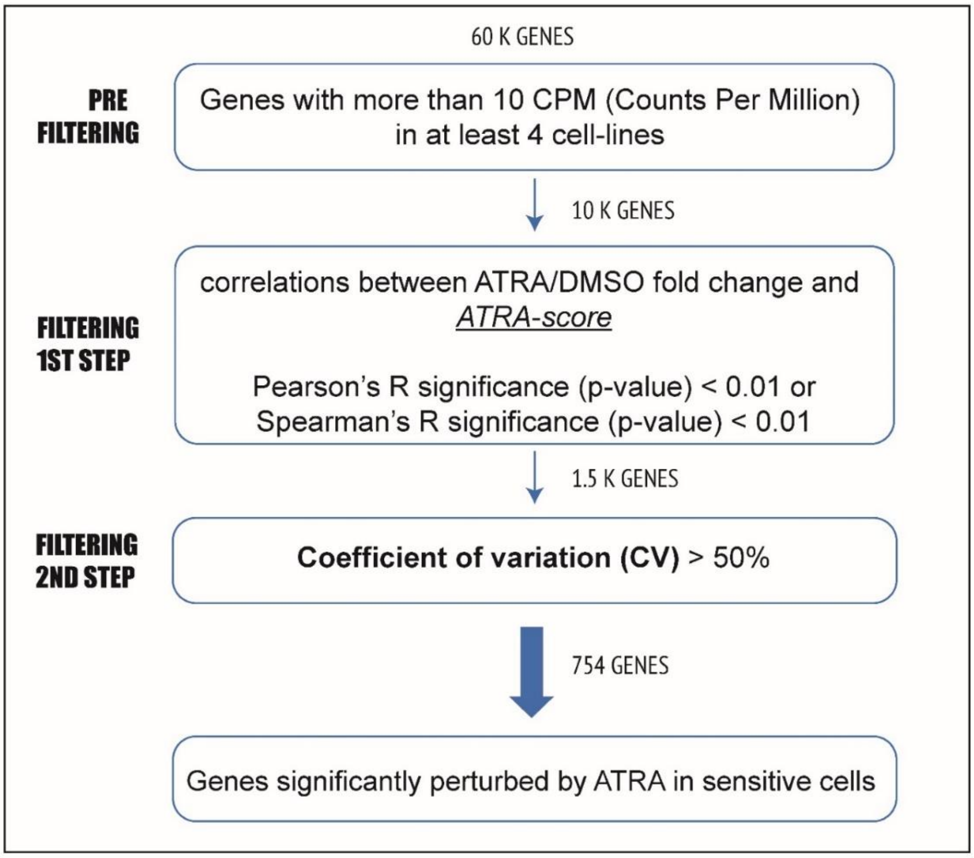

Supplementary Figure S2 *Identification of the genes differentially regulated by ATRA in sensitive cell-lines*

Biological triplicates of 16 breast cancer cell-lines were treated with vehicle (DMSO) or ATRA (10^{-6} M) for 24 hours. Total RNA was extracted and subjected to *RNA-seq* analysis. The flow-chart illustrates the computational approach applied to define the 754 genes whose expression is significantly perturbed by ATRA and quantitatively correlated with cell-line sensitivity to the anti-proliferative activity of ATRA (*ATRA-score* value).

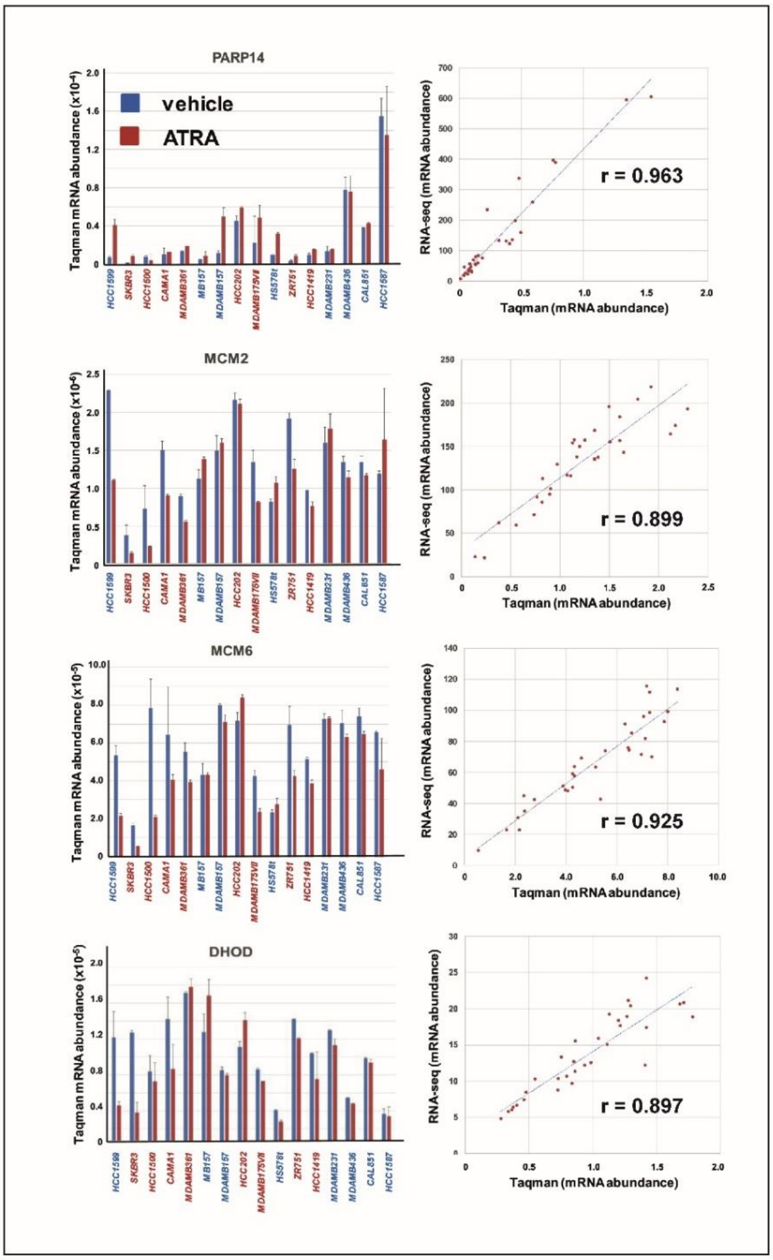

Supplementary Figure S3 *Validation of the RNA-seq data*
The *RNA-seq* data were validated by Taqman analysis of the 4 indicated genes in all the cell-lines considered. The indicated cell-lines were treated with vehicle (DMSO) or ATRA (10^{-6} M) for 24 hours. Total RNA was extracted and subjected to Taqman analysis using specific oligonucleotide probes targeting the PARP14, MCM2, MCM6 and DHODH mRNAs. Each column represents the Mean $\pm$ SD of 3 replicate cultures. The scatter plots on the right show the correlation between the Taqman and the *RNA-seq* data in each cell-line exposed to vehicle or ATRA (n = 32). The r value of each correlation is shown. PARP14 = Poly (ADP-ribose) polymerase family, member 14; MCM2 = Minichromosome maintenance complex component 2; MCM6 = Minichromosome maintenance complex component 6; DHODH = dihydroorotate dehydrogenase.

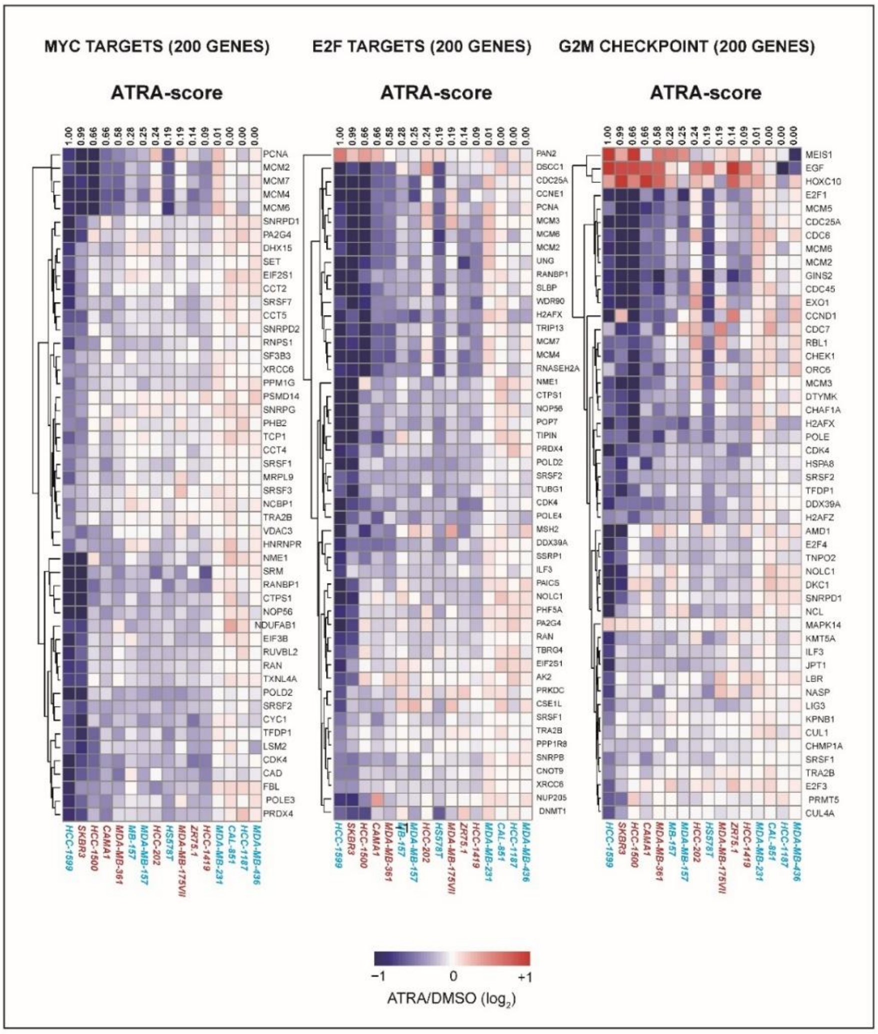

Supplementary Figure S4 *Effects of ATRA on the genes belonging to the "MYC Targets", "E2F Targets" and "G2M Checkpoint" gene-sets*

The indicated cell lines were treated with vehicle (DMSO) or ATRA (1 μM) for 24 hours. Total RNA was extracted and subjected to *RNA-seq* analysis. The heat-maps show the effects exerted by ATRA on the expression of the genes belonging to the HALLMARK "*MYC Targets*", *"E2F Targets"* and *"G2M Checkpoint"* gene-sets. The data are expressed as the ATRA/DMSO fold change. The "*MYC Targets*" gene-set is a merged version of the HALLMARK "*MYC Targets V1*" and the "*MYC Targets V2*" gene-sets. Each of the "*MYC Targets*" *"E2F Targets"* and *"G2M Checkpoint"*gene-sets consist of 200 genes and the heat-maps show the 50 top-ranking genes significantly correlated to the *ATRA-score* of the indicated cell-lines. The cell-lines are ordered according to their *ATRA-score* from left to right and the *ATRA-score* values are shown on the top. The cell-lines marked in blue have a basal phenotype, while the cell-lines marked in red are endowed with a luminal phenotype.

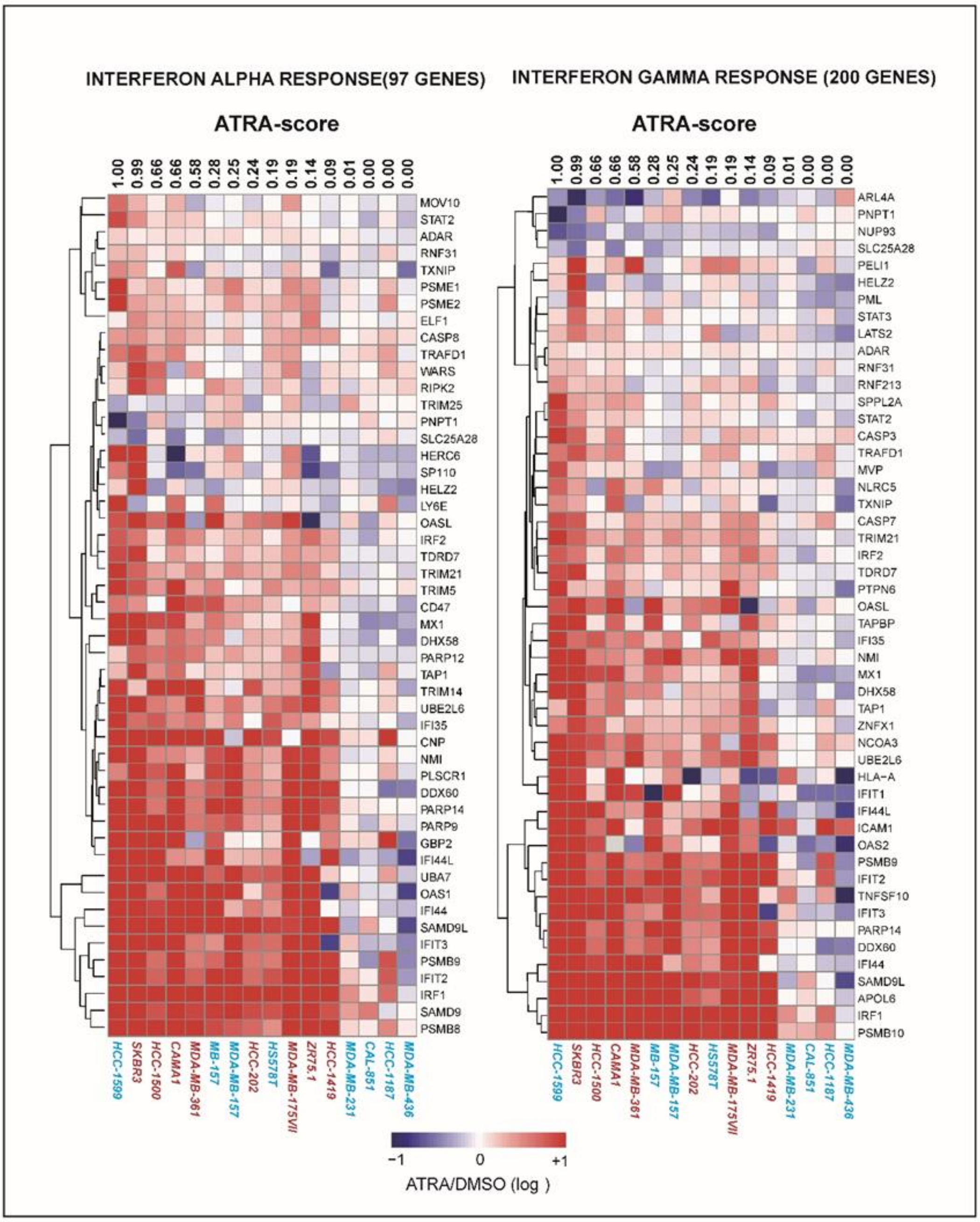

Supplementary Figure S5 *Effects of ATRA on the genes belonging to the "Interferon Alpha Response" and "Interferon Gamma Response" gene-sets*

The indicated cell lines were treated with vehicle (DMSO) or ATRA (1 μM) for 24 hours. Total RNA was extracted and subjected to *RNA-seq* analysis. The heat-maps show the effects exerted by ATRA on the expression of the genes belonging to the "*Interferon Alpha Response*" and *"Interferon Gamma Response"* HALLMARK gene-sets. The data are expressed as the ATRA/DMSO fold change. The "*Interferon Alpha Response*" and *"Interferon Gamma Response"* gene-sets consists of 97 and 200 genes, respectively, and the heat-maps show the 50 top-ranking genes significantly correlated to the *ATRA-score* of the indicated cell-lines. The cell-lines are ordered according to their *ATRA-score* from left to right and the *ATRA-score* values are shown on the top. The cell-lines marked in blue have a basal phenotype, while the cell lines marked in red are endowed with a luminal phenotype.

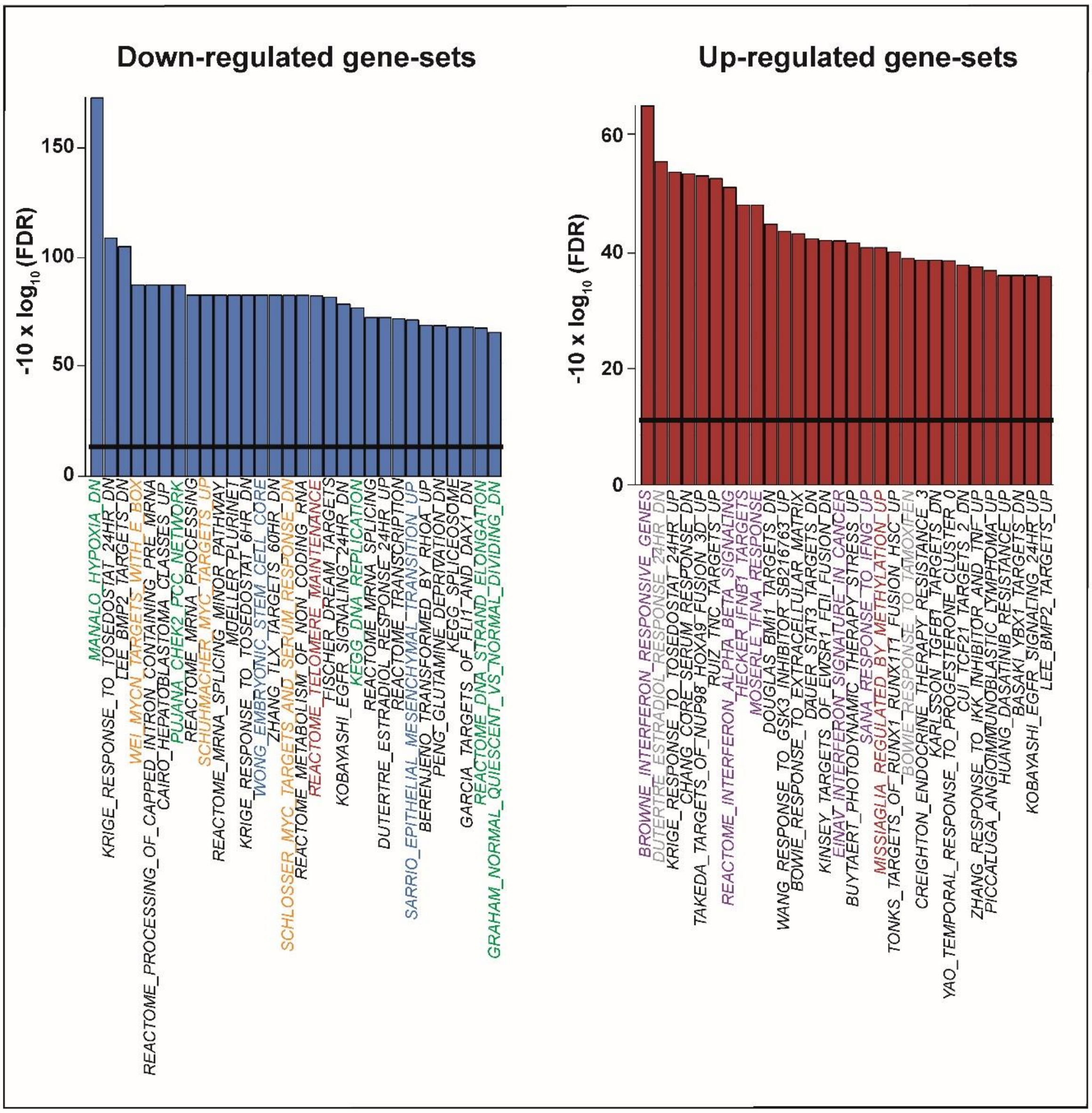

Supplementary Figure S6 *Gene-network enrichment analysis of the RNA-seq results obtained in breast-cancer cell-lines*

Gene-network enrichment analysis was performed on the 754 genes whose up-or down-regulation is proportional to ATRA-sensitivity, using the CURATED gene-sets. The top 30 down-regulated gene-networks are shown by the blue-column graph on the left, while the top 30 up-regulated gene-networks are illustrated by the red-column graph on the right. The horizontal line indicates the FDR threshold value considered. Gene-sets involved in in the control of: Green = proliferation/cell-cycle; Brown = histone/chromatin organization and *"Missiaglia-Regulated-by-Methylation-UP"*; Orange = Myc-pathway; Grey = estrogen-responses; Violet = interferon/inflammatory responses.

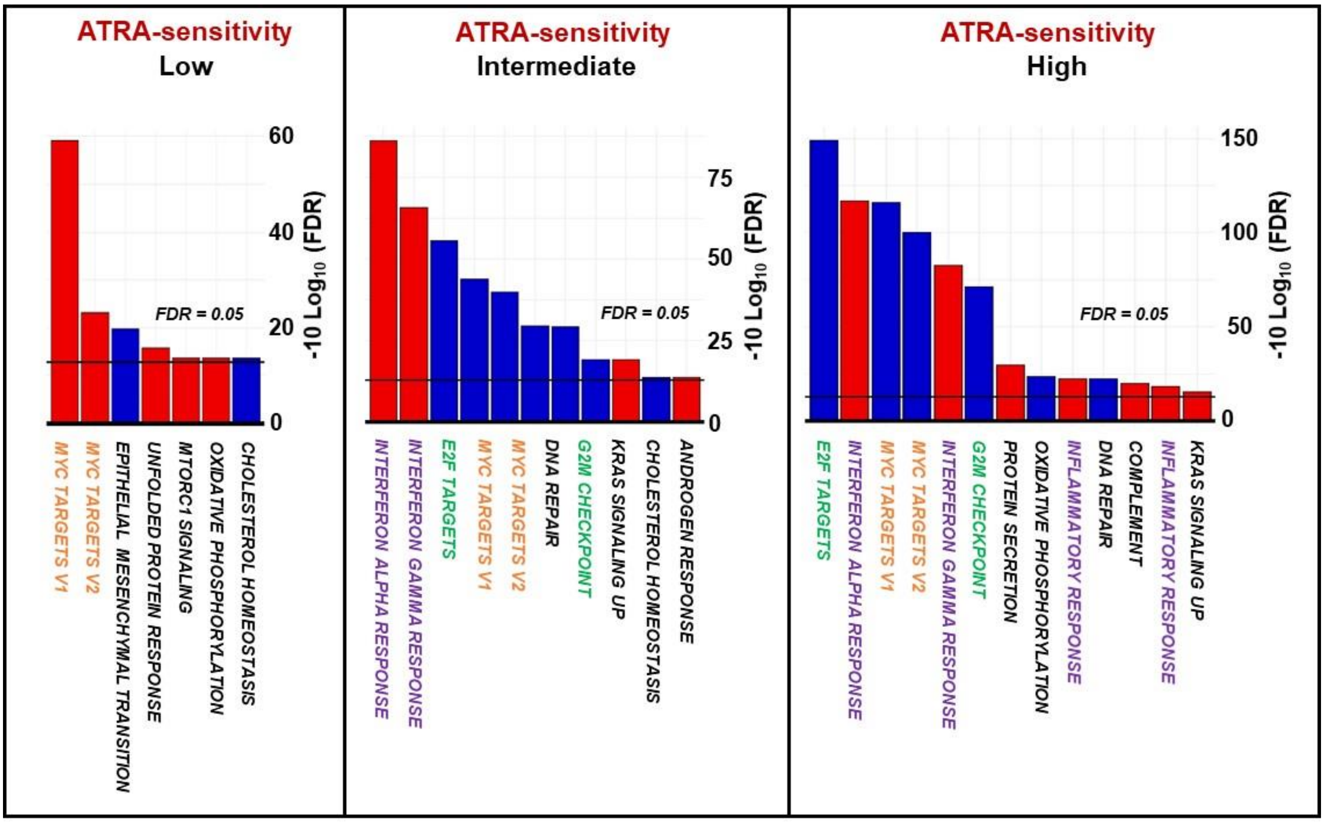

Supplementary Figure S7 *Gene-network enrichment analysis of the RNA-seq results obtained in breast-cancer cell-lines*

Gene-network enrichment analysis was performed on all the genes up- and down-regulated in the cell-lines characterized by low, intermediate and high sensitivity to the anti-proliferative action of ATRA, using the HALLMARK gene-sets. The blue and red columns indicate the gene-sets collectively down- and up-regulated by ATRA, respectively. The horizontal line indicates the FDR (False Discovery Rate) threshold value considered. Green = Gene-sets involved in the control of proliferation/cell-cycle; Orange = Gene-sets involved in the control of the Myc-pathway; Violet = Gene-sets involved in interferon/inflammatory responses.

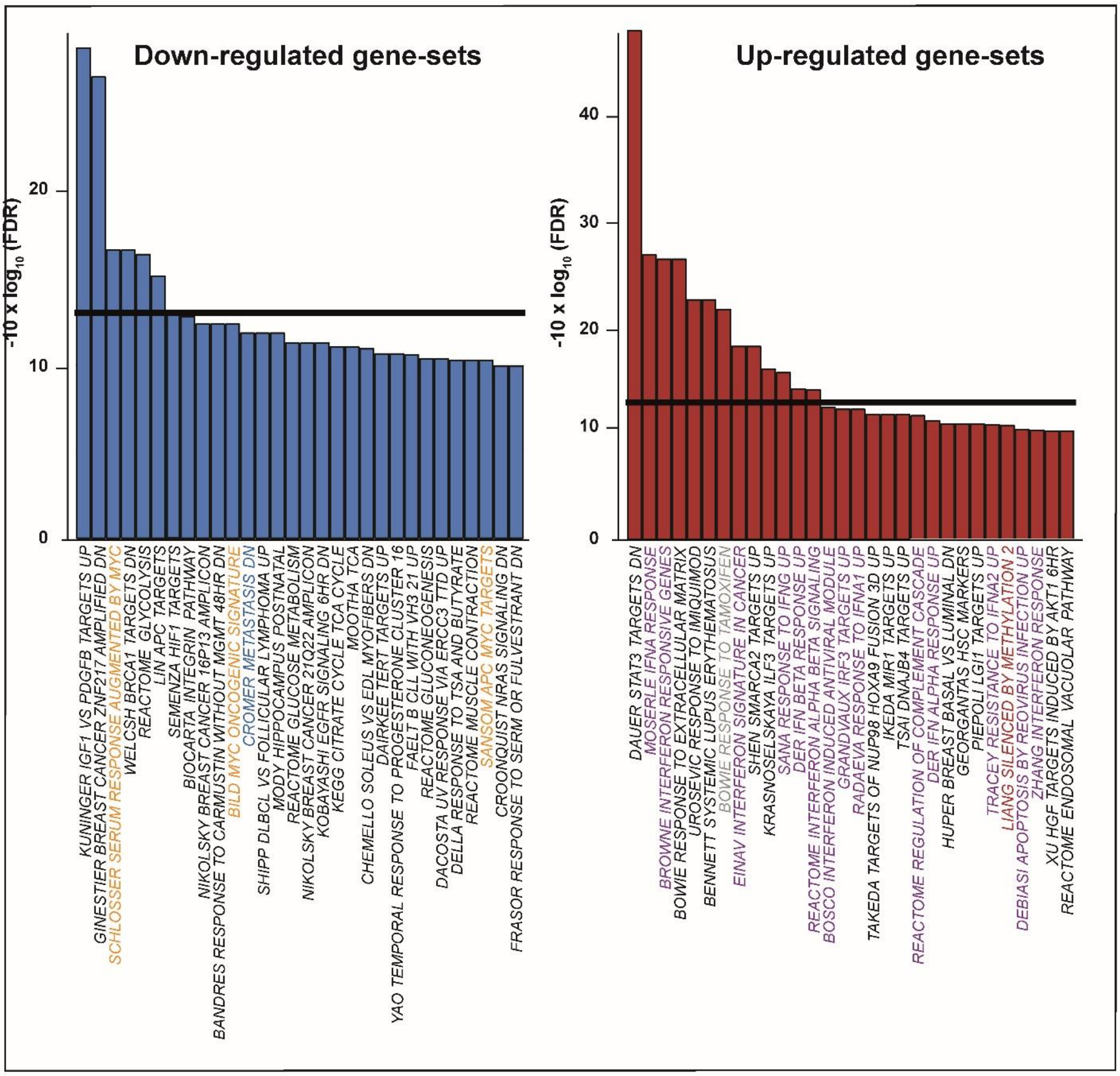

Supplementary Figure S8 *Gene-network enrichment analysis of the RNA-seq results obtained in HCC-1599 xenografts*

HCC-1599 cells were transplanted in nude mice subcutaneously. Xenografted animals were treated with vehicle or ATRA according to the scheme illustrated in Centritto *et al*. (EMBO Mol Med 7, 950–972). Twenty four hours following the last treatment, the *HCC-1599* xenografts of 3 ATRA-treated and 3 vehicle treated animals were subjected to whole-genome gene-expression microarray analysis. Genes significantly up- and down-regulated by ATRA were subjected to gene-network enrichment analysis using the CURATED gene-sets. The top 30 down-regulated gene-sets are shown by the blue-column graph on the left, while the top 30 up-regulated gene-sets are illustrated by the red-column graph on the right. The horizontal line indicates the FDR (False Discovery Rate) threshold value considered. Orange = genes involved in the Myc-pathway; Blue = genes involved in the metastatic process; Brown = genes involved in the DNA methylation process; Violet = genes involved in interferon responses; Grey = genes involved in the estrogen-receptor pathway.

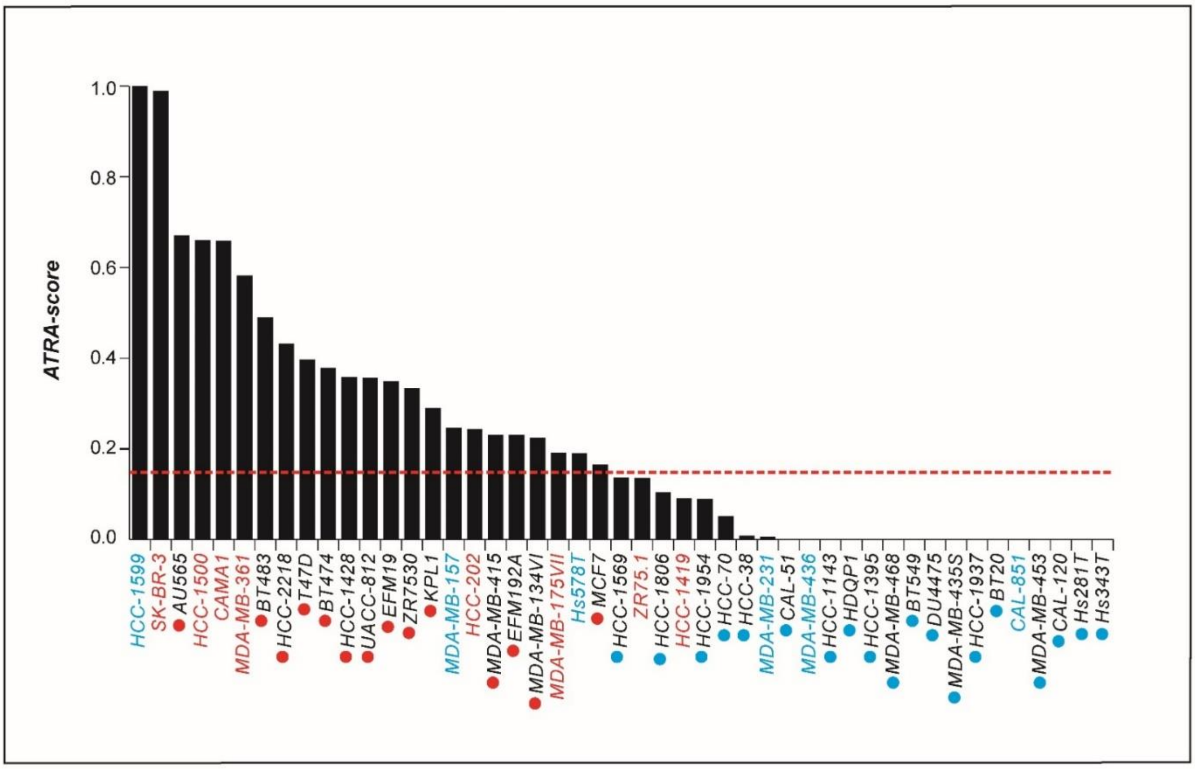

Supplementary Figure S9 *Sensitivity of breast cancer cell-lines to the anti-proliferative activity of ATRA*

The column diagram illustrates the experimental *ATRA-score* values calculated for the indicated cell-lines. The 14 cell-lines belonging to our experimental panel are shown in blue (basal cell-lines) and red (luminal cell-lines). The basal or luminal phenotype of all the other cell-lines is indicated by a blue and a red dot, respectively. The dotted red line represents the threshold *ATRA-score* value (0.17) used to separate the cell-lines binomially in two groups characterized by high and low ATRA-sensitivity.

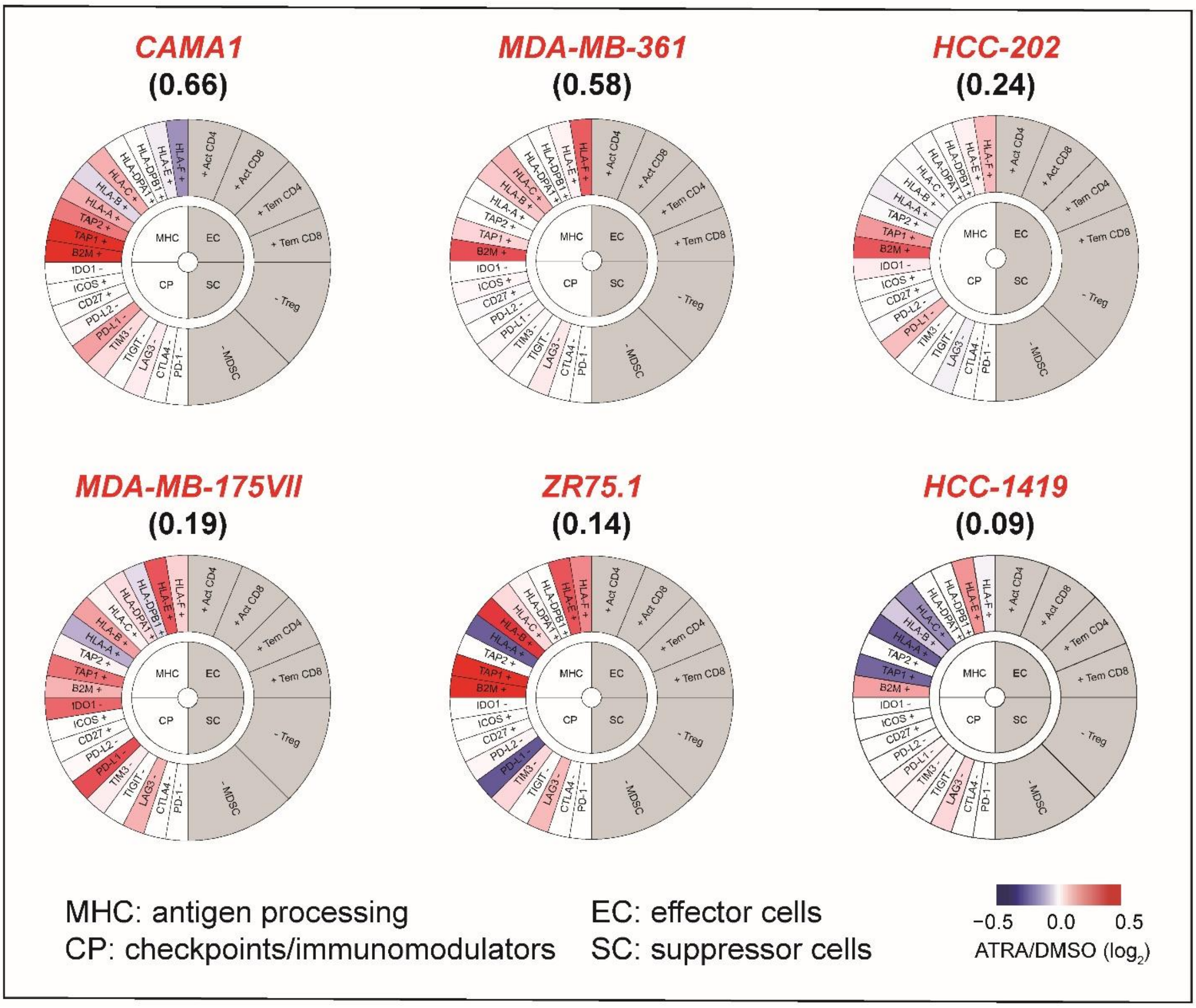

Supplementary Figure S10 *Effects of ATRA on the immunophenograms of luminal cell-lines*
The indicated luminal cell lines were treated with vehicle (DMSO) or ATRA (1 μM) for 24 hours. Total RNA was extracted and subjected to *RNA-seq* analysis. The circles show the effects exerted by ATRA on the immunophenograms derived from the *RNA-seq* data. The *ATRA-score* of each cell-line is indicated in parenthesis.

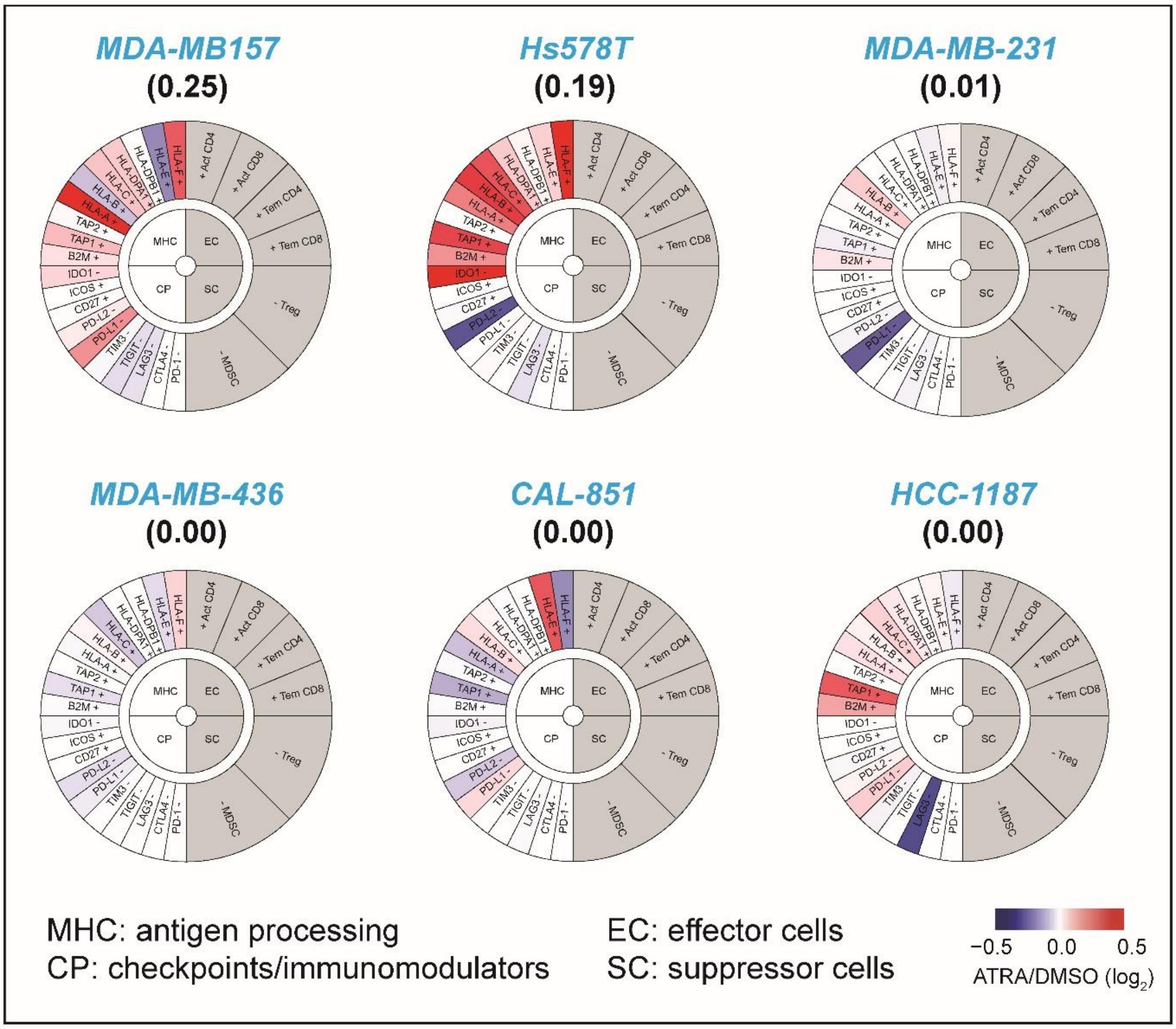

Supplementary Figure S11 *Effects of ATRA on the immunophenograms of basal cell-lines*
The indicated basal cell lines were treated with vehicle (DMSO) or ATRA (1 µM) for 24 hours. Total RNA was extracted and subjected to *RNA-seq* analysis. The circles show the effects exerted by ATRA on the immunophenograms derived from the *RNA-seq* data. The *ATRA-score* of each cell-line is indicated in parenthesis.

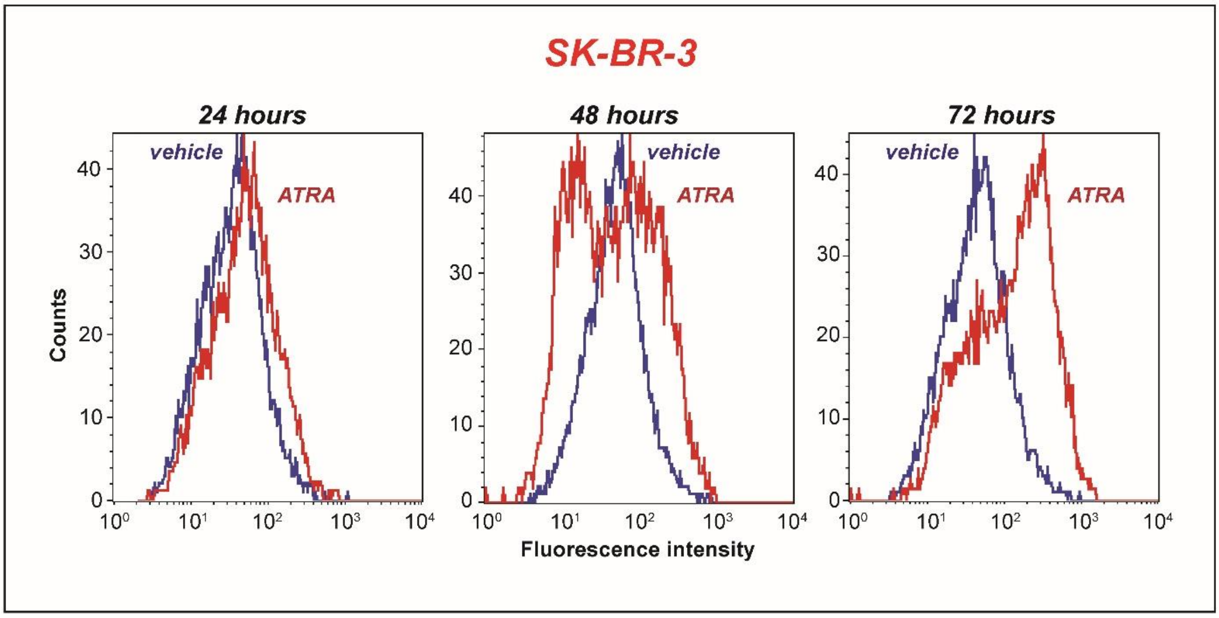

Supplementary Figure S12 *Effects of ATRA on the surface expression of HLA class I antigens in SK-BR-3 cells*

The panel illustrates the FACS (Fluorescence Activated Cell Sorter) analyses of the HLA molecules obtained in retinoid sensitive *SK-BR-3* cells exposed to ATRA for the indicated time frames.

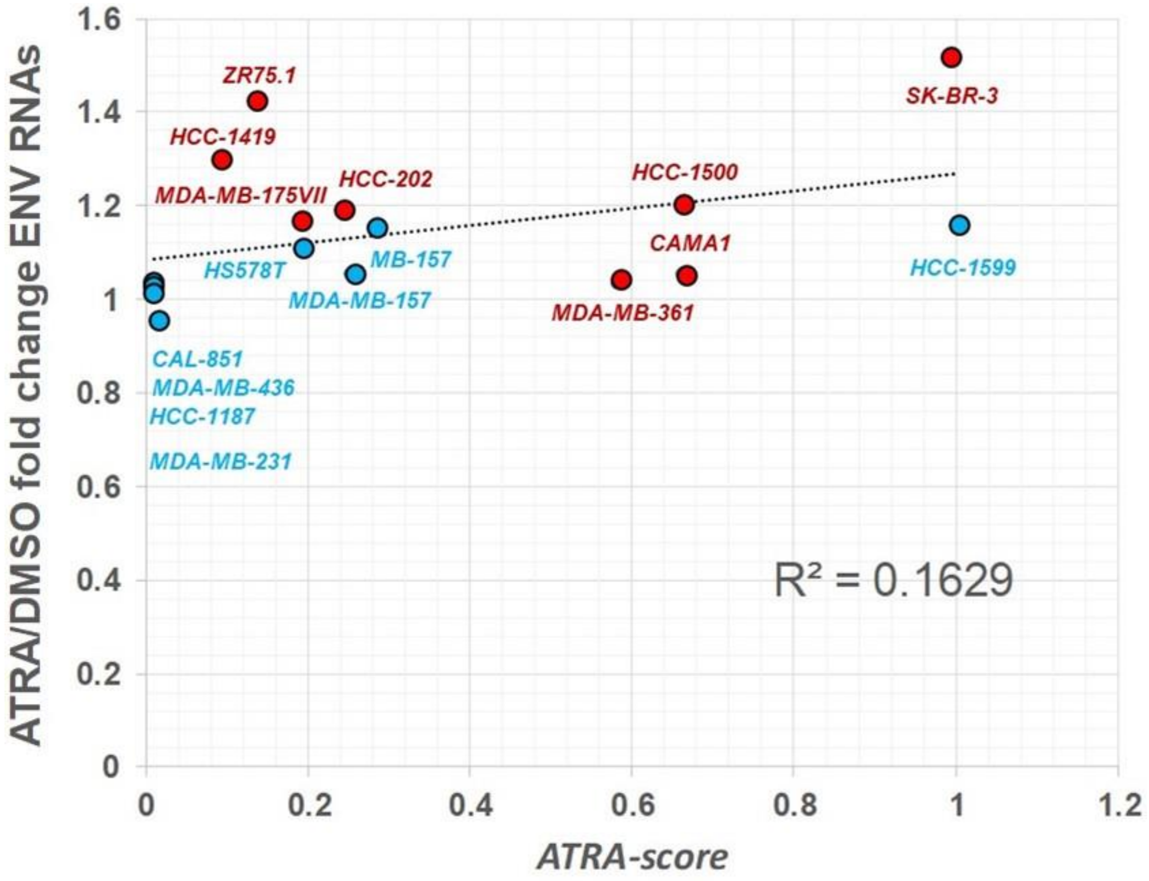

Supplementary Fig. S13 *Effects of ATRA on the endogenous retroviral elements in breast cancer cell-lines*

The diagram illustrates the correlations between the effects exerted by ATRA on ENV derived mRNAs and the *ATRA-scores* determined in luminal (red) and basal (blue) cell-lines, as indicated. ATRA/DMSO fold change ENV RNAs = fold change of the median expression values of the single ENV RNAs determined in each cell line following treatment with ATRA (1 µM) or vehicle (DMSO) for 24 hours. The median fold changes are expressed in linear values and were calculated from the logarithmic values shown in Figure 5B and Supplementary Table S5. The R^2 values of the correlations are indicated. The R^2 value of the correlation is indicated.

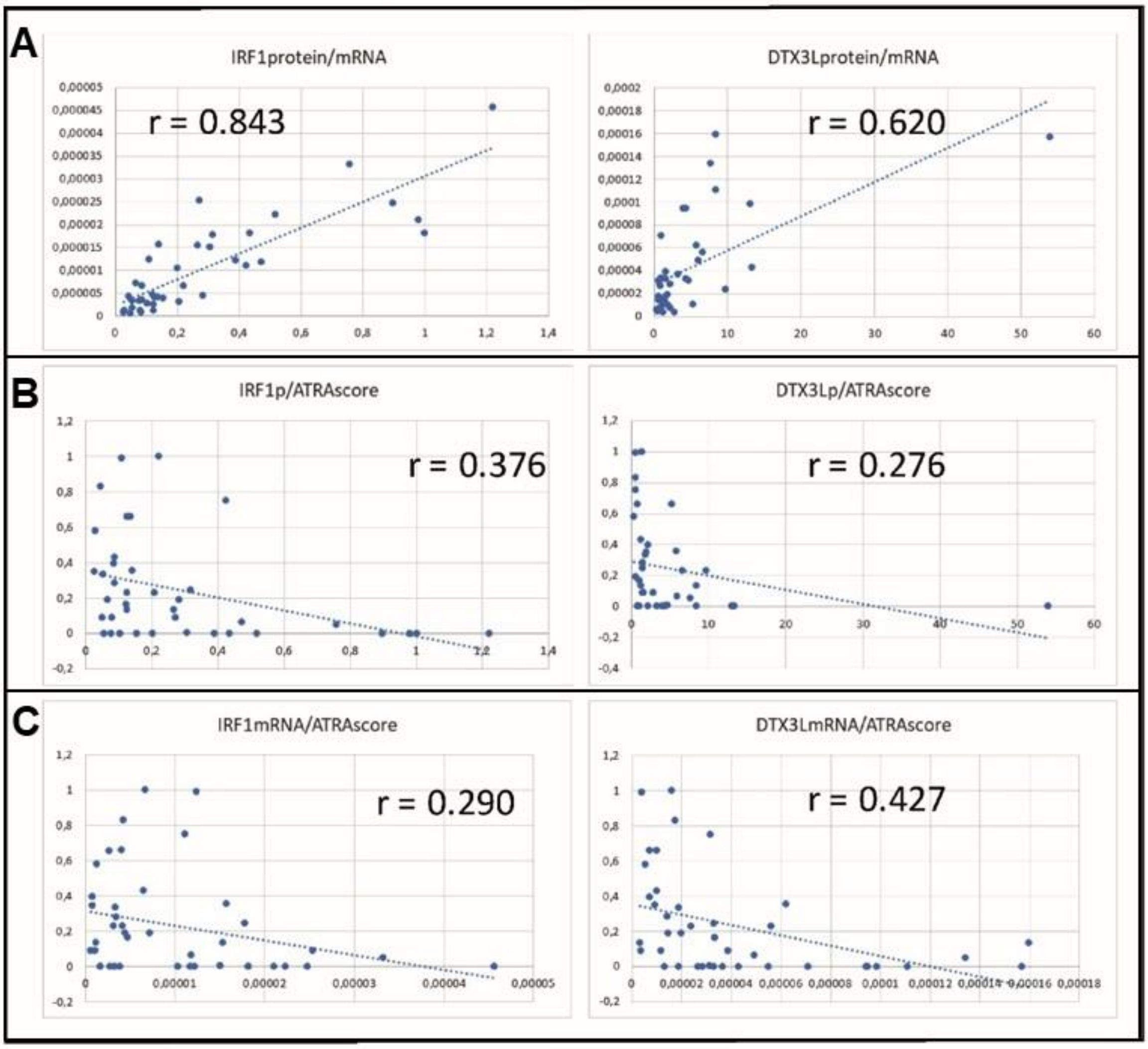

Supplementary Fig. S14 *IRF1 and DTX3L correlations*
The levels of IRF1 and DTX3L proteins as well as mRNAs were determined in 41 breast-cancer cell-lines profiled for their sensitivity to ATRA with the use of the *ATRA-score*. (**A**) The diagrams show the linear correlation plots of IRF1 and DTX3L proteins with the corresponding mRNAs. (**B**) The diagrams show the linear correlation plots of IRF1 and DTX3L proteins with the *ATRA-score* of each cell-line. (**C**) The diagrams show the linear correlation plots of IRF1 and DTX3L mRNAs with the *ATRA-score* of each cell-line.

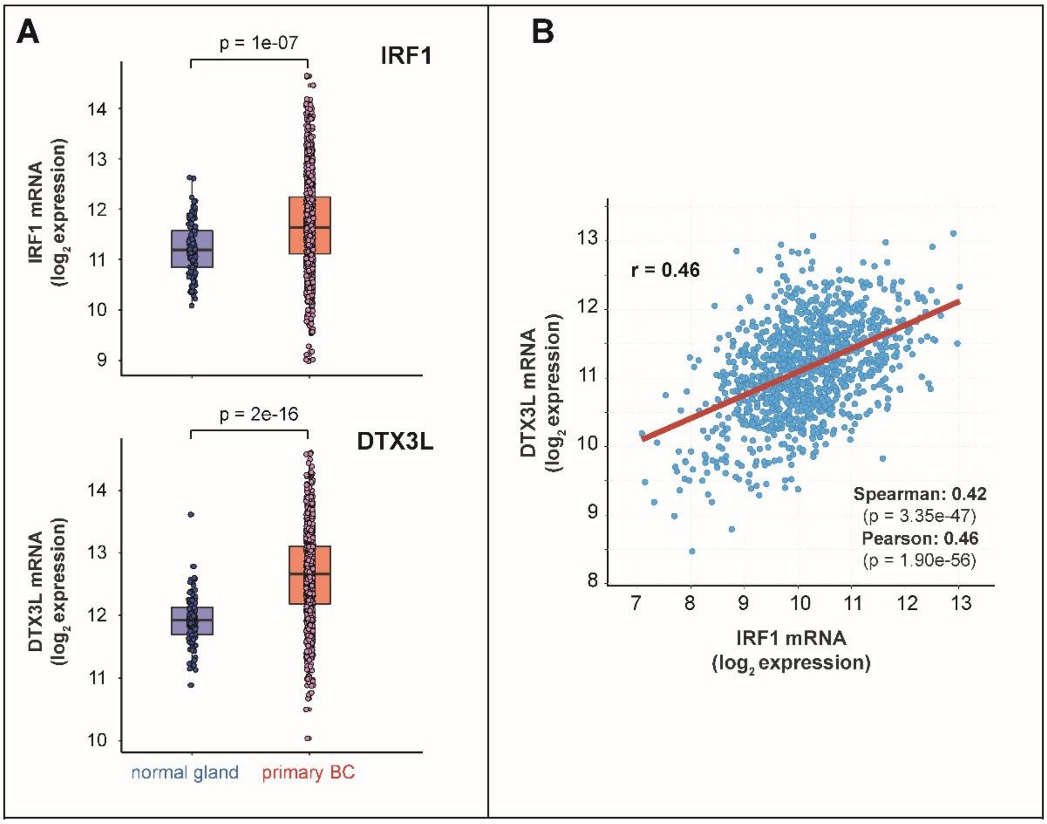

Supplementary Figure S15 *IRF1 and DTX3L mRNA expression in primary mammary tumors*
The levels of IRF1 and DTX3L mRNAs were determined on the basis of the *RNA-seq* data of the normal mammary glands and the primary breast-cancer samples available in the TCGA database. (**A**) The panel shows the mRNA expression levels of IRF1 and DTX3L in the single samples indicated. The p-values were adjusted for the FDR using DESEQ2 analysis pipeline. (**B**) The panel illustrates the correlations between the expression levels of DTX3L and IRF1 mRNAs in breast tumors and the normal mammary gland. The data demonstrate a significant direct correlation between the levels of the two mRNAs.

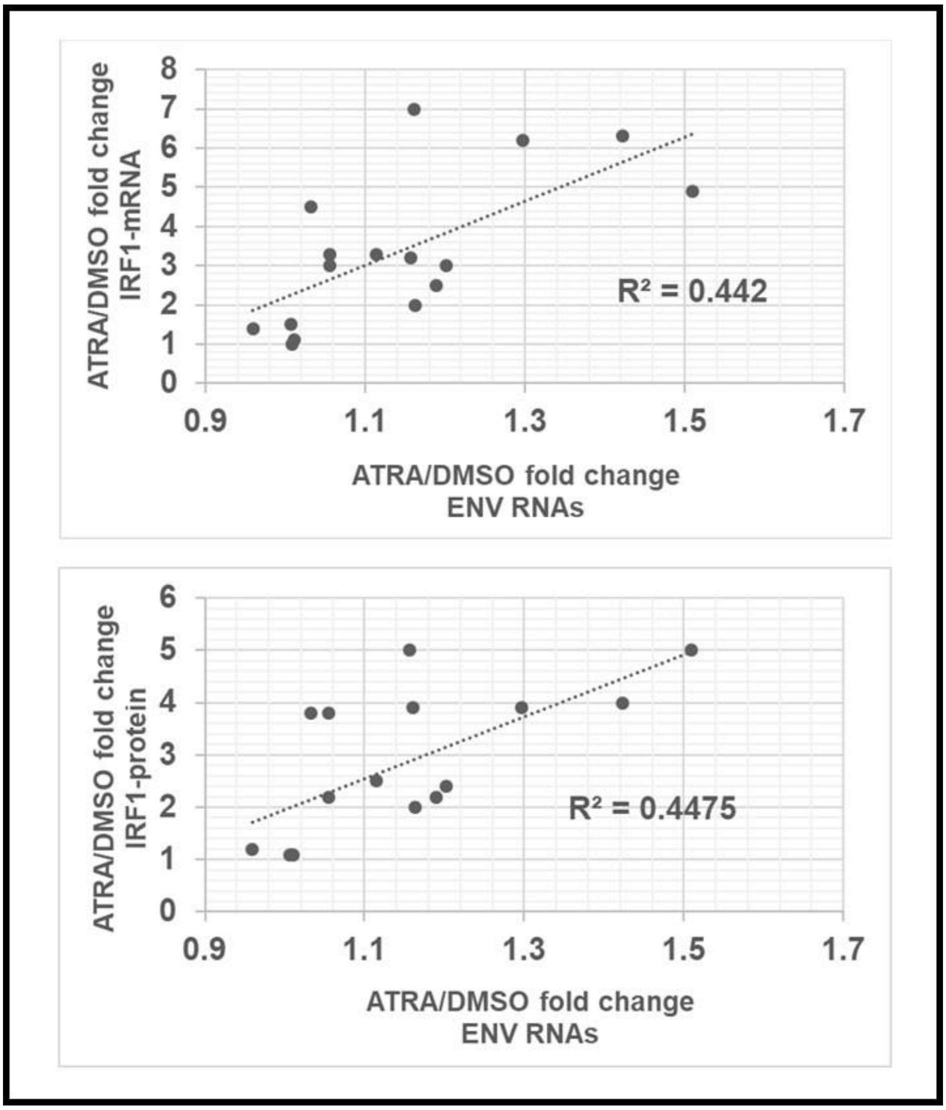

Supplementary Figure S16 *Correlations between ATRA-dependent IRF1 mRNA/protein and endogenous retrovirus induction in breast cancer cell-lines*

The upper diagram illustrates the correlation between the ATRA-dependent induction of the IRF1-mRNA shown in and the corresponding retinoid-dependent induction of ENV (Endogenous Retroviruses) RNAs (see Figures 5B and 7A). The lower diagram illustrates the same type of correlation observed between the ATRA-dependent induction of the IRF1-protein and the corresponding retinoid-dependent induction of ENV (Endogenous Retroviruses) RNAs.

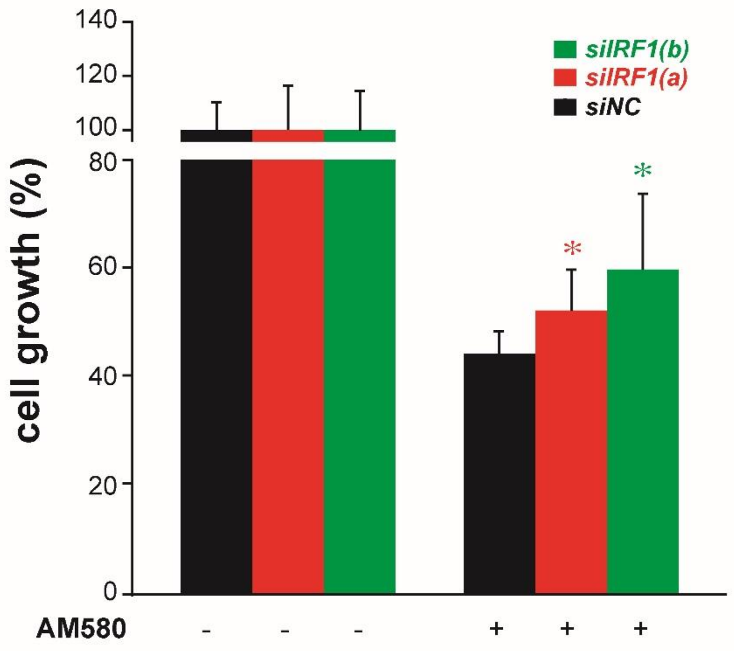

Supplementary Fig. S17 *Effects of IRF1 silencing on AM580-induced growth inhibition in SK-BR-3 cells*
SK-BR-3 cells were transiently transfected with *siIRF1(a)* and *siIRF1(b),* two siRNAs targeting IRF1, or the scrambled *siNC* control. The indicated cells were treated with the RARα agonist, AM580 (0.1 μM), for 96 hours. Cell growth was evaluated and data were analysed as in (**A**). Values are the mean $\pm$ SD of 3 replicate cultures.

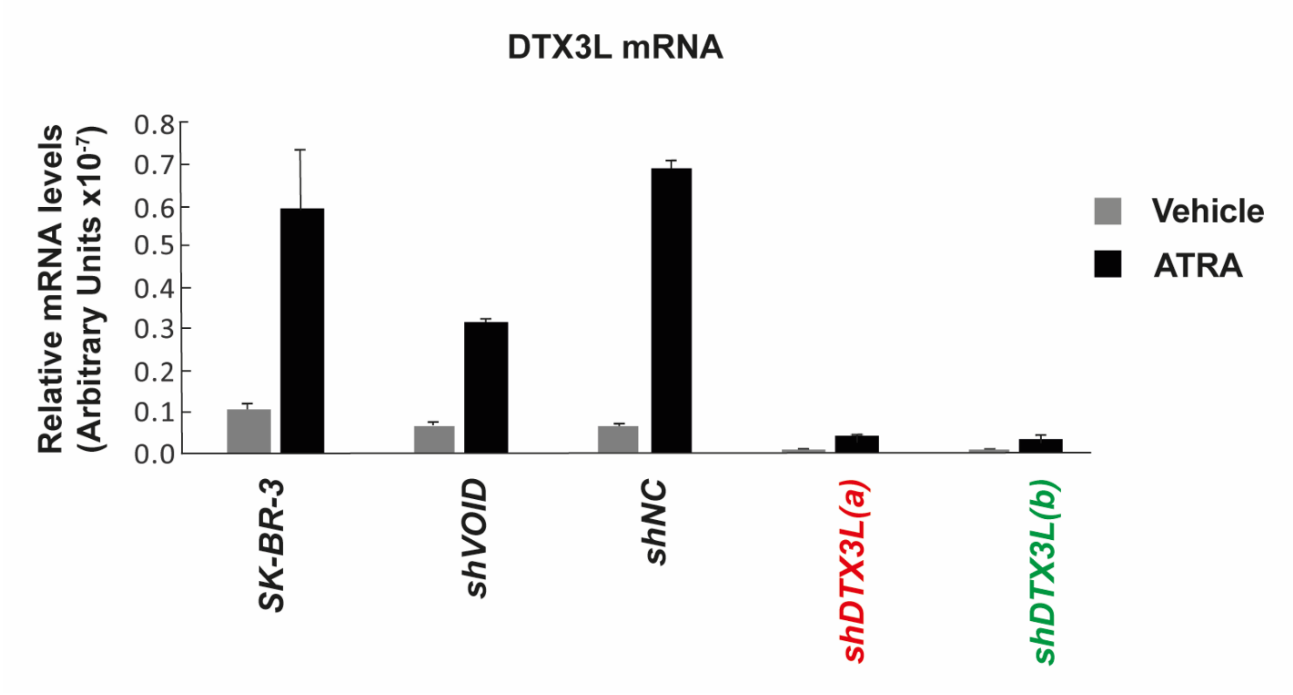

Supplementary Fig. S18 *Effects of DTX3L knock-down on DTX3L mRNA in SK-BR-3 cells*
SK-BR-3 cells were stably infected with *shDTX3L(a)*, *shDTX3L(b)*, the void lentiviral vector (*shVOID*) and a scrambled shRNA (*shNC*). Following antibiotic selection and isolation, the indicated cell populations were treated (24 hours) with vehicle (DMSO) or ATRA (1 µM). Total RNA was extracted and subjected to RT-PCR (Reverse Transcriptase-Polymerase Chain Reaction) analysis for *DTX3L* and 18S RNA using specific Taqman assays. The results of *DTX3L* mRNA expression are normalized for the expression of 18S RNA, which was used as an internal standard for the assay.

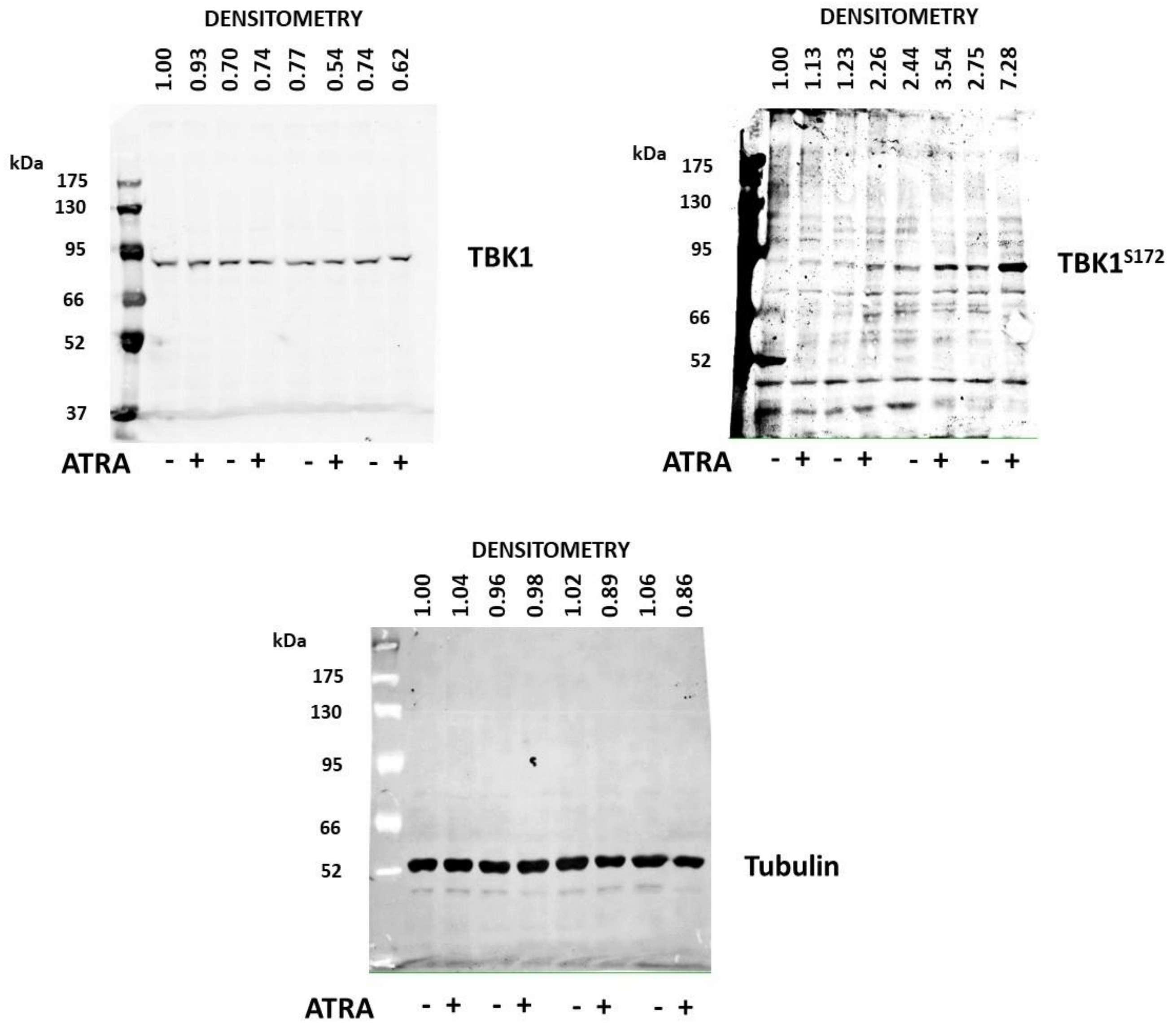

Legend: Original Western blots contained in **Figure 5D**

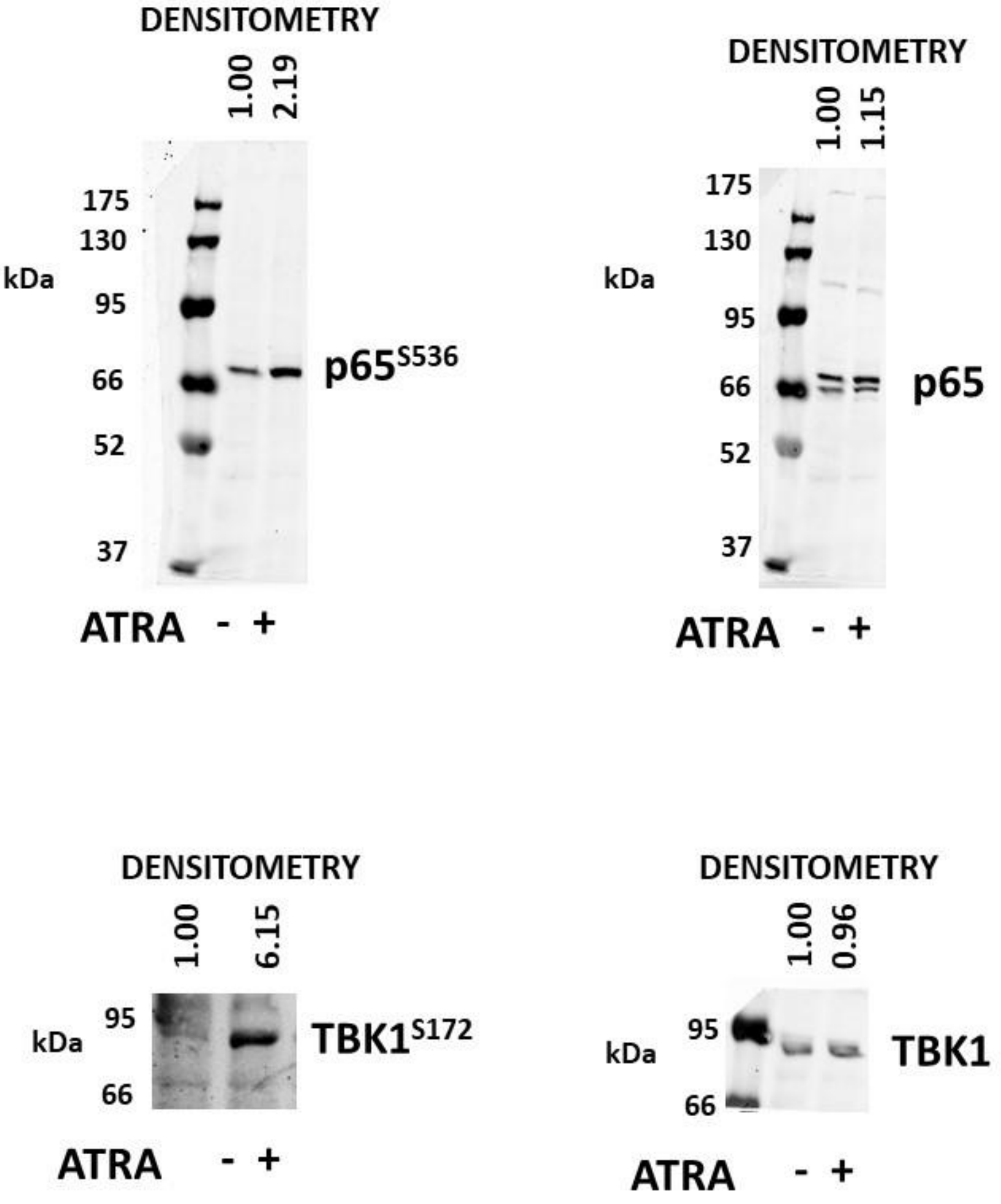

Legend: Original Western blots contained in **Figure 5D**

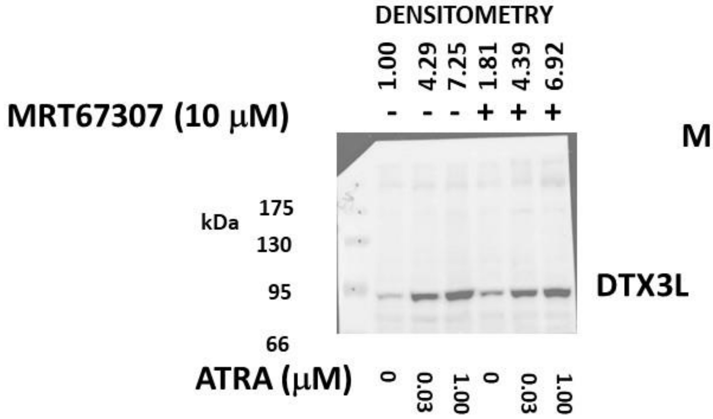

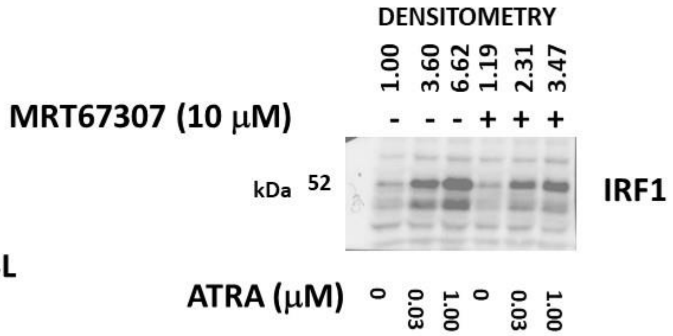

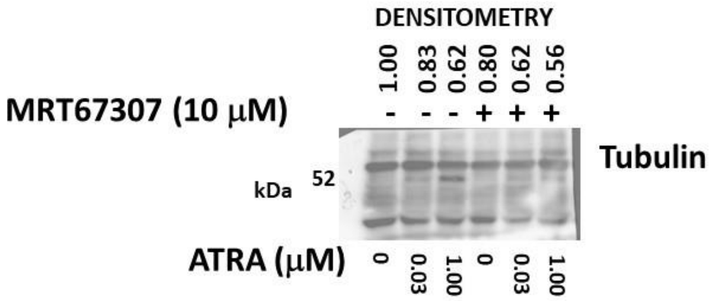

Legend: Original Western blots contained in **Figure 5E**

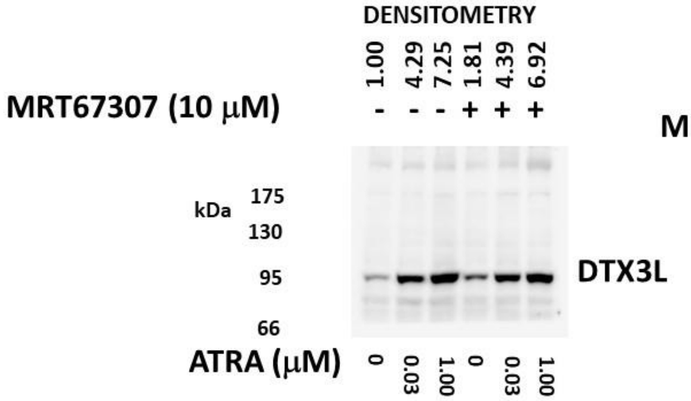

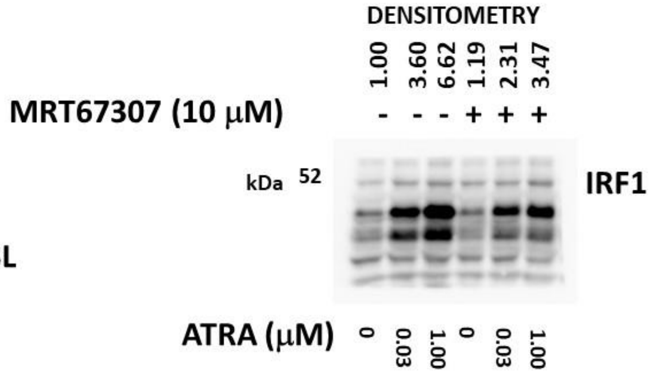

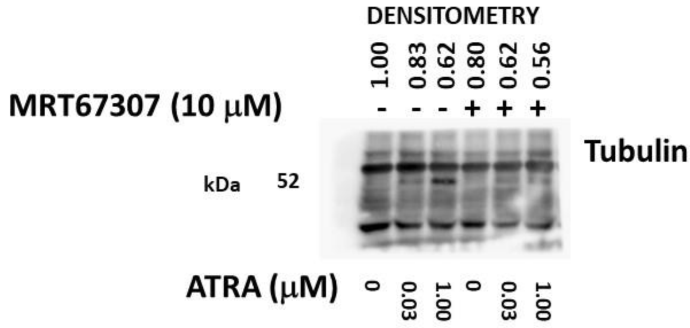

Legend: Original Western blots contained in **Figure 5E**

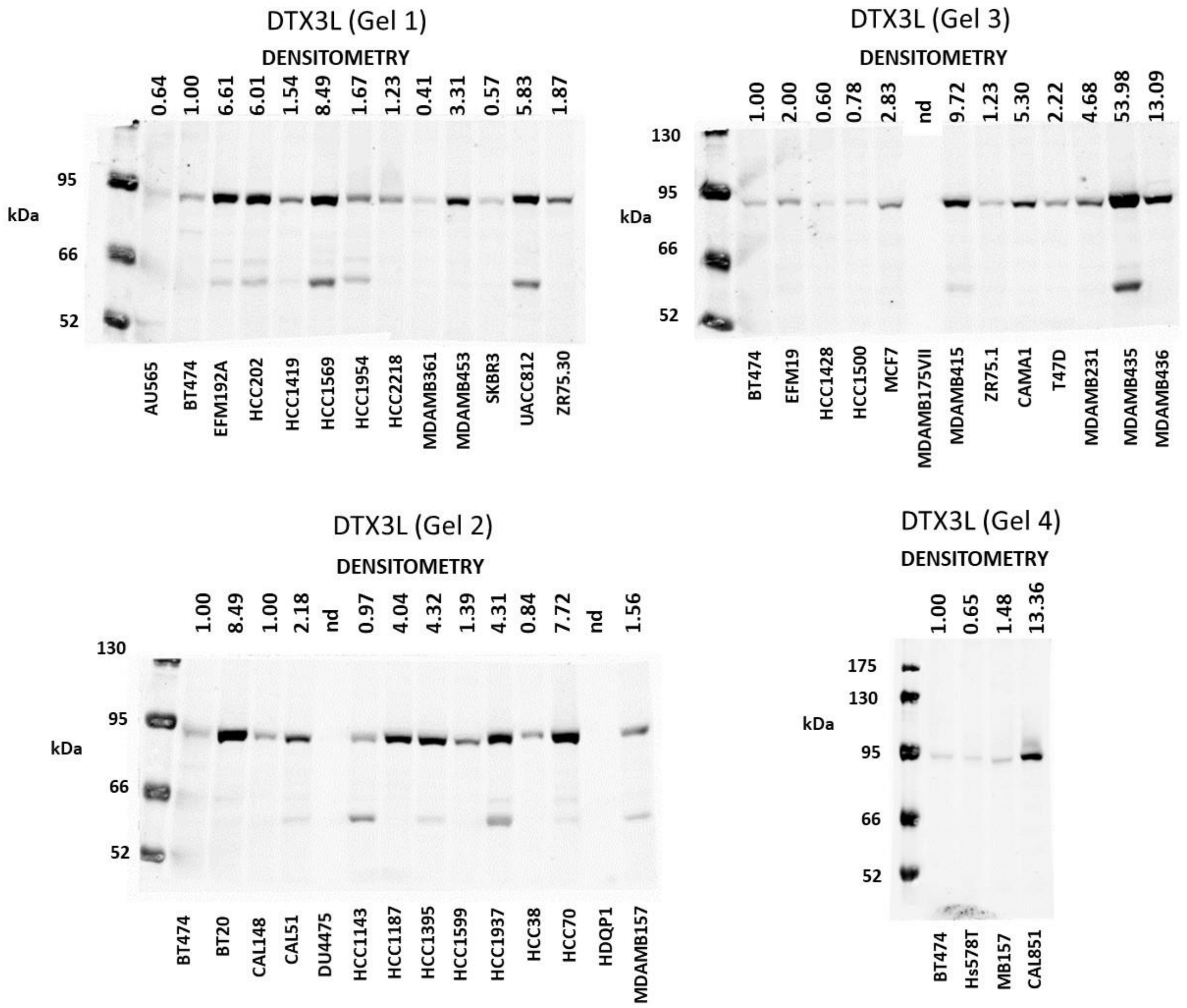

Legend: Original Western blots contained in **Figure 6**

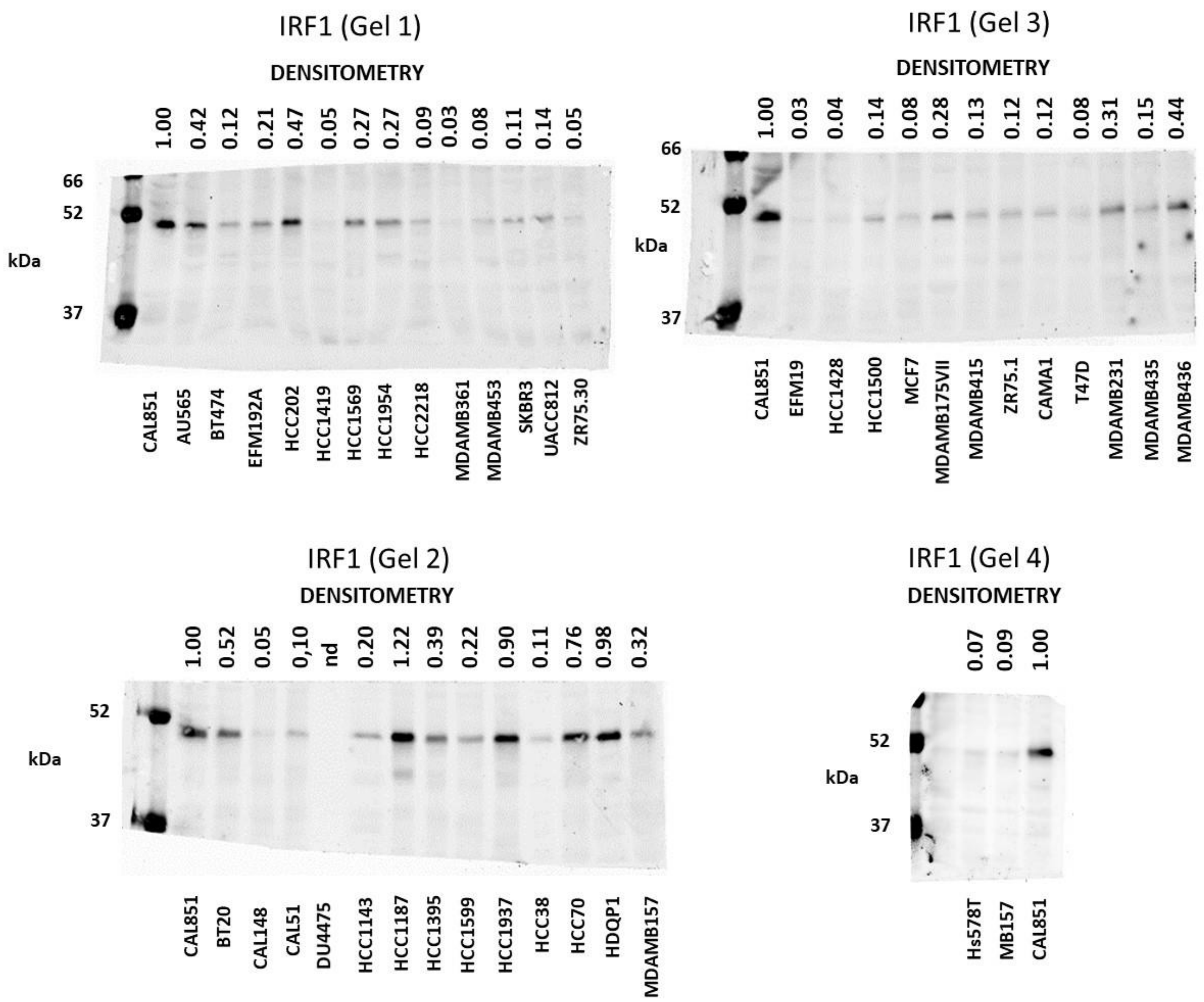

Legend: Original Western blots contained in **Figure 6**

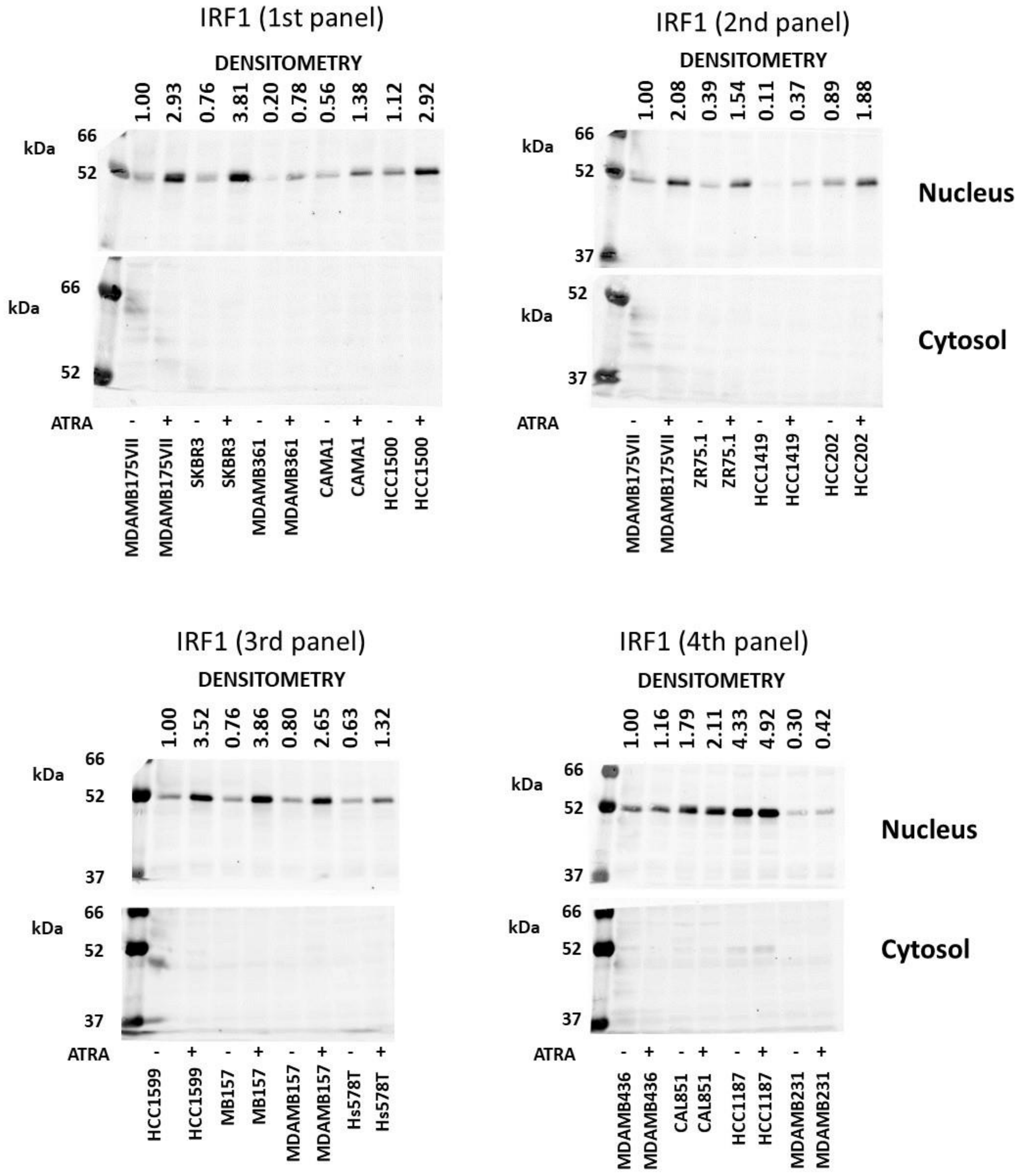

Legend: Original Western blots contained in **Figure 7A**

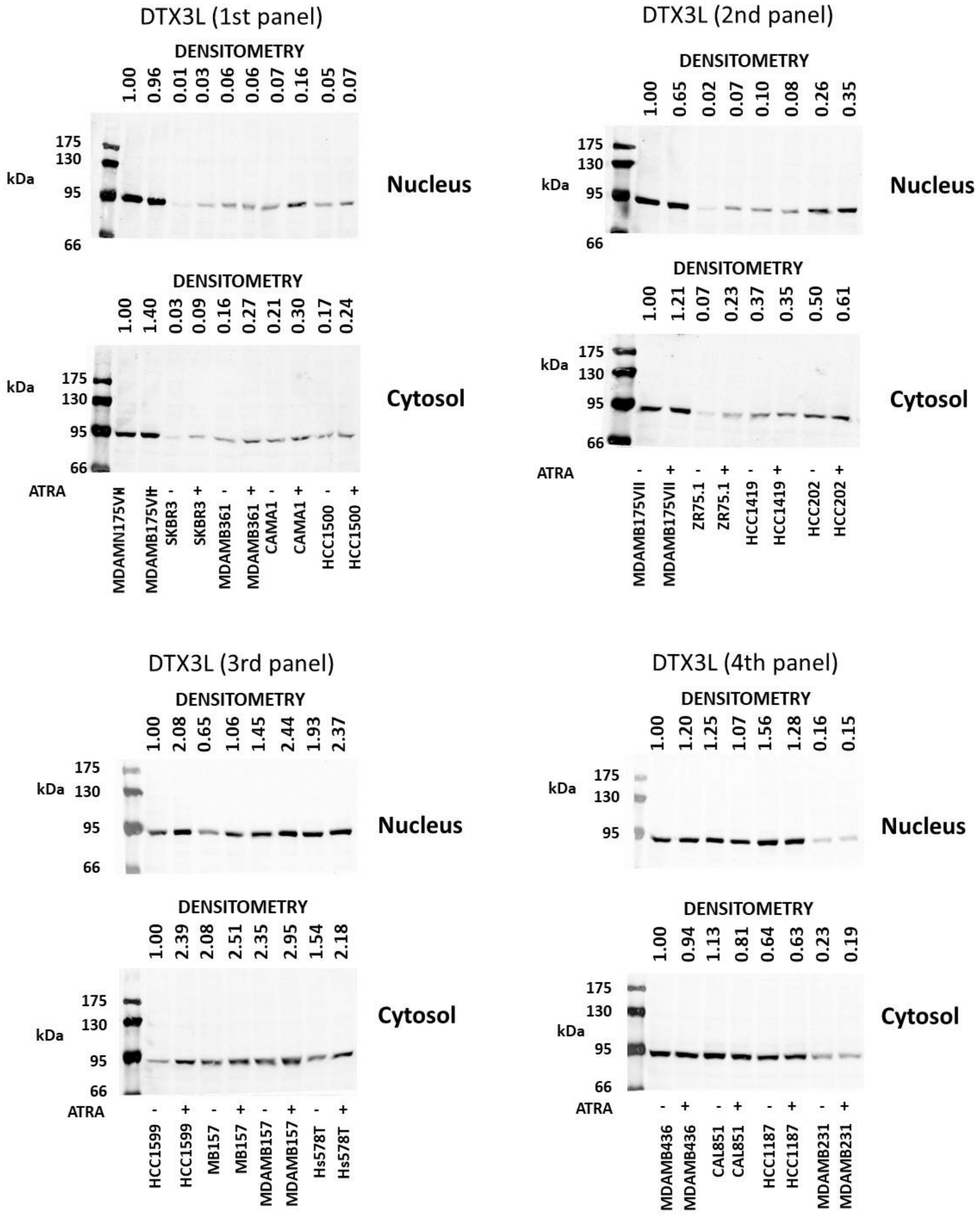

Legend: Original Western blots contained in **Figure 7B**

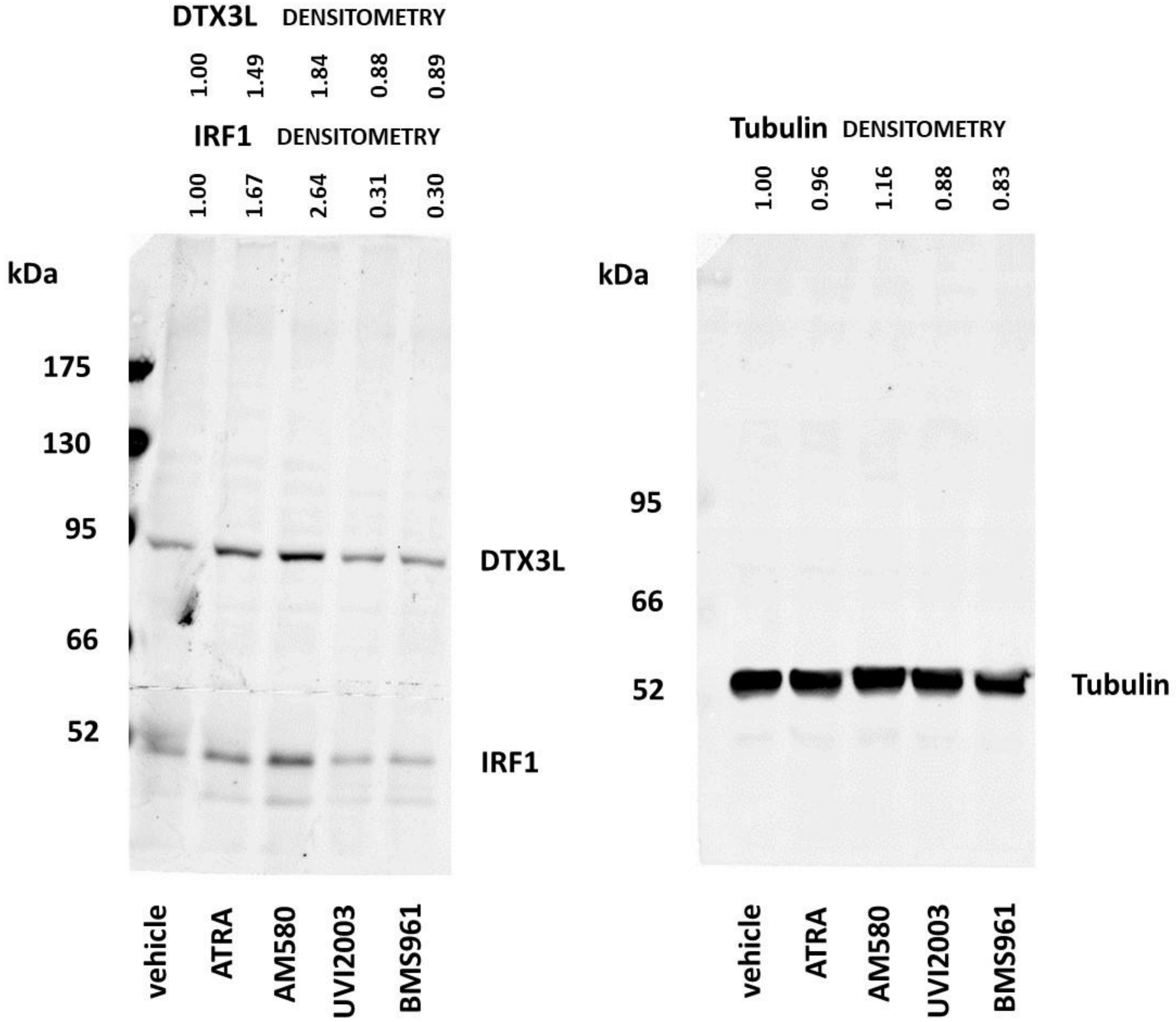

Legend: Original Western blots contained in **Figure 7C**

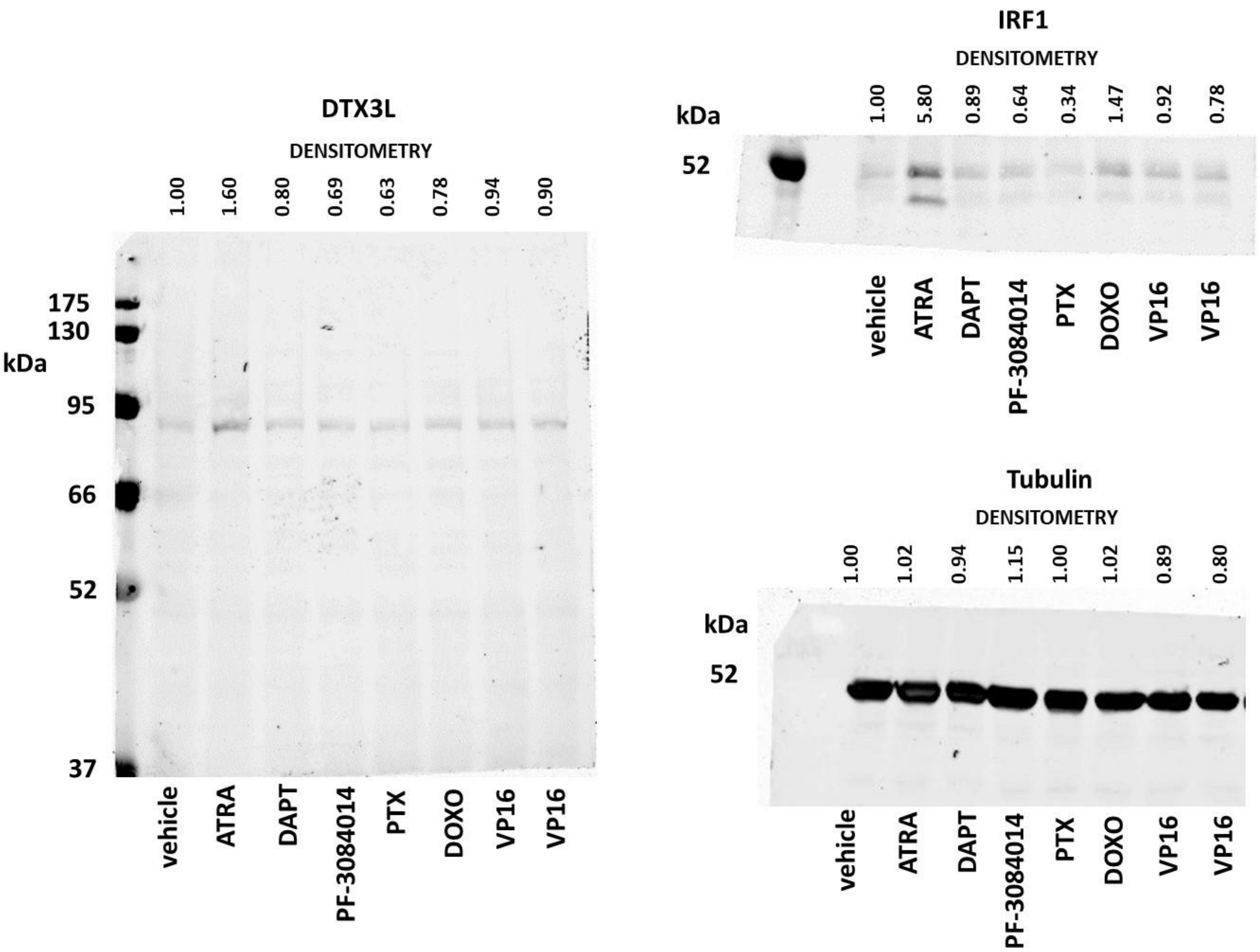

Legend: Original Western blots contained in **Figure 7D**

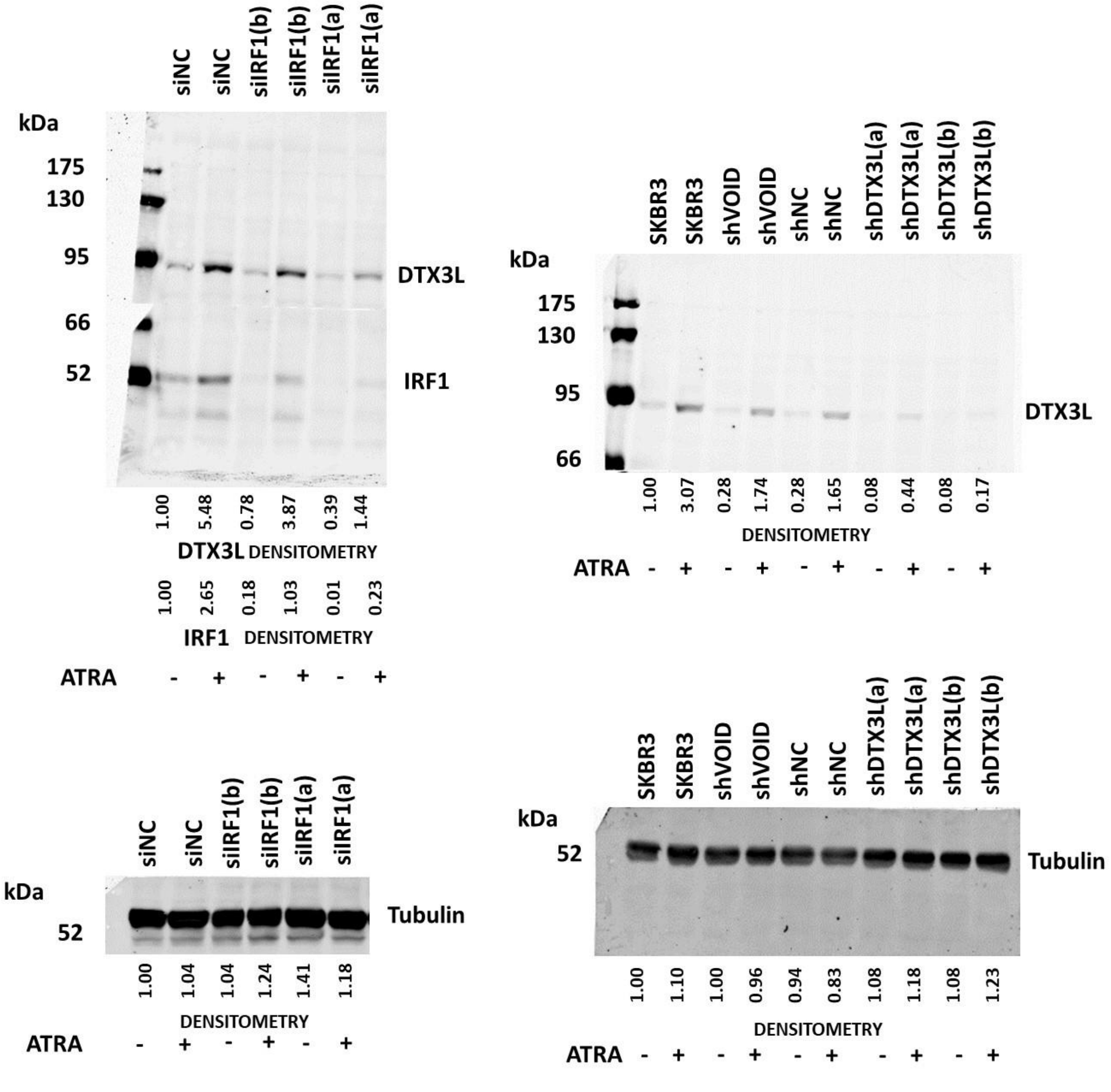

Legend: Original Western blots contained in **Figures 7E** and **7F**
