# Supplementary material for: All-Trans Retinoic Acid Stimulates Viral Mimicry, Interferon Responses and Antigen Presentation in Breast-Cancer Cells
Source: Cancers (Basel). 2020 May 6;12(5):1169. doi: 10.3390/cancers12051169 (PMC7281473; doi:10.3390/cancers12051169)
Supplement: Supplementary file 1 [file cancers-12-01169-s001.zip › cancers-776836 supplementary/Suppl. INFO_Cancers.pdf]

SUPPLEMENTARY INFORMATION

Title: All-trans retinoic acid stimulates viral mimicry, interferon responses and antigen presentation in breast-cancer cells

Marco Bolis[1,2], Gabriela Paroni[1], Maddalena Fratelli[1], Arianna Vallerga[1], Adriana Zanetti[1], Mami Kurosaki[1], Silvio Ken Garattini[3], Maurizio Gianni'[1], Monica Lupi[4], Linda Pattini[5], Maria Monica Barzago[1], Mineko Terao[1] and Enrico Garattini[1]

[1]Laboratory of Molecular Biology, Istituto di Ricerche Farmacologiche Mario Negri IRCCS, via La Masa 19, 20156 Milano Italy. [2]Functional Cancer Genomics Laboratory, Institute of Oncology Research; Bioinformatics Core Unit, SIB-Swiss Institute of Bioinformatics; USI, University of Southern Switzerland. Bellinzona, Switzerland. [3]DAME, Dipartimento di Area Medica Università degli Studi di Udine. Department of Oncology, Azienda Ospedaliera di Udine, Udine, Italy. [4] Department of Oncology, Istituto di Ricerche Farmacologiche Mario Negri IRCCS, via La Masa 19, 20156 Milano Italy. [5] Department of Electronics, Information and Bioengineering, Politecnico di Milano, Milano

Supplementary Methods	pages 2-4
Legends to Supplementary Tables S1-S4	pages 4-5
Supplementary Figures S1-S15	pages 6-20
Original Western blots of Figures 5-7	pages 21-31

SUPPLEMENTARY METHODS

Cell lines: The table below summarizes the characteristics and the source of the cell lines used throughout the study:

**Cell name**	**Gender**	**Phenotype**	**Source**
AU565	F	Luminal ($HER2^+$)	ATCC
BT20	F	Basal (TNBC)	ATCC
BT474	F	Luminal (ER^+/$HER2^+$)	ATCC
BT483	F	Luminal (ER^+)	ATCC
BT549	F	Basal (TNBC)	ATCC
CAL-120	F	Basal (TNBC)	DSMZ
CAL-148	F	Basal (TNBC)	DSMZ
CAL-51	F	Basal (TNBC)	DSMZ
CAL-851	F	Basal (TNBC)	DSMZ
CAMA1	F	Luminal (ER^+)	ATCC
DU4475	F	Basal (TNBC)	ATCC
EFM192A	F	Luminal (ER^+/$HER2^+$)	DSMZ
EFM19	F	Luminal (ER^+/$HER2^+$)	DSMZ
HCC-1143	F	Basal (TNBC)	ATCC
HCC-1187	F	Basal (TNBC)	ATCC
HCC-1395	F	Basal (TNBC)	ATCC
HCC-1419	F	Luminal ($HER2^+$)	ATCC
HCC-1428	F	Luminal (ER^+)	ATCC
HCC1500	F	Luminal (ER^+)	ATCC
HCC-1569	F	Basal (TNBC)	ATCC
HCC-1599	F	Basal (TNBC)	ATCC
HCC-1806	F	Basal (TNBC)	ATCC
HCC-1937	F	Basal (TNBC)	ATCC
HCC-1954	F	Basal (TNBC)	ATCC
HCC-202	F	Luminal ($HER2^+$)	ATCC
HCC-2218	F	Luminal ($HER2^+$)	ATCC
HCC-38	F	Basal (TNBC)	ATCC
HCC-70	F	Basal (TNBC)	ATCC
HDQP1	F	Basal (TNBC)	DSMZ
Hs281T	F	Basal (TNBC)	ATCC
Hs343T	F	Basal (TNBC)	ATCC
Hs578T	F	Basal (TNBC)	ATCC
KPL1	F	Luminal (ER^+)	DSMZ
MB-157	F	Basal (TNBC)	ATCC
MCF7	F	Luminal (ER^+)	ATCC
MDA-MB-157	F	Basal (TNBC)	ATCC
MDA-MB-134VI	F	Luminal (ER^+)	ATCC
MDA-MB-175VII	F	Luminal (ER^+)	ATCC

MDA-MB-231	F	Basal (TNBC)	ATCC
MDA-MB-361	F	Luminal (ER^+/$HER2^+$)	ATCC
MDA-MB-415	F	Luminal (ER^+)	ATCC
MDA-MB-435S	F	Basal (TNBC)	ATCC
MDA-MB-436	F	Basal (TNBC)	ATCC
MDA-MB-453	F	Basal (TNBC)	ATCC
MDA-MB-468	F	Basal (TNBC)	ATCC
SK-BR-3	F	Luminal ($HER2^+$)	ATCC
T47D	F	Luminal (ER^+)	ATCC
UACC-812	F	Luminal ($ER2^+$/$HER2^+$)	ATCC
ZR75.1	F	Luminal (ER^+)	ATCC
ZR75.30	F	Luminal (ER^+/$HER2^+$)	ATCC

ATCC = American Type Culture Collection; DSMZ = Deutsche Sammlung von Mikroorganismen und Zellkulturen; SIGMA = Sigma-Aldrich. The cell-lines marked in yellow are the 16 cell-lines used to perform the RNA-seq experiments following treatment with ATRA.

Polymerase Chain Reaction experiments: The expression of the following transcripts was evaluated with the use of commercially available taqman assays according to the instructions of the manufacturer (Applied Biosystems): Dihydroorotate dehydrogenase (DHODH, NM_001361.4; assay ID-Hs00361406_m1); Minichromosome maintenance complex component 6 (MCM6, NM_005915.5; assay ID-Hs00195504_m1); Minichromosome maintenance complex component 2 (MCM2, NM_004526.3; assay ID-Hs01091568_g1); Poly (ADP-ribose) polymerase family, member 14 (PARP14, NM_017554.2; Hs00393814_m1).

Identification of genetic perturbations proportional to ATRA-sensitivity and generation of ATRA-sensitivity associated gene interaction networks: After a first phase of differential expression analysis, we determined for each gene, the correlation between ATRA-induced fold changes across cell-lines and the respective ATRA sensitivity scores. Subsequently, we filtered out genes with either Pearson's or Spearman's correlation coefficients associated p-values lower than 0.01. We adopted both metrics in order to obtain more inclusive results. We further discarded genes showing a

coefficient of variation across cell-lines being lower than 0.5 and obtained a final list consisting of 754 genes, whose perturbations are associated with ATRA responsiveness. Finally, we constructed a protein-protein interaction network using the *STRING* database and discarded all elements with less than three edges. A scheme of the strategy used for the analysis of the data is available in Supplementary Figure S2.

LEGENDS TO SUPPLEMENTARY TABLES

Supplementary Table S1 *Genes up- and down-regulated by ATRA in breast-cancer cell-lines*

The indicated cell-lines were treated with DMSO or ATRA (1 μM) for 24 hours. Whole-genome gene-expression analysis was performed by *RNA-seq*. The table contains the list of 754 genes significantly up- and down-regulated by ATRA and whose ATRA-dependent expression is quantitatively and significantly correlated with the *ATRA-score*. The expression data are shown as the $\log_2$ of the ATRA/DMSO fold-change and the values calculated derive from 3 biological replicates. The values corresponding to the following correlation indexes are provided: pRHO = p-value of the Spearman's correlation between ATRA/DMSO fold-change and *ATRA-score*; RHO = Spearman's correlation coefficient between ATRA/DMSO fold-change and *ATRA-score*; pR = p-value of the Pearson's correlation between ATRA/DMSO fold-change and *ATRA-score*; R = Pearson's correlation coefficient between ATRA/DMSO fold-change and *ATRA-score*; CV = Coefficient of variation.

Supplementary Table S2 *Enrichment analyses of the gene-sets up- and down-regulated by ATRA which are quantitatively associated with ATRA sensitivity in breast-cancer cell-lines*

Each breast-cancer cell-line was treated with vehicle (DMSO) or ATRA (10^{-6} M) for 24 hours. Total RNA was extracted and subjected to *RNA-seq* analysis. The gene-sets up- or down-regulated

by ATRA and whose significance is proportional to ATRA-sensitivity were subjected to GSEA (Gene Set Enrichment Analysis) using the HALLMARK and the CURATED datasets. The FDR (False Discovery Rate) values and the ATRA-dependent up-or down-regulation are indicated.

Supplementary Table S3 *Enrichment analyses of the gene-sets up- and down-regulated by ATRA in HCC-1599 xenografts*

Three separate SCID mice/experimental group were transplanted sub-cutaneously with *HCC-1599* cells. Animals were administered two daily doses of vehicle or ATRA (15 mg/kg) orally. RNA was extracted from the isolated tumor tissues and subjected to microarray gene-expression analysis. The gene-sets up- or down-regulated by ATRA were subjected to GSEA (Gene Set Enrichment Analysis) using the HALLMARK and the CURATED datasets. The FDR values and the ATRA-dependent up-or down-regulation are indicated.

Supplementary Table S4 *Effect of ATRA on the expression of interferon genes in breast cancer cell-lines*

Each breast-cancer cell-line was treated with vehicle (DMSO) or ATRA (10^{-6} M) for 24 hours. Total RNA was extracted and subjected to *RNA-seq* analysis. The expression levels of the transcripts encoding the indicated interferon (IFN) species were determined. Each value is the mean of 3 replicate cultures treated with vehicle (DMSO) or ATRA.

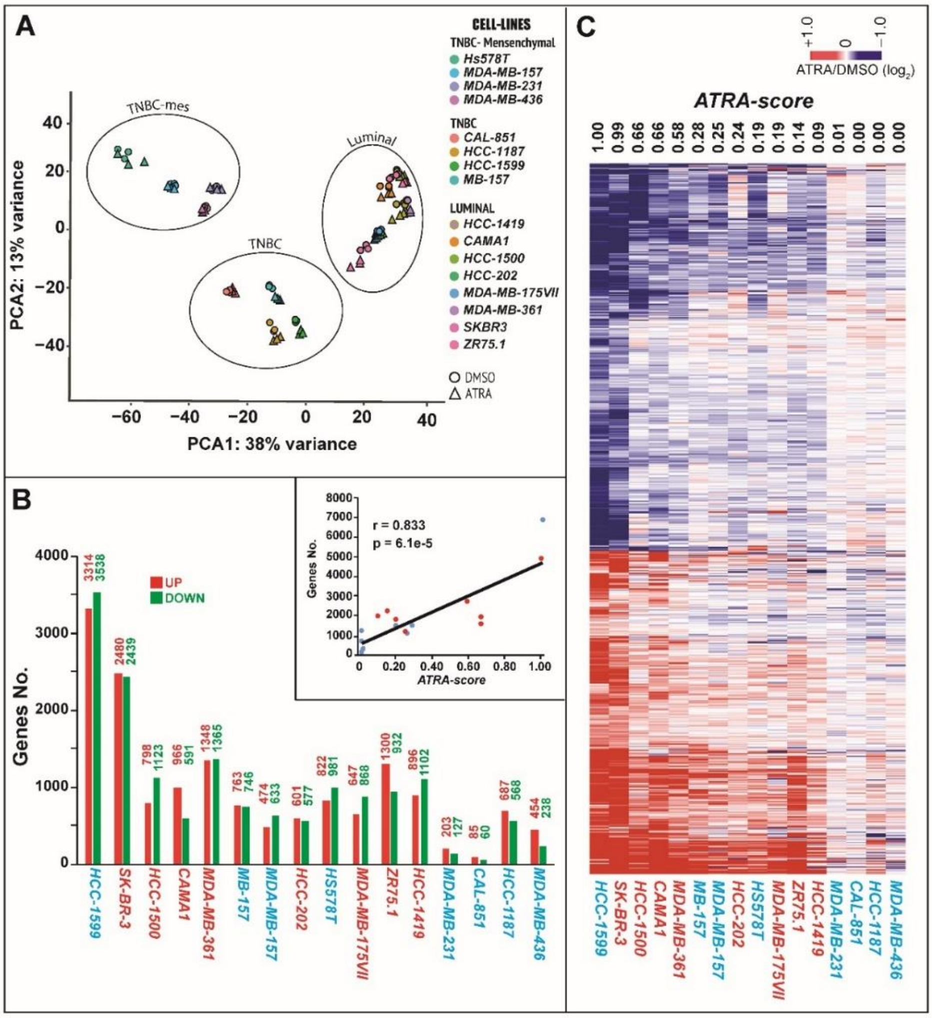

Supplementary Figure S1 *Gene expression profiles of the breast-cancer cell-lines following exposure to ATRA*

Three replicate cultures of the indicated and logarithmically growing cell-lines were treated with vehicle (DMSO) or ATRA (1 μM) for 24 hours. RNA was extracted and subjected to *RNA-seq* analysis. (**A**) The diagram illustrates a bi-dimensional principal component analysis (PCA) of the *RNA-seq* data obtained in each cell-line exposed to DMSO or ATRA. The gene-expression data separate the cell-lines in three distinct groups, *TNBC*, *TNBC-mes* and *Luminal*, which recapitulate their phenotypic and morphological characteristics. ATRA-treatment does not cause transitions across the 3 groups of cell-lines. *TNBC* = triple-negative breast-cancer; *TNBC-mes* = triple-negative breast-cancer with a mesenchymal phenotype. (**B**) The column graph indicates the number of genes selectively up- (red) or down-regulated (green) by ATRA in each cell-line. The cell-lines are ranked from left to right according to their decreasing sensitivity to ATRA (decreasing *ATRA-score* value). Luminal cell-lines are indicated in red, while basal cell-lines are marked in blue. The inset shows the linear correlation between the *ATRA-score* values and the total number of up- and down-regulated genes determined in each cell-line. The calculated r value for the correlation is shown. (**C**) The heat-map shows the 754 genes whose up-or down-regulation is proportional to ATRA-sensitivity. The cell-lines are ranked from left to right according to their decreasing sensitivity to ATRA. The *ATRA-score* values are shown above the heat-map. Luminal cell-lines are indicated in red, while basal cell-lines are marked in blue.

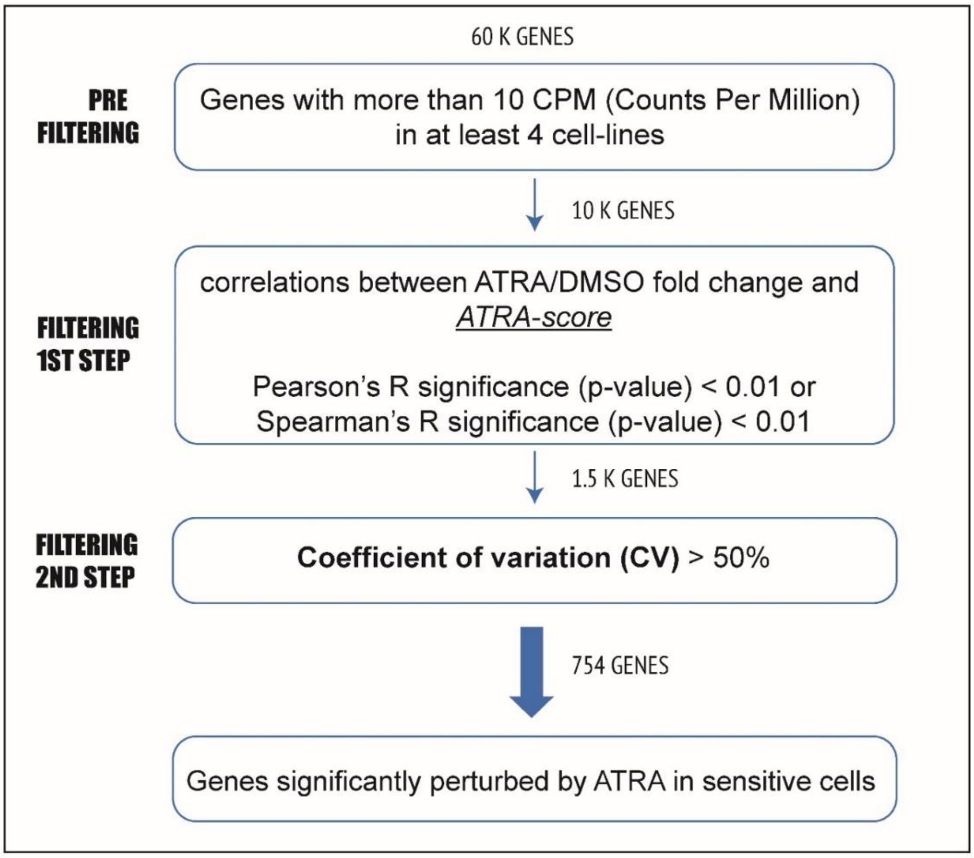

Supplementary Figure S2 *Identification of the genes differentially regulated by ATRA in sensitive cell-lines*

Biological triplicates of 16 breast cancer cell-lines were treated with vehicle (DMSO) or ATRA (10^{-6} M) for 24 hours. Total RNA was extracted and subjected to *RNA-seq* analysis. The flow-chart illustrates the computational approach applied to define the 754 genes whose expression is significantly perturbed by ATRA and quantitatively correlated with cell-line sensitivity to the anti-proliferative activity of ATRA (*ATRA-score* value).

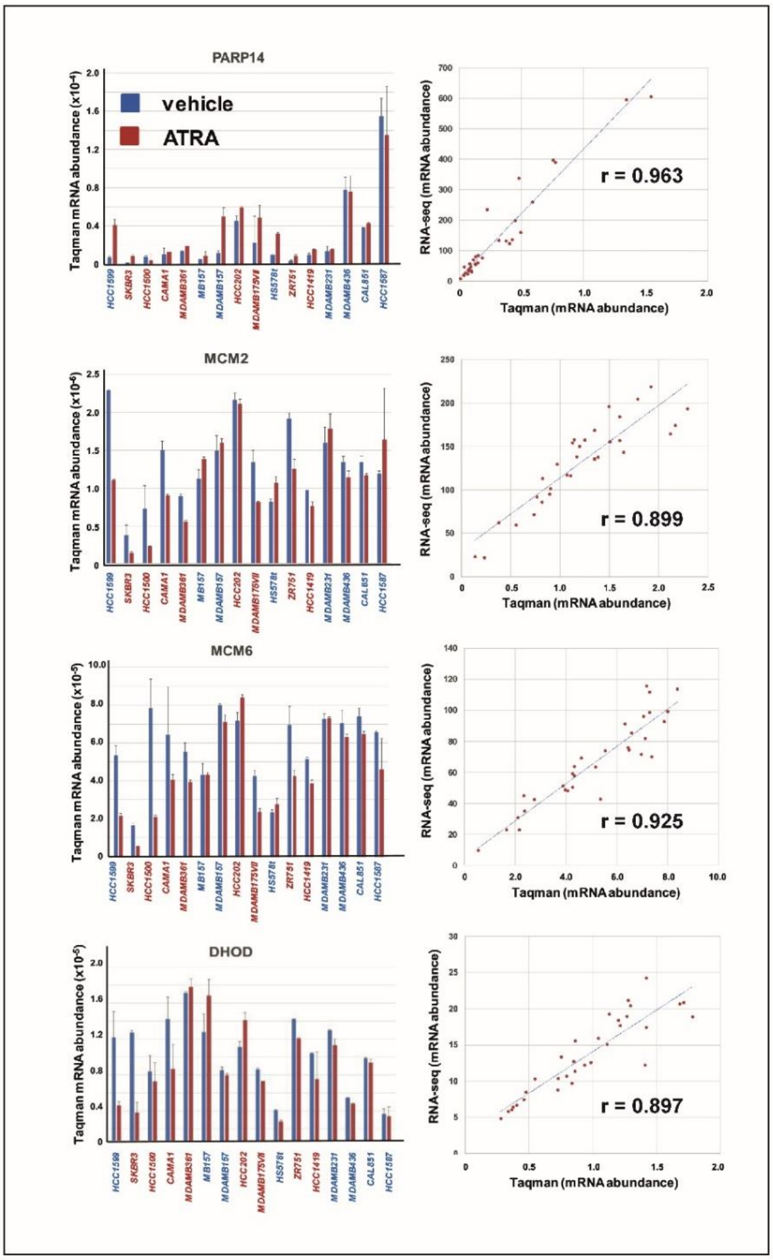

Supplementary Figure S3 *Validation of the RNA-seq data*

The *RNA-seq* data were validated by Taqman analysis of the 4 indicated genes in all the cell-lines considered. The indicated cell-lines were treated with vehicle (DMSO) or ATRA (10^{-6} M) for 24 hours. Total RNA was extracted and subjected to Taqman analysis using specific oligonucleotide probes targeting the PARP14, MCM2, MCM6 and DHODH mRNAs. Each column represents the Mean $\pm$ SD of 3 replicate cultures. The scatter plots on the right show the correlation between the Taqman and the *RNA-seq* data in each cell-line exposed to vehicle or ATRA (n = 32). The r value of each correlation is shown. PARP14 = Poly (ADP-ribose) polymerase family, member 14; MCM2 = Minichromosome maintenance complex component 2; MCM6 = Minichromosome maintenance complex component 6; DHODH = dihydroorotate dehydrogenase.

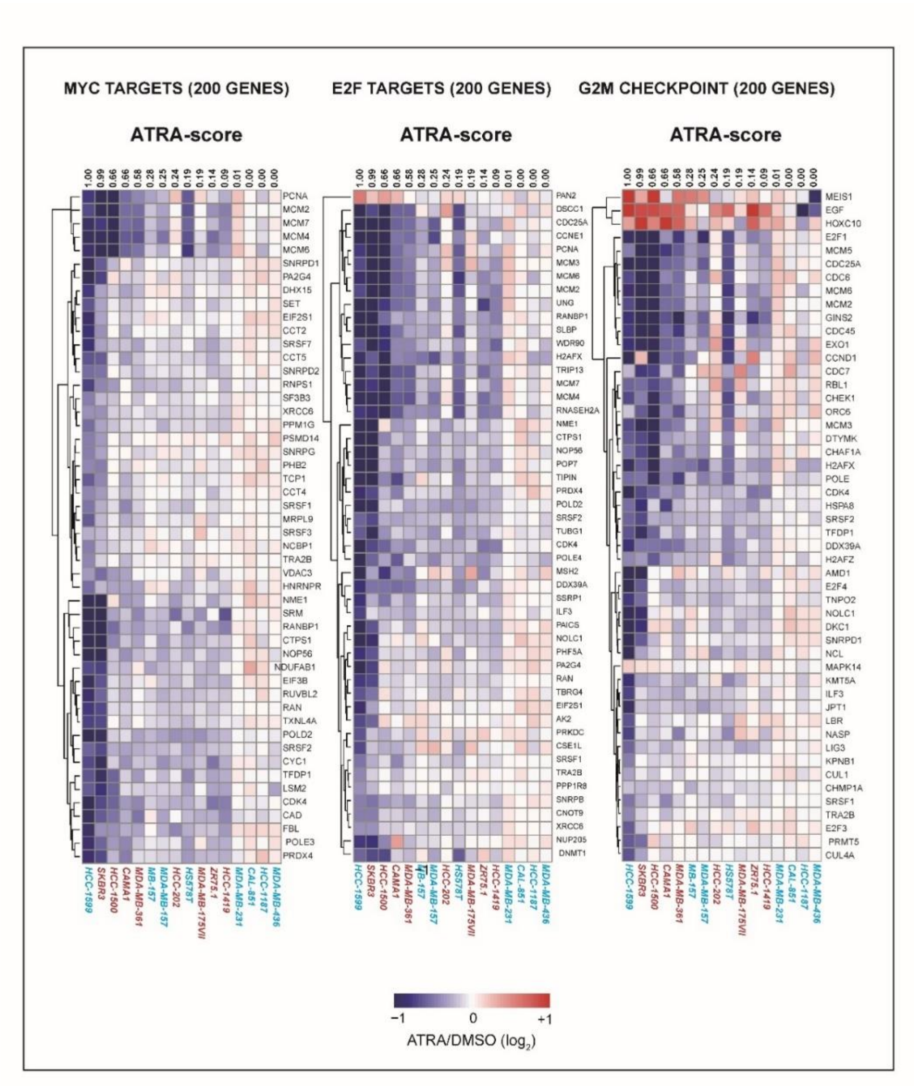

Supplementary Figure S4 *Effects of ATRA on the genes belonging to the "MYC Targets", "E2F Targets" and "G2M Checkpoint" gene-sets*

The indicated cell lines were treated with vehicle (DMSO) or ATRA (1 μM) for 24 hours. Total RNA was extracted and subjected to *RNA-seq* analysis. The heat-maps show the effects exerted by ATRA on the expression of the genes belonging to the CURATED "*MYC Targets*", *"E2F Targets"* and *"G2M Checkpoint"* gene-sets. The data are expressed as the ATRA/DMSO fold change. The "*MYC Targets*" gene-set is a merged version of the CURATED "*MYC Targets V1*" and the "*MYC Targets V2*" gene-sets. Each of the "*MYC Targets*" *"E2F Targets"* and *"G2M Checkpoint"*gene-sets consist of 200 genes and the heat-maps show the 50 top-ranking genes significantly correlated to the *ATRA-score* of the indicated cell-lines. The cell-lines are ordered according to their *ATRA-score* from left to right and the *ATRA-score* values are shown on the top. The cell-lines marked in blue have a basal phenotype, while the cell-lines marked in red are endowed with a luminal phenotype.

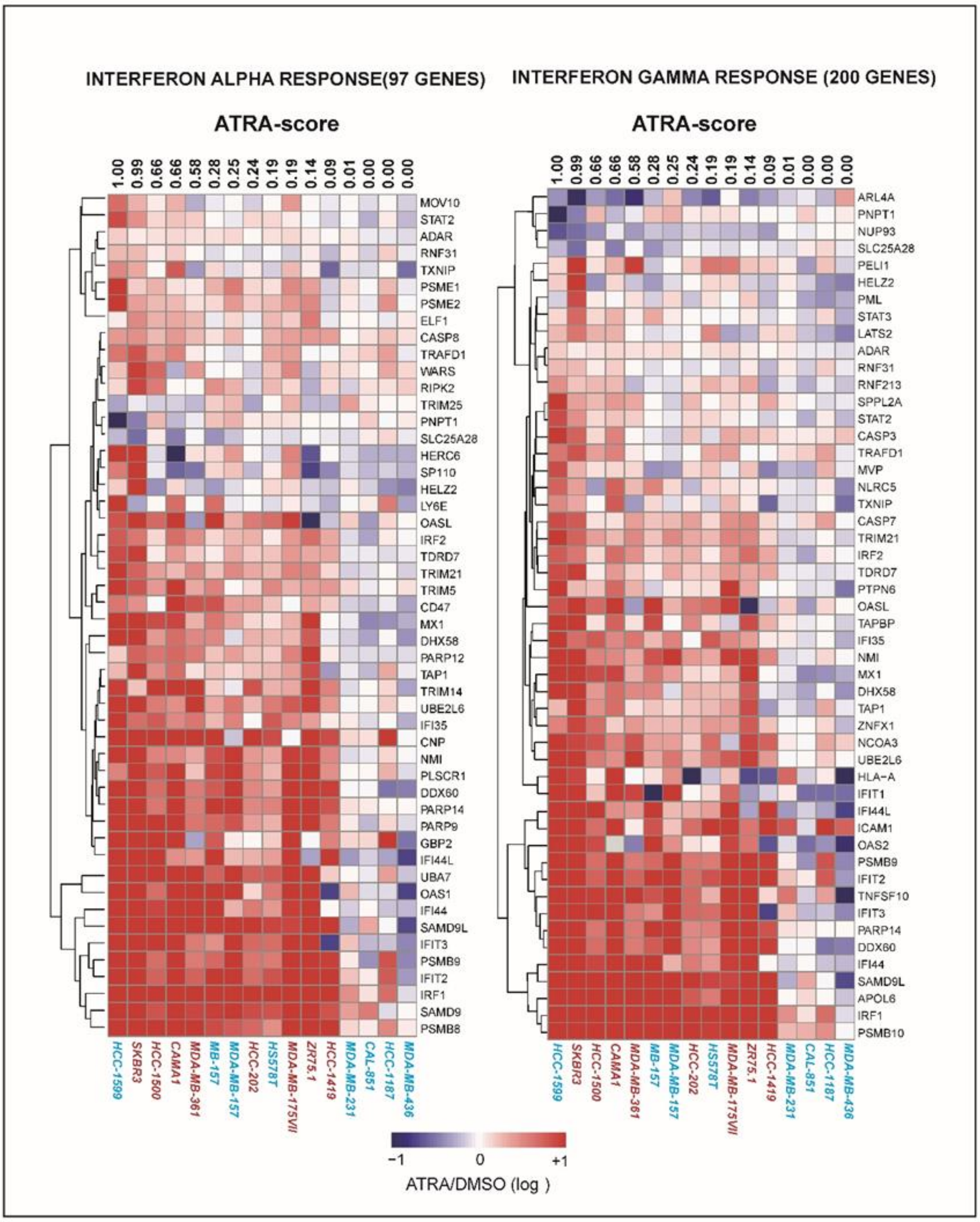

Supplementary Figure S5 *Effects of ATRA on the genes belonging to the "Interferon Alpha Response" and "Interferon Gamma Response" gene-sets*

The indicated cell lines were treated with vehicle (DMSO) or ATRA (1 μM) for 24 hours. Total RNA was extracted and subjected to *RNA-seq* analysis. The heat-maps show the effects exerted by ATRA on the expression of the genes belonging to the "*Interferon Alpha Response*" and *"Interferon Gamma Response"* CURATED gene-sets. The data are expressed as the ATRA/DMSO fold change. The "*Interferon Alpha Response*" and *"Interferon Gamma Response"* gene-sets consists of 97 and 200 genes, respectively, and the heat-maps show the 50 top-ranking genes significantly correlated to the *ATRA-score* of the indicated cell-lines. The cell-lines are ordered according to their *ATRA-score* from left to right and the *ATRA-score* values are shown on the top. The cell-lines marked in blue have a basal phenotype, while the cell lines marked in red are endowed with a luminal phenotype.

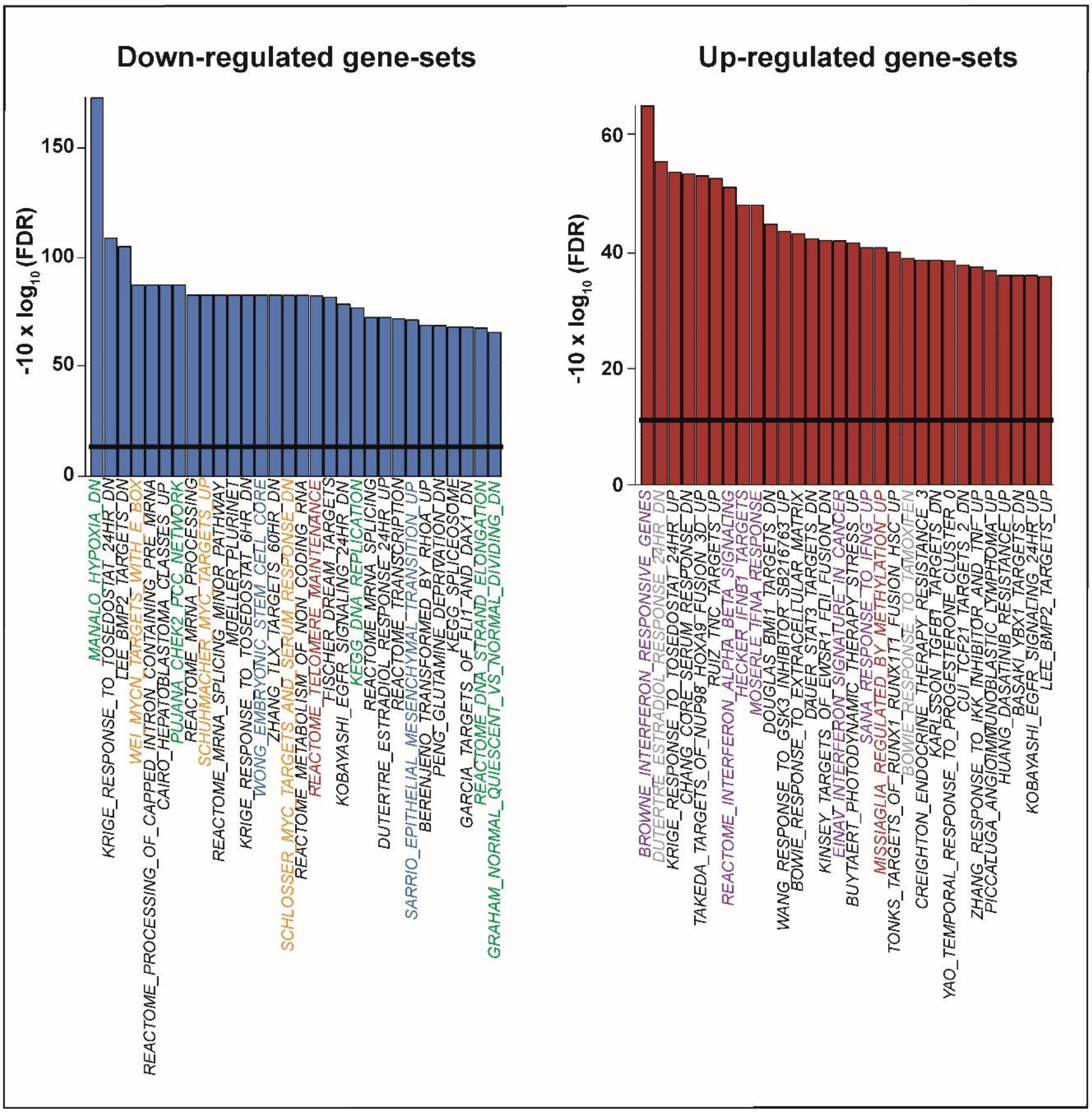

Supplementary Figure S6 *Gene-network enrichment analysis of the RNA-seq results obtained in breast-cancer cell-lines*

Gene-network enrichment analysis was performed on the 754 genes whose up-or down-regulation is proportional to ATRA-sensitivity, using the CURATED gene-sets. The top 30 down-regulated gene-networks are shown by the blue-column graph on the left, while the top 30 up-regulated gene-networks are illustrated by the red-column graph on the right. The horizontal line indicates the FDR threshold value considered. Gene-sets involved in in the control of: Green = proliferation/cell-cycle; Brown = histone/chromatin organization and *"Missiaglia-Regulated-by-Methylation-UP"*; Orange = Myc-pathway; Grey = estrogen-responses; Violet = interferon/inflammatory responses.

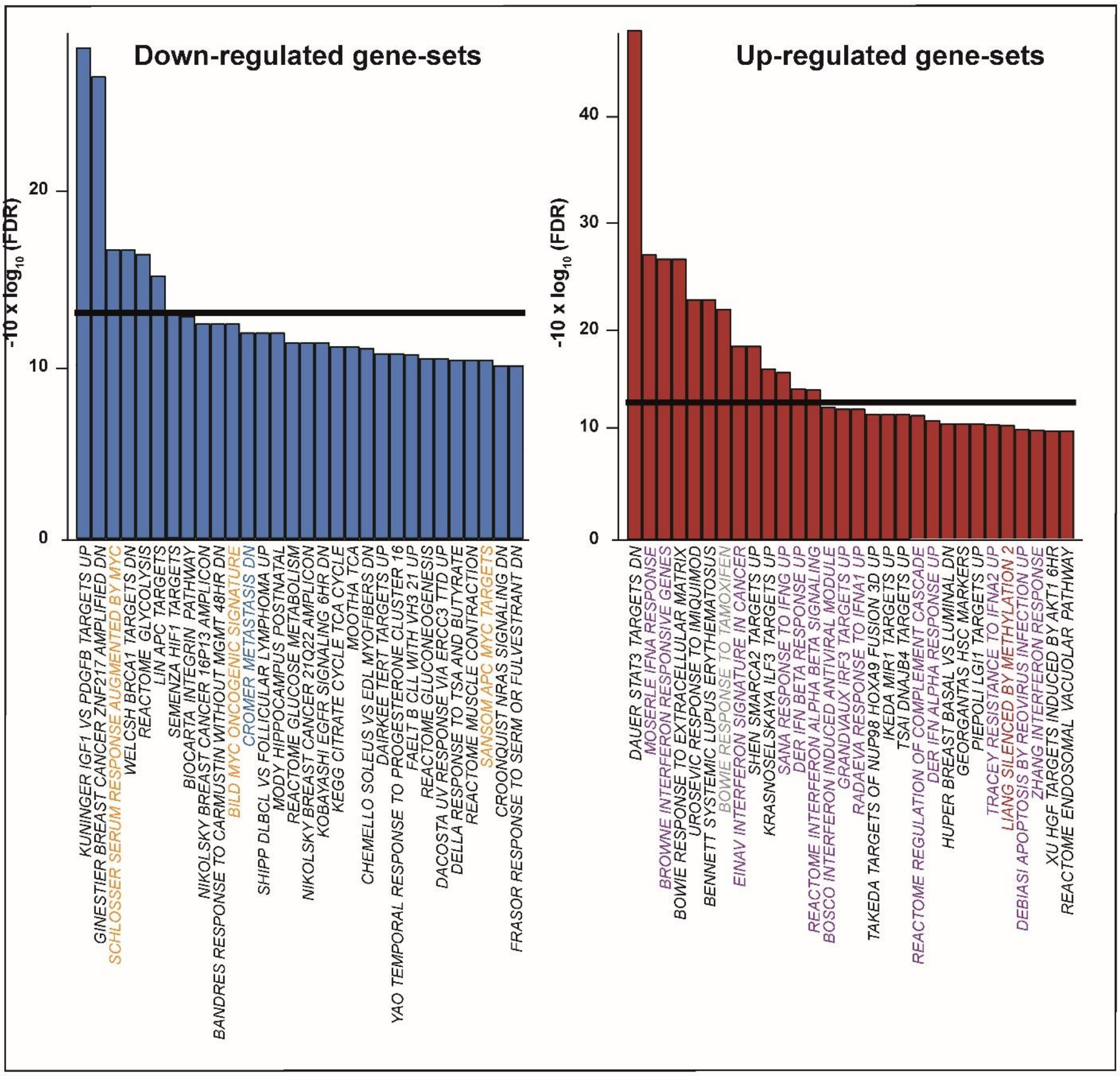

Supplementary Figure S7 *Gene-network enrichment analysis of the RNA-seq results obtained in HCC-1599 xenografts*

HCC-1599 cells were transplanted in nude mice subcutaneously. Xenografted animals were treated with vehicle or ATRA according to the scheme illustrated in Centritto *et al*. (EMBO Mol Med 7, 950–972). Twenty four hours following the last treatment, the *HCC-1599* xenografts of 3 ATRA-treated and 3 vehicle treated animals were subjected to whole-genome gene-expression microarray analysis. Genes significantly up- and down-regulated by ATRA were subjected to gene-network enrichment analysis using the CURATED gene-sets. The top 30 down-regulated gene-sets are shown by the blue-column graph on the left, while the top 30 up-regulated gene-sets are illustrated by the red-column graph on the right. The horizontal line indicates the FDR (False Discovery Rate) threshold value considered. Orange = genes involved in the Myc-pathway; Blue = genes involved in the metastatic process; Brown = genes involved in the DNA methylation process; Violet = genes involved in interferon responses; Grey = genes involved in the estrogen-receptor pathway.

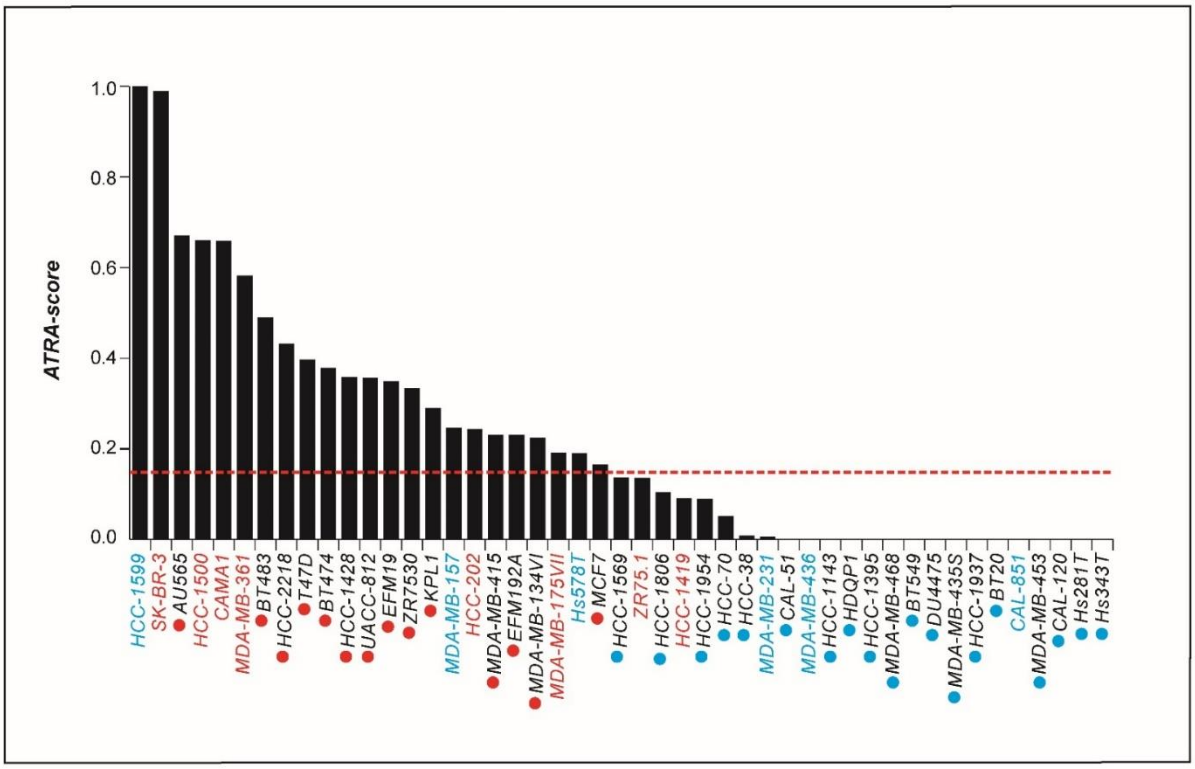

Supplementary Figure S8 *Sensitivity of breast cancer cell-lines to the anti-proliferative activity of ATRA*

The column diagram illustrates the experimental *ATRA-score* values calculated for the indicated cell-lines. The 14 cell-lines belonging to our experimental panel are shown in blue (basal cell-lines) and red (luminal cell-lines). The basal or luminal phenotype of all the other cell-lines is indicated by a blue and a red dot, respectively. The dotted red line represents the threshold *ATRA-score* value (0.17) used to separate the cell-lines binomially in two groups characterized by high and low ATRA-sensitivity.

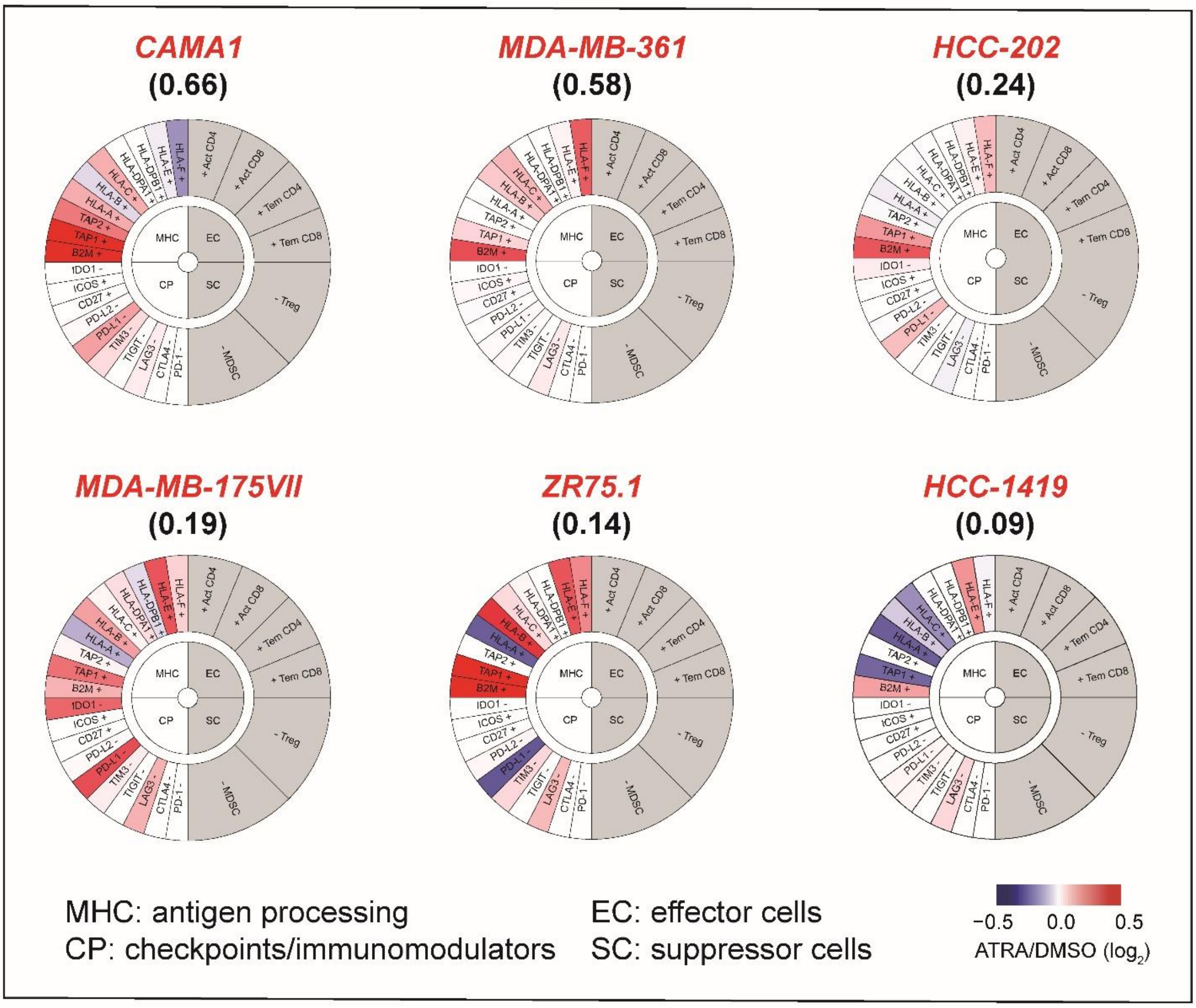

Supplementary Figure S9 *Effects of ATRA on the immunophenograms of luminal cell-lines*
The indicated luminal cell lines were treated with vehicle (DMSO) or ATRA (1 μM) for 24 hours. Total RNA was extracted and subjected to *RNA-seq* analysis. The circles show the effects exerted by ATRA on the immunophenograms derived from the *RNA-seq* data. The *ATRA-score* of each cell-line is indicated in parenthesis.

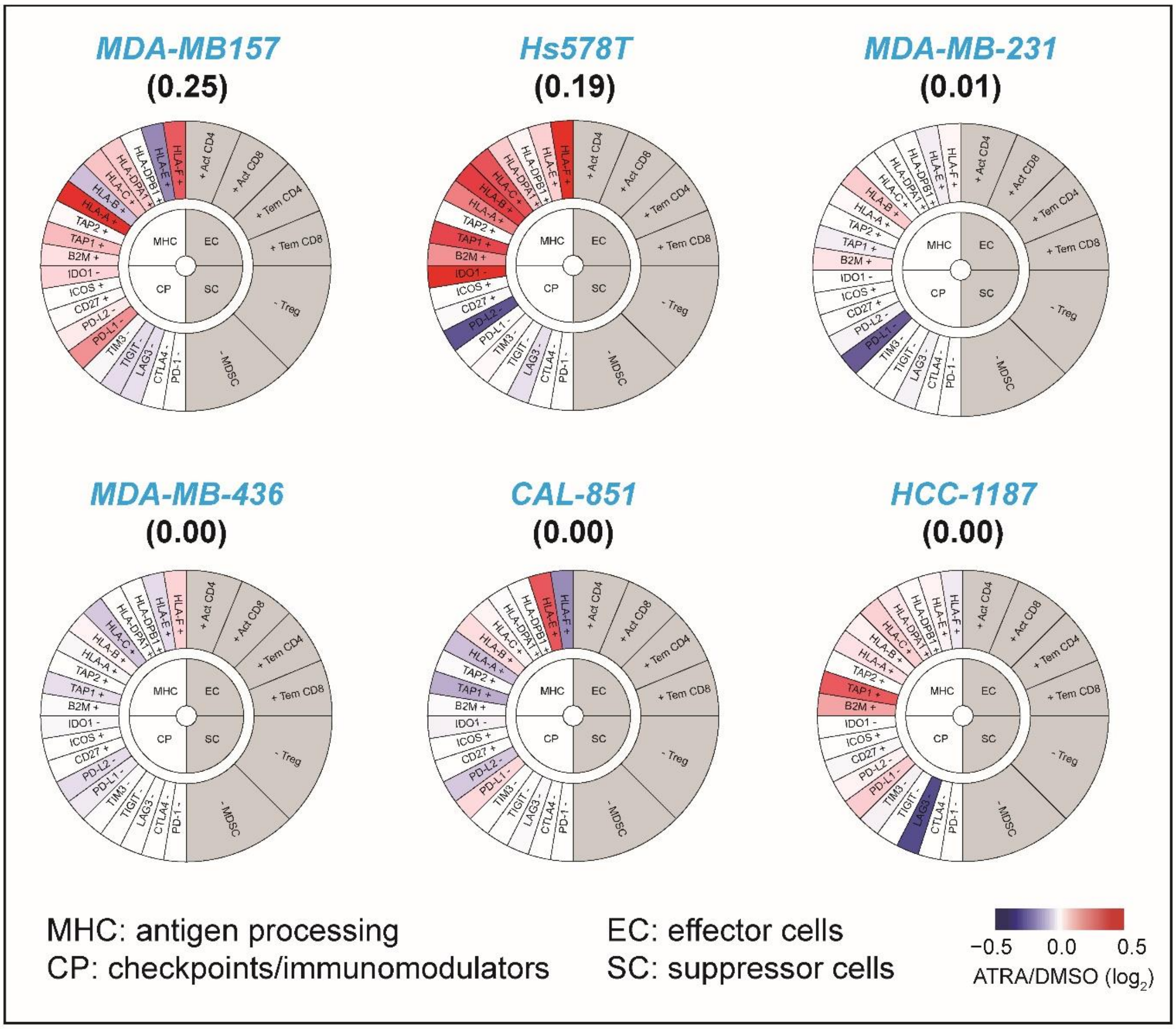

Supplementary Figure S10 *Effects of ATRA on the immunophenograms of basal cell-lines*
The indicated basal cell lines were treated with vehicle (DMSO) or ATRA (1 μM) for 24 hours. Total RNA was extracted and subjected to *RNA-seq* analysis. The circles show the effects exerted by ATRA on the immunophenograms derived from the *RNA-seq* data. The *ATRA-score* of each cell-line is indicated in parenthesis.

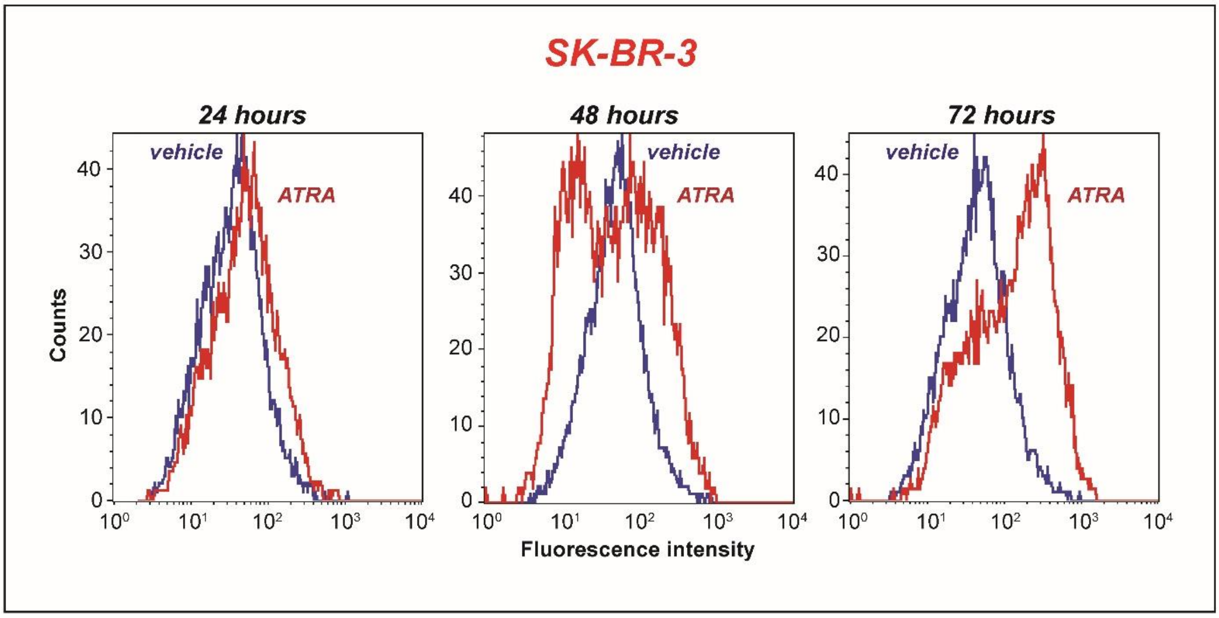

Supplementary Figure S11 *Effects of ATRA on the surface expression of HLA class I antigens in SK-BR-3 cells*

The panel illustrates the FACS (Fluorescence Activated Cell Sorter) analyses of the HLA molecules obtained in retinoid sensitive *SK-BR-3* cells exposed to ATRA for the indicated time frames.

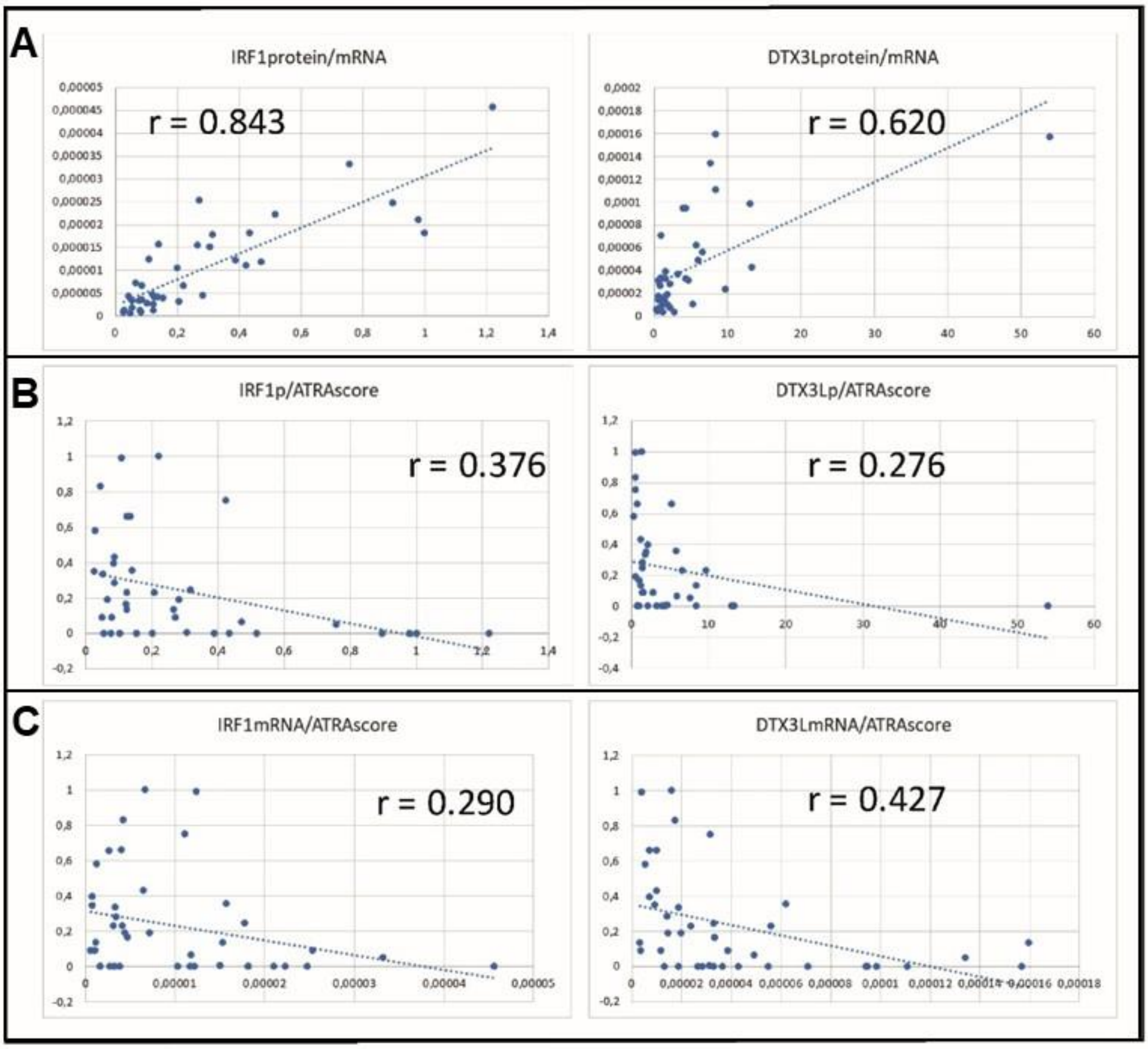

Supplementary Fig. S12 *IRF1 and DTX3L correlations*
The levels of IRF1 and DTX3L proteins as well as mRNAs were determined in 41 breast-cancer cell-lines profiled for their sensitivity to ATRA with the use of the *ATRA-score*. (**A**) The diagrams show the linear correlation plots of IRF1 and DTX3L proteins with the corresponding mRNAs. (**B**) The diagrams show the linear correlation plots of IRF1 and DTX3L proteins with the *ATRA-score* of each cell-line. (**C**) The diagrams show the linear correlation plots of IRF1 and DTX3L mRNAs with the *ATRA-score* of each cell-line.

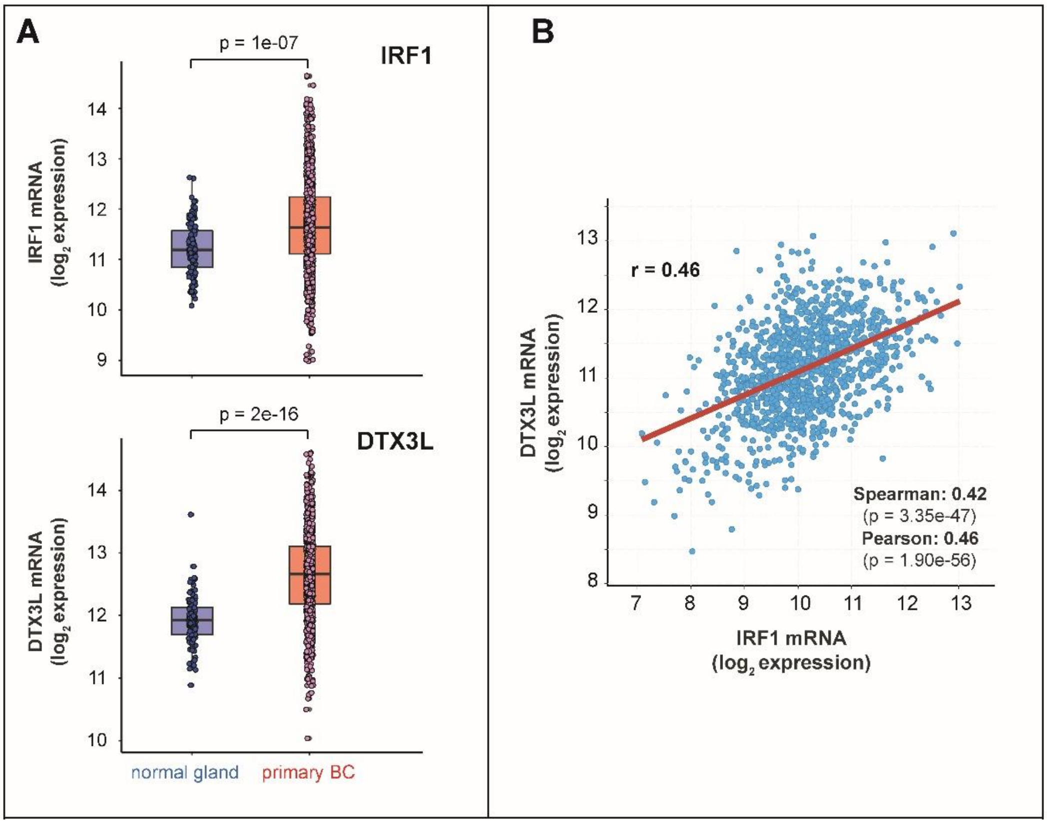

Supplementary Figure S13 *IRF1 and DTX3L mRNA expression in primary mammary tumors*
The levels of IRF1 and DTX3L mRNAs were determined on the basis of the *RNA-seq* data of the normal mammary glands and the primary breast-cancer samples available in the TCGA database. (**A**) The panel shows the mRNA expression levels of IRF1 and DTX3L in the single samples indicated. The p-values were adjusted for the FDR using DESEQ2 analysis pipeline. (**B**) The panel illustrates the correlations between the expression levels of DTX3L and IRF1 mRNAs in breast tumors and the normal mammary gland. The data demonstrate a significant direct correlation between the levels of the two mRNAs.

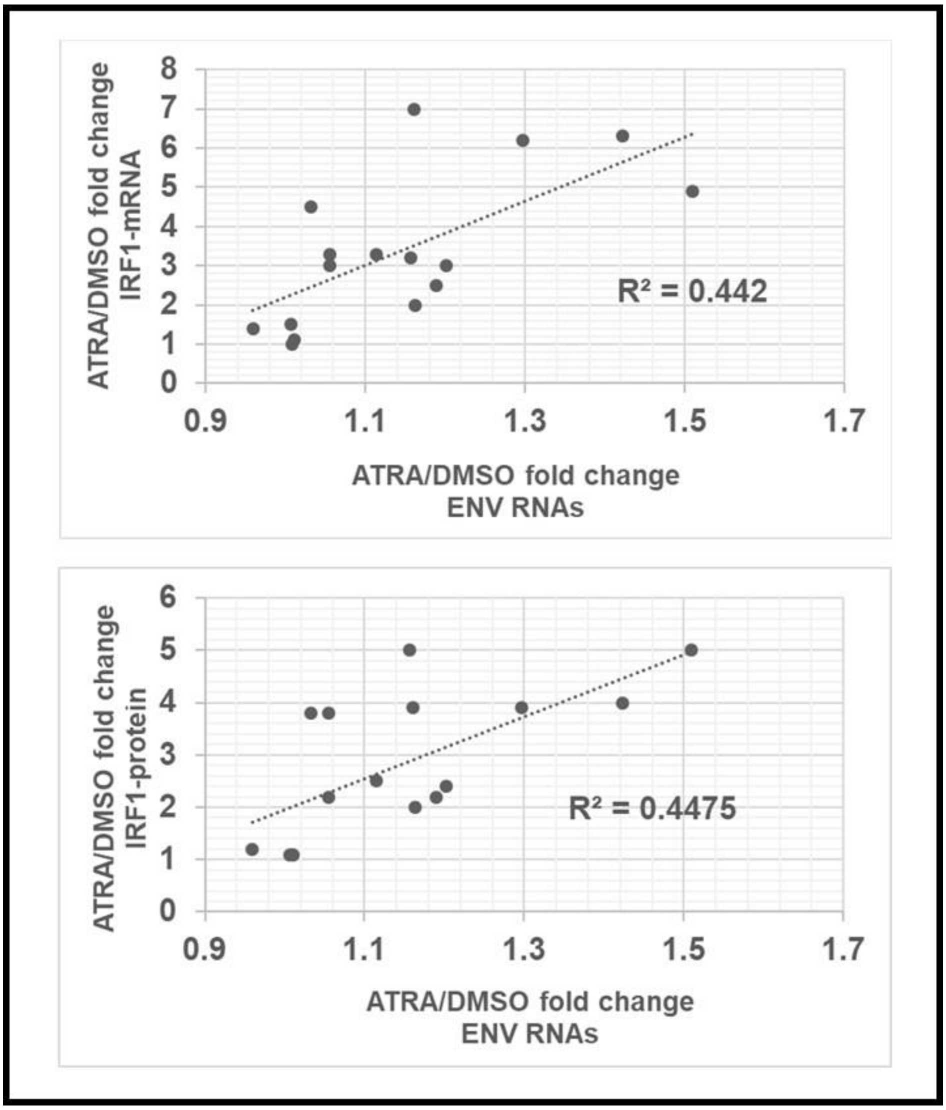

Supplementary Figure S14 *Correlations between ATRA-dependent IRF1 mRNA/protein and endogenous retrovirus induction in breast cancer cell-lines*

The upper diagram illustrates the correlation between the ATRA-dependent induction of the IRF1-mRNA shown in and the corresponding retinoid-dependent induction of ENV (Endogenous Retroviruses) RNAs (see Figures 5B and 7A). The lower diagram illustrates the same type of correlation observed between the ATRA-dependent induction of the IRF1-protein and the corresponding retinoid-dependent induction of ENV (Endogenous Retroviruses) RNAs.

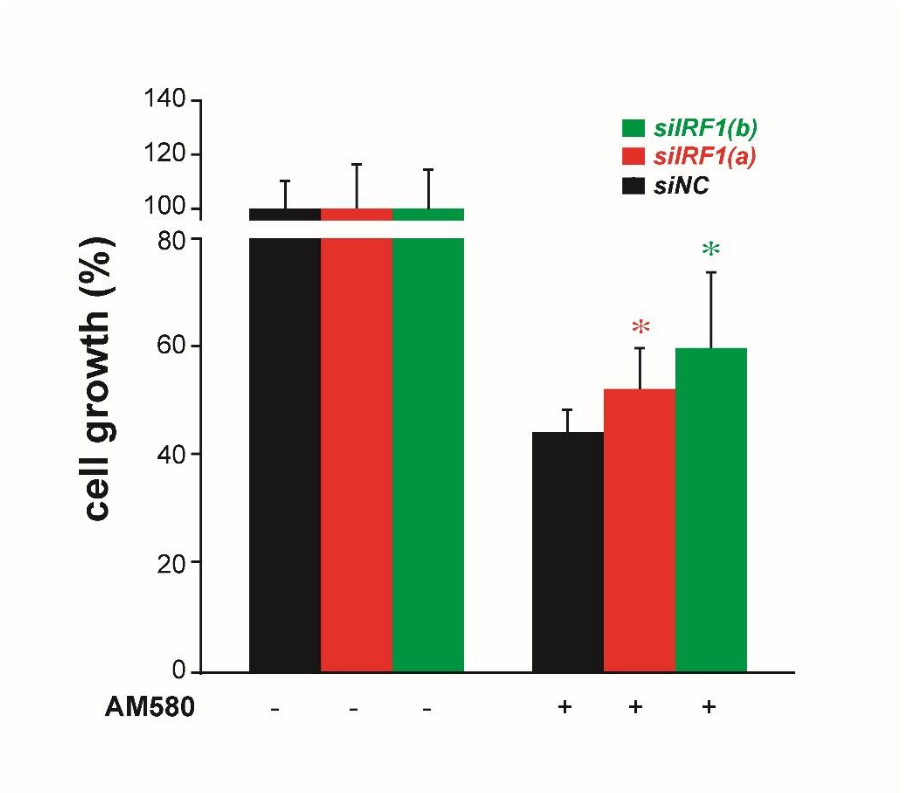

Supplementary Fig. S15 *Effects of IRF1 silencing on AM580-induced growth inhibition in SK-BR-3 cells*

SK-BR-3 cells were transiently transfected with *siIRF1(a)* and *siIRF1(b)*, two siRNAs targeting IRF1, or the scrambled *siNC* control. The indicated cells were treated with the RARα agonist, AM580 (0.1 μM), for 96 hours. Cell growth was evaluated and data were analysed as in (**A**). Values are the mean ± SD of 3 replicate cultures.

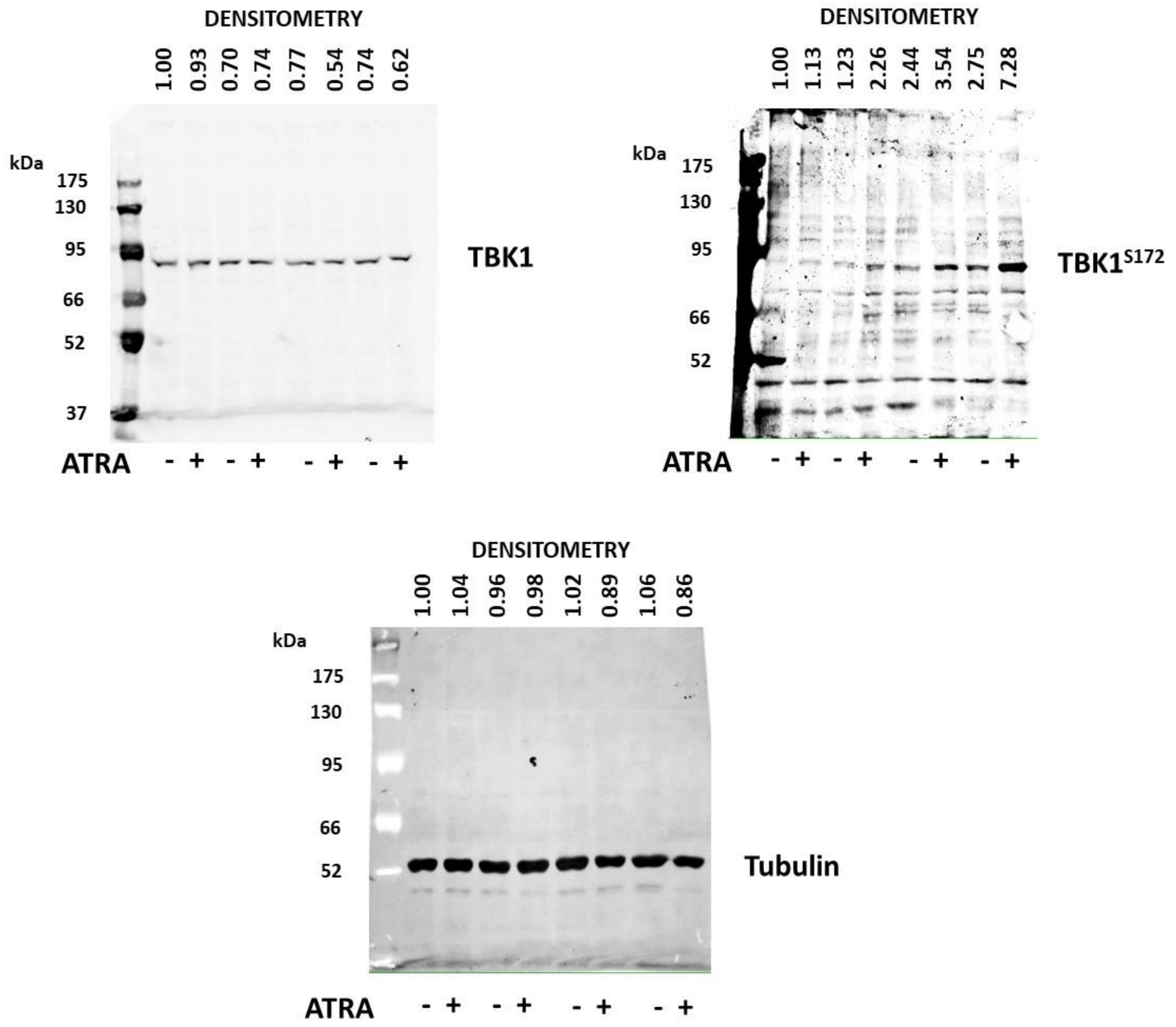

Legend: Original Western blots contained in **Figure 5D**

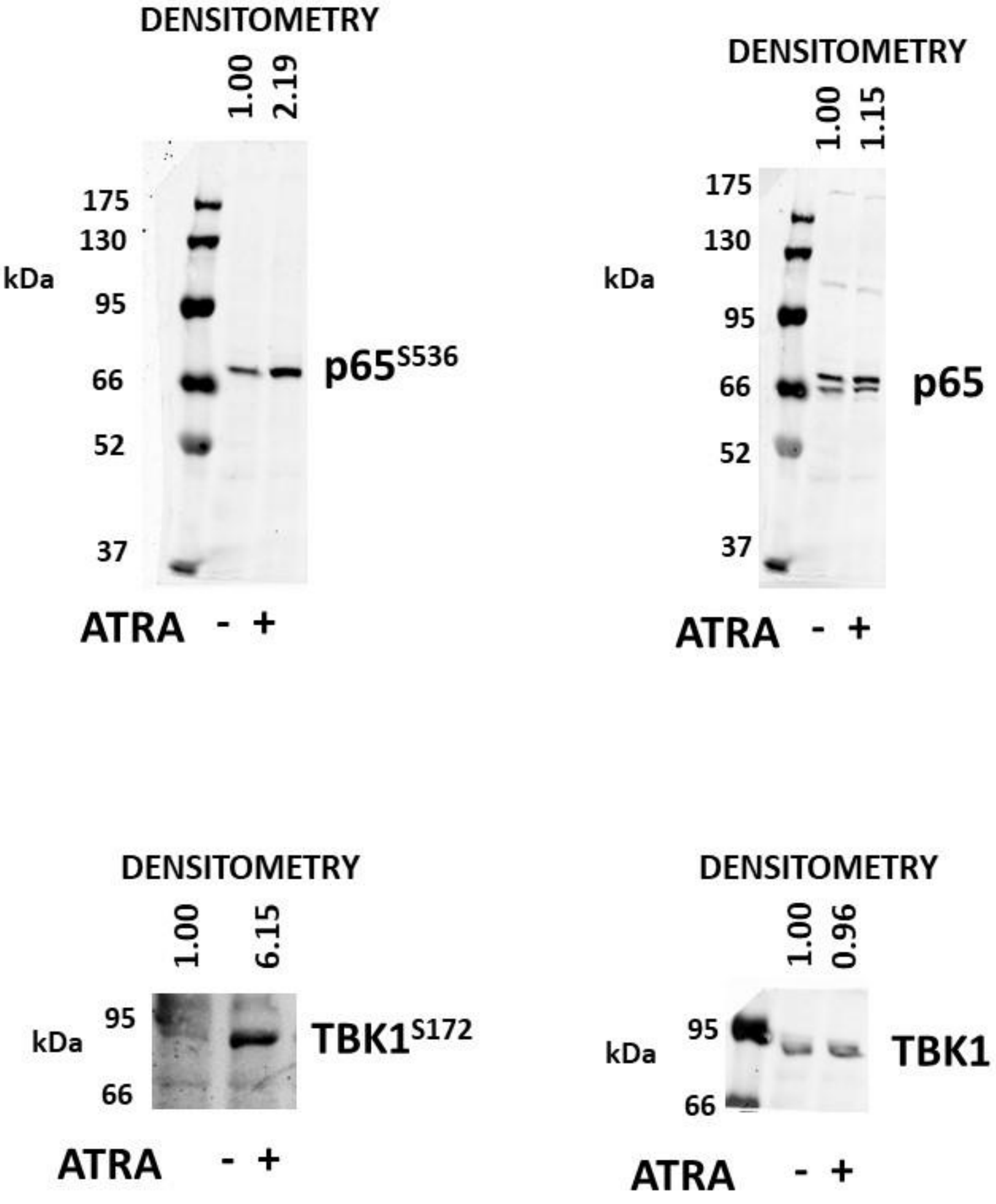

Legend: Original Western blots contained in **Figure 5D**

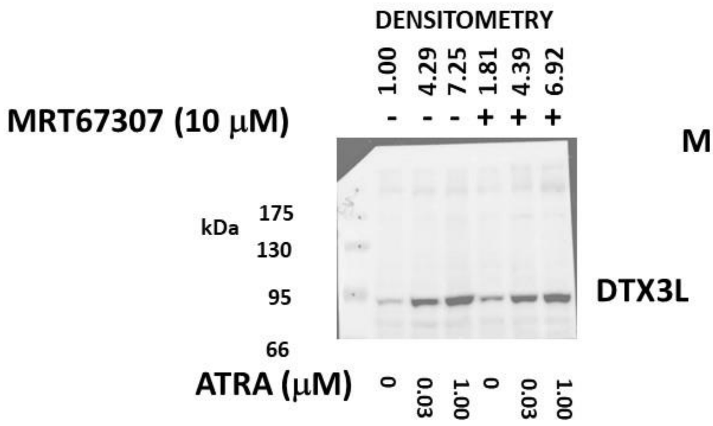

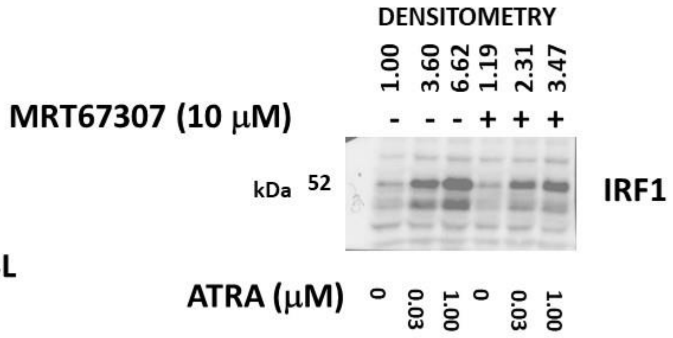

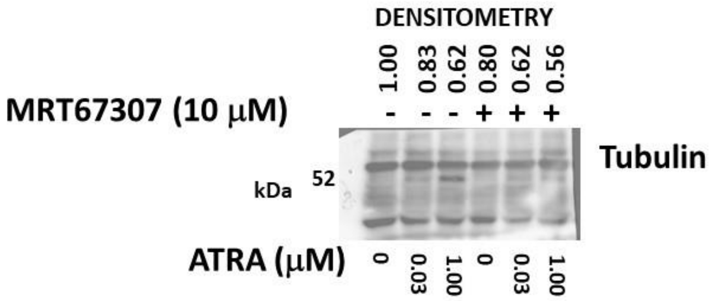

Legend: Original Western blots contained in **Figure 5E**

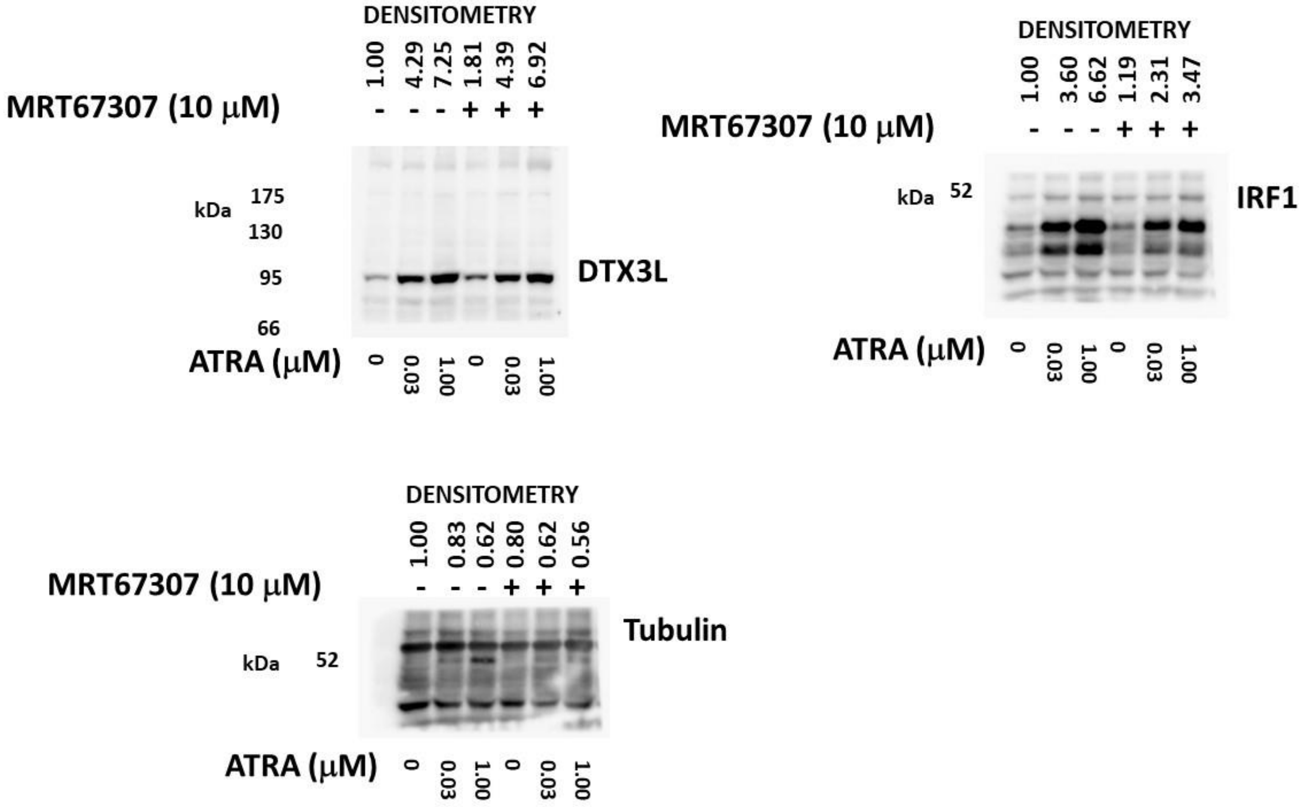

Legend: Original Western blots contained in **Figure 5E**

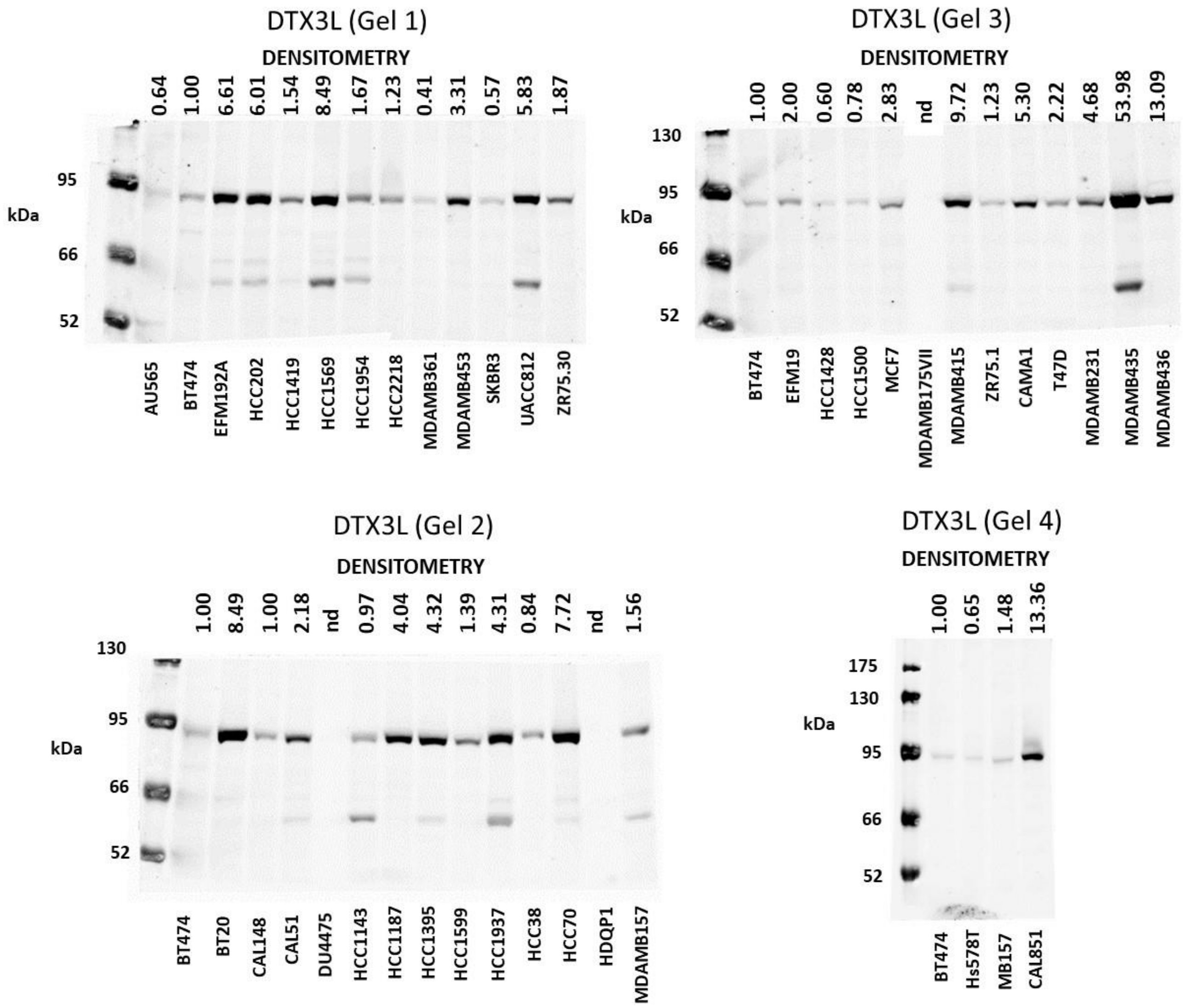

Legend: Original Western blots contained in **Figure 6**

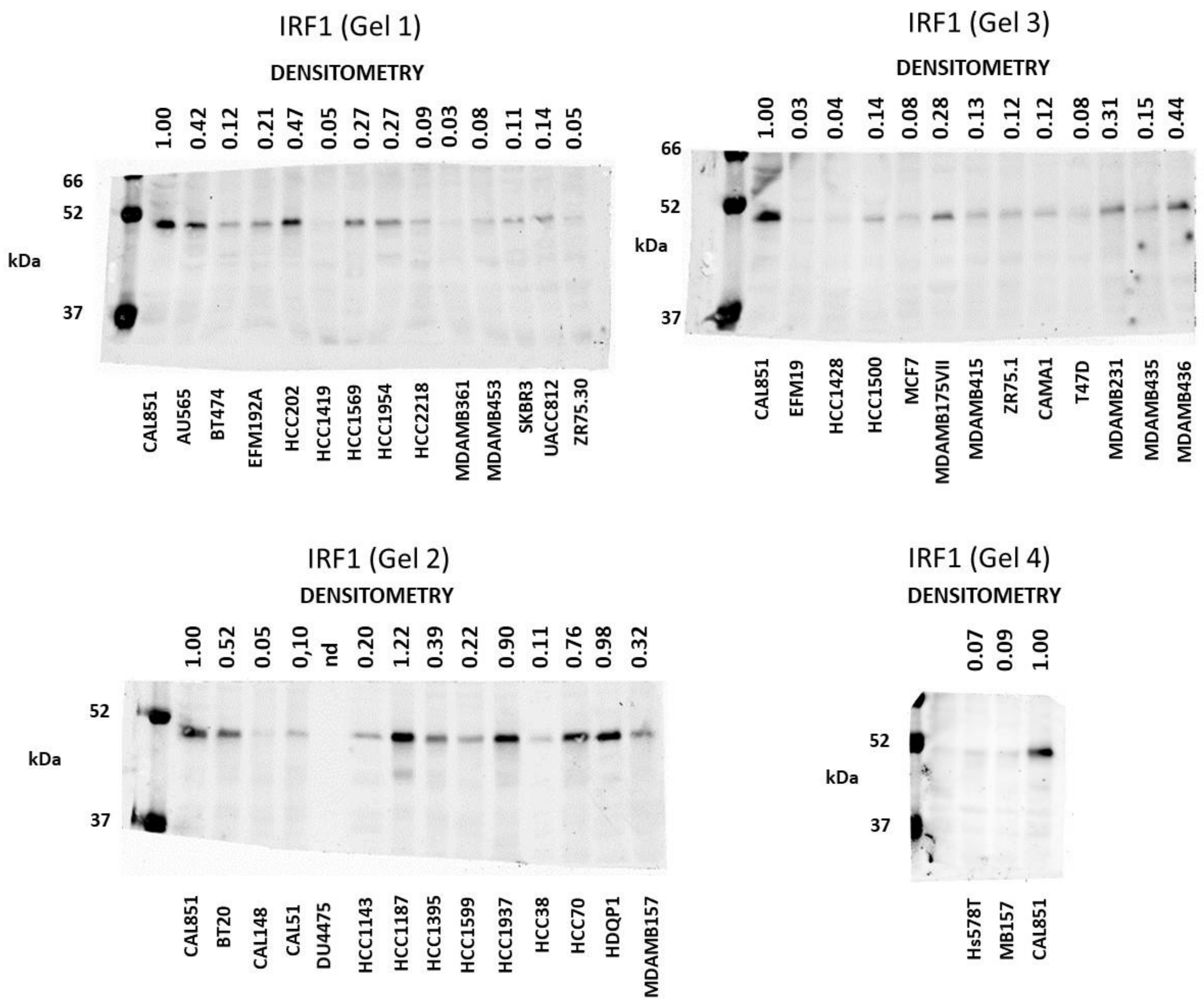

Legend: Original Western blots contained in **Figure 6**

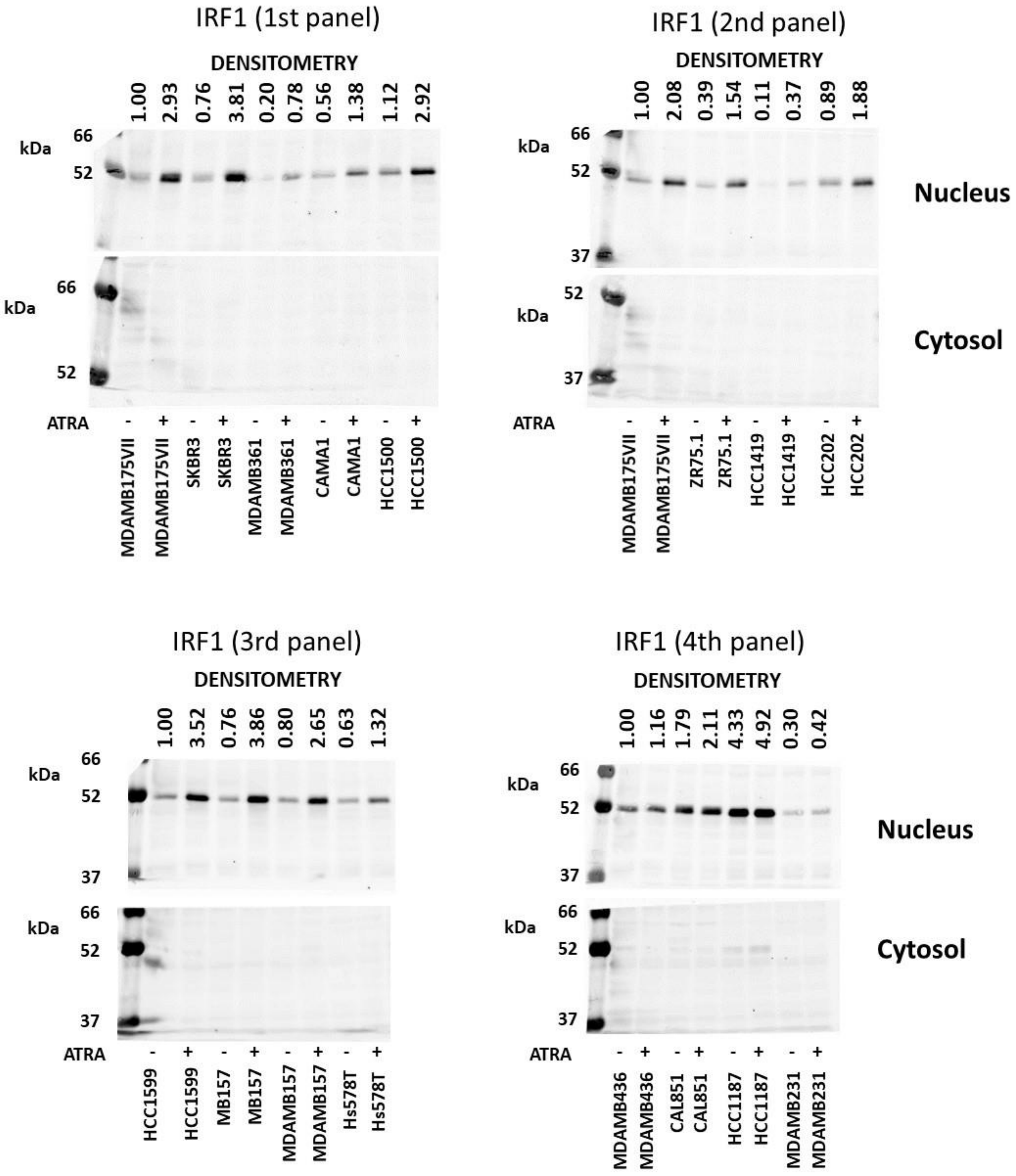

Legend: Original Western blots contained in **Figure 7A**

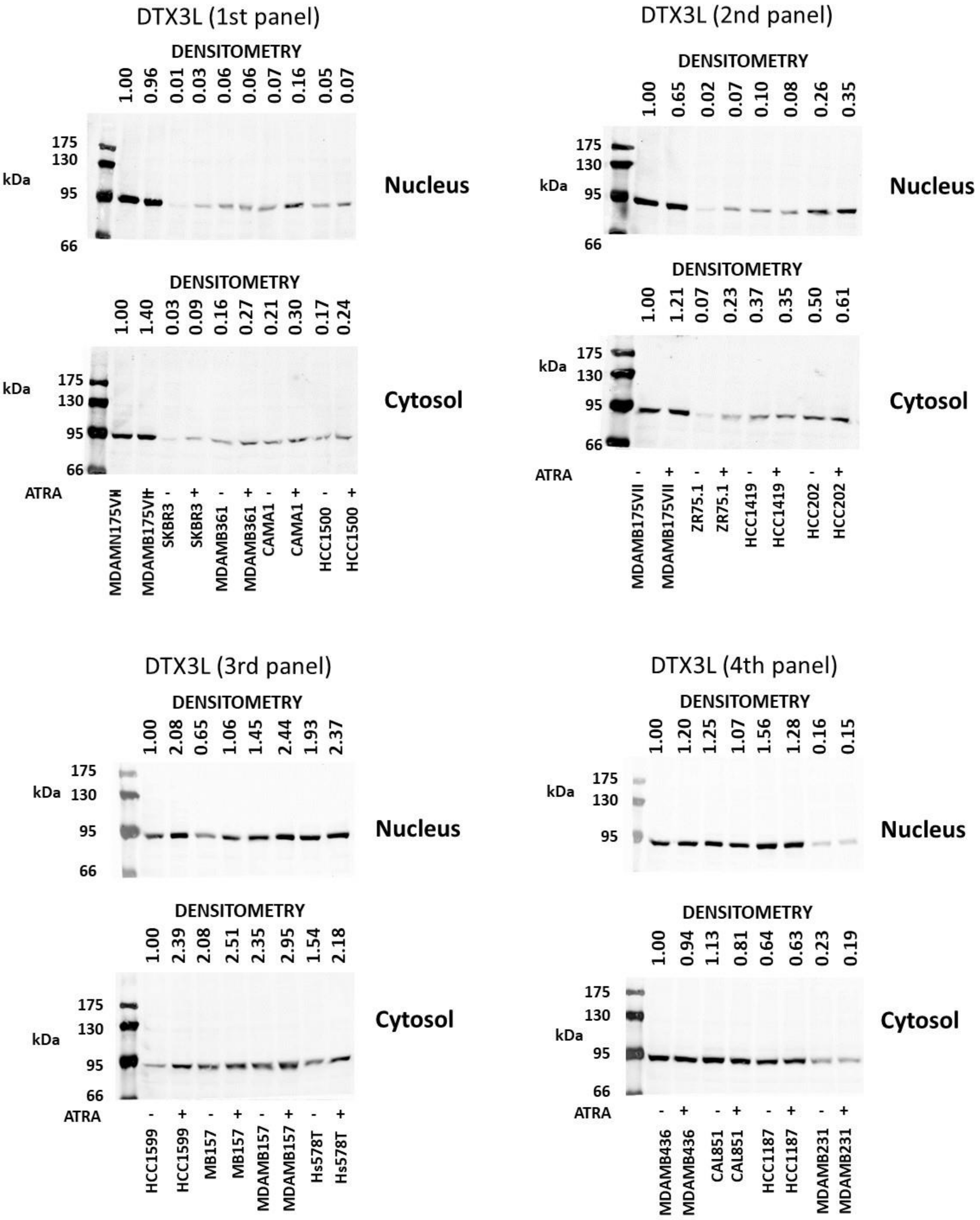

Legend: Original Western blots contained in **Figure 7B**

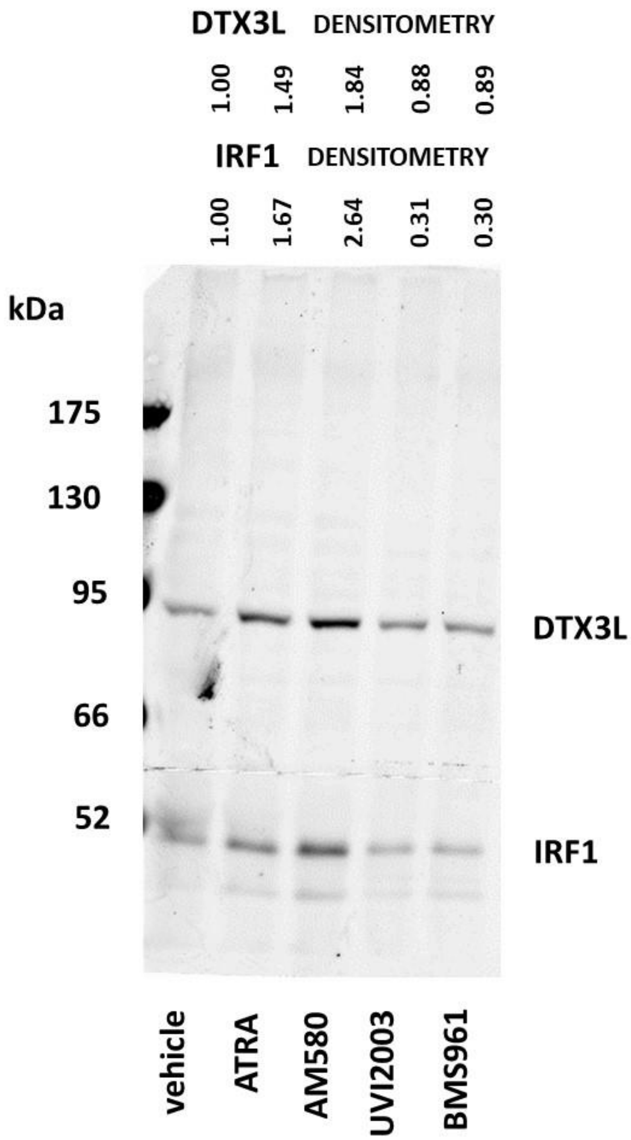

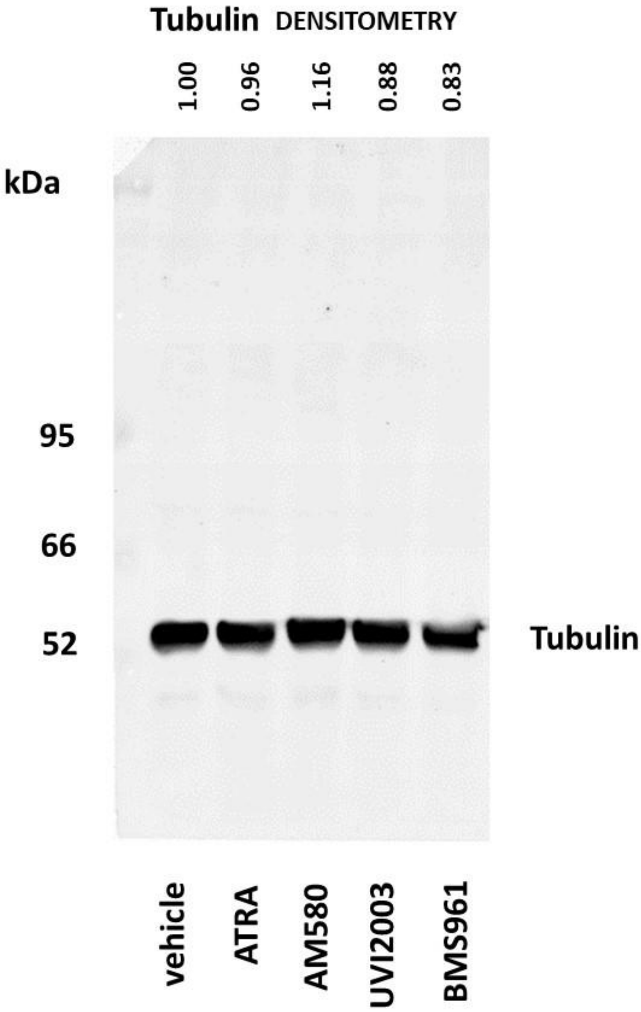

Legend: Original Western blots contained in **Figure 7C**

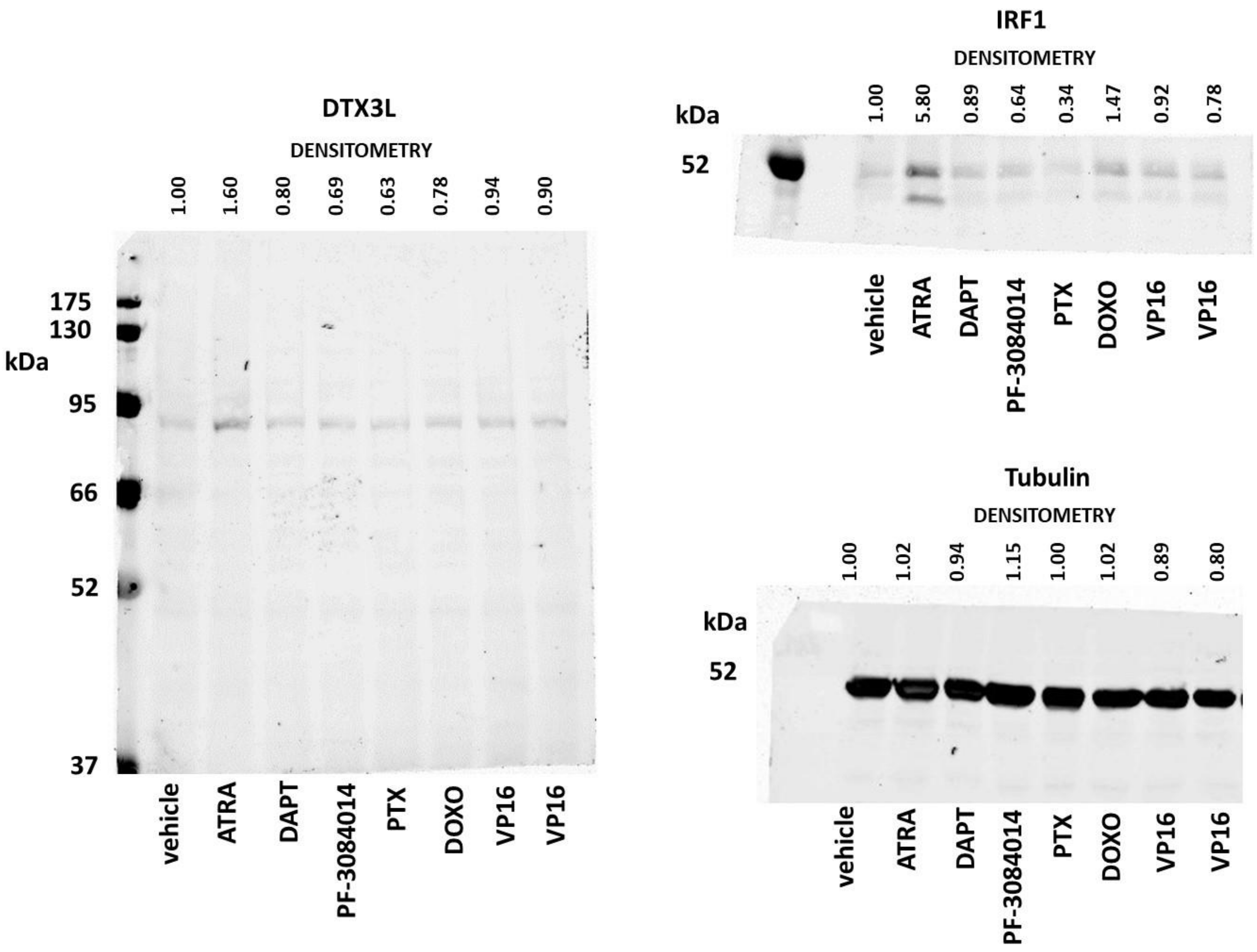

Legend: Original Western blots contained in **Figure 7D**

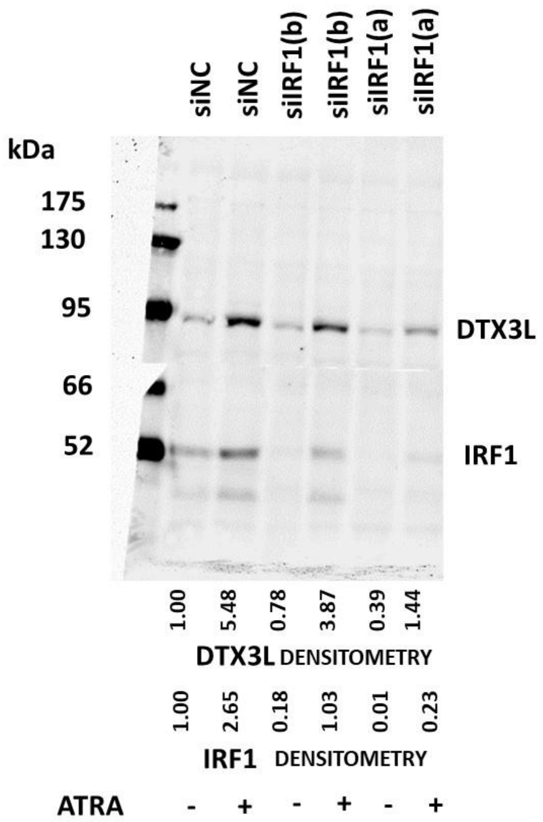

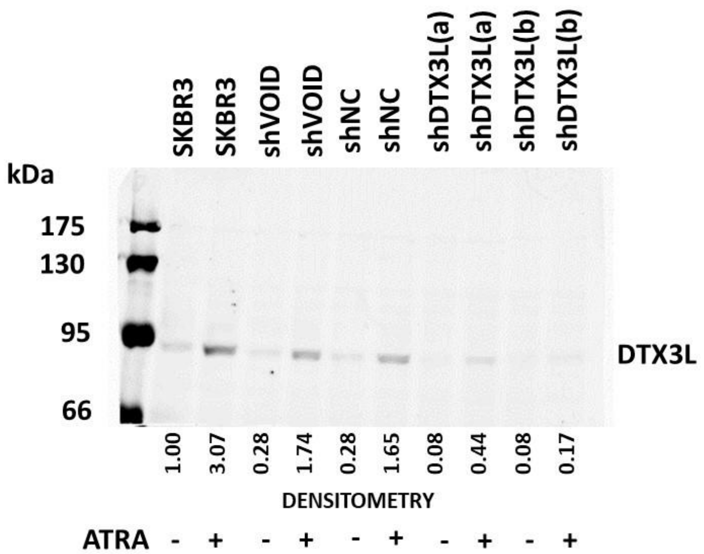

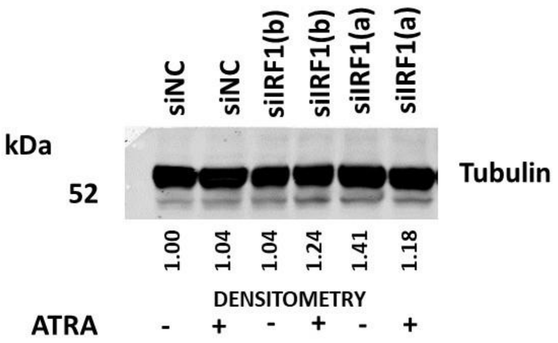

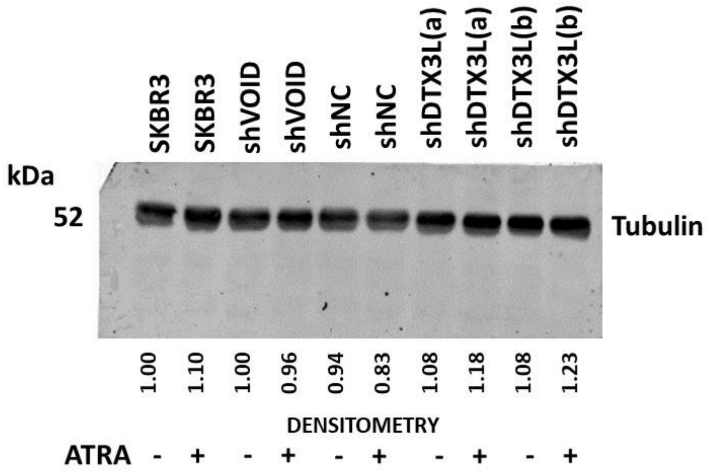

Legend: Original Western blots contained in **Figures 7E** and **7F**
